# WHO global vaccine safety multi-country collaboration project on safety in pregnancy: Assessing the level of diagnostic certainty using standardized case definitions for perinatal and neonatal outcomes and maternal immunization

**DOI:** 10.1016/j.jvacx.2021.100123

**Published:** 2021-11-03

**Authors:** Anke L. Stuurman, Apoorva Sharan, Shubhashri Jahagirdar, Varalakshmi Elango, Margarita Riera-Montes, Neeraj Kashyap, Jorne Biccler, Ramesh Poluru, Narendra Arora, Matthews Mathai, Punam Mangtani, Hugo DeVlieger, Steven Anderson, Barbee Whitaker, Hui-Lee Wong, Clare Cutland, Christine Guillard Maure, WHO GVS MCC Sites

**Affiliations:** a P95 Pharmacovigilance and Epidemiology, Leuven, Belgium; b INCLEN Trust International, New Delhi, India; c Swiss Tropical and Public Health Institute (Swiss TPH), Basel, Switzerland; d University of Basel, Basel, Switzerland; e Centre for Maternal and Newborn Health, Liverpool School of Tropical Medicine, Liverpool, UK; f Department of Infectious Disease Epidemiology, London School of Tropical Medicine, London, UK; g Universitair Ziekenhuis, Leuven, Belgium; h Center for Biologics Evaluation and Research (CBER), U.S. Food and Drug Administration (FDA), Silver Spring, MD, USA; i African Leadership in Vaccinology Expertise (Alive), Faculty of Health Sciences, University of the Witwatersrand, Johannesburg, South Africa

**Keywords:** Vaccination, Pregnancy, Maternal immunization, Vaccine safety, Brighton Collaboration, Standardized case definitions, LMIC

## Abstract

Standardized case definitions strengthen post-marketing safety surveillance of new vaccines by improving generated data, interpretation and comparability across surveillance systems. The Global Alignment of Immunization Safety Assessment in Pregnancy (GAIA) project developed standardized case definitions for 21 key obstetric and neonatal terms following the Brighton Collaboration (BC) methodology.

In this prospective cohort study, we assessed the applicability of GAIA definitions for maternal immunization exposure and for low birth weight (LBW), preterm birth, small for gestational age (SGA), stillbirth, neonatal death, neonatal infection, and congenital microcephaly. We identified the missing data elements that prevented identified cases and exposures from meeting the case definition (level 1–3 of BC diagnostic certainty). Over a one-year period (2019–2020), all births occurring in 21 sites (mostly secondary and tertiary hospitals) in 6 Low Middle Income Countries and 1 High Income Country were recorded and the 7 perinatal and neonatal outcome cases were identified from routine medical records. Up to 100 cases per outcome were recruited sequentially from each site.

Most cases recruited for LBW, preterm birth and neonatal death met the GAIA case definitions. Birth weight, a key parameter for all three outcomes, was routinely recorded at all sites. The definitions for SGA, stillbirth, neonatal infection (particularly meningitis and respiratory infection) and congenital microcephaly were found to be less applicable. The main barrier to obtaining higher levels of diagnostic certainty was the lack of sonographic documentation of gestational age in first or second trimester. The definition for maternal immunization exposure was applicable, however, the highest level of diagnostic certainty was only reached at two sites. Improved documentation of maternal immunization will be important for vaccine safety studies. Following the field-testing of these 8 GAIA definitions, several improvements are suggested that may lead to their easier implementation, increased standardization and hence comparison across studies.

## Highlights

• Applicability of GAIA definitions were assessed using medical records in LMICs.
• LBW, preterm birth and neonatal death definitions were applicable in the field.
• SGA, stillbirth, neonatal infection, congenital microcephaly definitions were less applicable.
• Suggestions to the improvement in GAIA definitions were made.
• Better documentation of maternal immunization is suggested for vaccine-safety studies.

## Introduction

Immunization during pregnancy can protect the pregnant woman and her child, both in the womb and in early life by increasing the antibody titers against vaccine-preventable diseases [1], [2]. Diseases like pertussis, influenza, group B streptococcus (GBS) infection, respiratory syncytial virus (RSV) infection and tetanus have a disproportionate impact on the newborn and young infant. Immunization of pregnant women with Tetanus Toxoid Containing Vaccine through the Maternal and Neonatal Tetanus Elimination (MNTE) initiative, achieved elimination of maternal and neonatal tetanus in 47 countries within twenty years [3]. For tetanus, immunization in pregnancy is a key strategy to prevent significant morbidity and mortality amongst young infants globally. Implementation or strengthening of pertussis and influenza immunization of pregnant women holds great promise as a strategy to protect infants from these infections [4], [5], [6]. This is of specific interest for Low and Middle-Income Countries (LMICs), where access to basic health services may be limited and the burden of vaccine-preventable diseases is large [7].

No evidence of adverse pregnancy outcomes from the vaccination of pregnant women with inactivated virus, bacterial vaccine, or toxoid has been found [8]. Live attenuated vaccines pose a theoretical risk to the fetus, therefore, these vaccines are generally contraindicated during pregnancy [9]. However, potential risks of the vaccine must be balanced against the risk of infection, and in a recent systematic review no evidence of harm related to live attenuated vaccines, other than the smallpox vaccine, was found [10]. Vaccine safety surveillance is complex, especially during pregnancy, when the risk of adverse outcomes may change due to underlying maternal health conditions, quality of obstetric care and the exposure to infection or vaccination during pregnancy [8]. In LMICs, safety monitoring is further challenged by limited pharmacovigilance infrastructure [11]. Post-marketing safety surveillance of new vaccines used during pregnancy is important for the detection of any Adverse Events Following Immunization (AEFIs) in pregnant women and their infants and also to provide information on the potential risks to the fetus [8]. This has become very important now in the context of COVID-19 pandemic, where more safety data are needed for immunizing this vulnerable group.

Enhancing pharmacovigilance capacity is a strategic goal of the WHO’s Global Vaccine Safety Blueprint [12]. In this context, the Global Vaccine Safety Initiative tested the establishment and operationalization of a global network of hospital-based sentinel sites for vaccine safety signal verification in the general population [13]. The present study of safety in pregnancy builds upon lessons learnt from the earlier proof of concept project [14].

The use of standardized case definitions can strengthen programs of immunization in pregnancy, by improving generated data and facilitating data interpretation and comparability across surveillance systems [15]. To this end, the Global Alignment of Immunization Safety Assessment in Pregnancy (GAIA) project, launched in 2015 and managed by the Brighton Collaboration [16], developed standardized case definitions for 21 key obstetric and neonatal terms. Within each case definition, multiple ‘Levels of diagnostic certainty’ are recognized, which take into account current scientific evidence and different levels of diagnostic capacity available in different research and geographic settings [17].

The primary objective of this prospective study was to assess the applicability of GAIA definitions for maternal exposure to vaccination [18] and for seven perinatal and neonatal outcomes (low birth weight (LBW) [19], preterm birth [20], small for gestational age (SGA) [21], stillbirth [22], neonatal death [18], neonatal infection [23], and congenital microcephaly [24]) in LMICs, in order to inform future vaccine safety studies. Specifically, we assessed the proportion of cases or exposures identified that met the GAIA definition at any level of diagnostic certainty and the proportion that could be classified to each level*.* In addition, we identified which missing data elements prevented identified cases and exposures from meeting the definition at the lowest level or a higher level of diagnostic certainty.

## Methods

This was a prospective, descriptive, cohort study using a common protocol (S1) and routinely collected data at 21 sites in six LMICs (Ghana, Tanzania, Zimbabwe, Iran, India, Nepal) and one high-income country (HIC) (Spain), consisting of one primary care center, five secondary hospitals and fifteen tertiary hospitals. Each site had a maternity ward. Sites were selected using a 2017 study that employed site selection criteria and acceptable performance in a simulation exercise that tested capacity to access sufficient data of acceptable quality at the site level [25]. Table 1 lists characteristics of the participating sites. The first site started data collection on May 6, 2019, and the last site completed data collection on August 18, 2020. Detailed methods are available in the report [26].

**Table 1:** Characteristics of participating sites.

Country	Site	Type of healthcare setting (primary, secondary, tertiary)	Facility ownership (public, private)	Presence of NICU	Records (paper, electronic, combination)	Number of births during the one-year study period, n
*AFRO*						
Ghana	St Joseph's H	Secondary	Public	Yes	Paper	1634
Ghana	Ejisu H	Secondary	Public	Yes	Paper	1442
Ghana	Tema GH	Secondary	Public	Yes	Paper	5523
Ghana	Eastern RH	Secondary	Public	Yes	Combination	5387
Tanzania	Mbeya ZRH	Tertiary	Public	No	Combination	7023
Tanzania	St Francis RH	Tertiary	Public Private Partnership	No	Paper	3484
Tanzania	Mbeya RRH	Tertiary	Public	Yes	Paper	3930
Zimbabwe	Mbare PC	Primary	Public	No	Paper	5501
Zimbabwe	Mutare PH	Tertiary	Public	No	Paper	1558
*EMRO*						
Iran	Mahdieh H	Tertiary	Public	Yes	Combination	5802
Iran	Shohada TH	Tertiary	Public	Yes	Combination	864
*EURO*						
Spain	General Castellon UH	Tertiary	Public	Yes	Electronic	1390
Spain	Dr Peset UH	Secondary	Public	No	Electronic	1078
*SEARO*						
India	JSS H	Tertiary	Private	Yes	Combination	2796
India	Grant GMC	Tertiary	Public	Yes	Paper	2251*
India	IMS SUM H	Tertiary	Private	Yes	Combination	1805
India	Kasturba MC	Tertiary	Private	Yes	Combination	2762
India	MP Shah MC	Tertiary	Public	Yes	Paper	9996
India	SKIMS	Tertiary	Public	Yes	Paper	3188
Nepal	Patan H	Tertiary	Public	Yes	Paper	7573
Nepal	BP Koirala	Tertiary	Public	Yes	Combination	10,554

*8 months instead of 1 year at Grant GMC.

AFRO: WHO African region; BP: BP Koirala Institute of Health Sciences; EMRO: WHO Eastern Mediterranean region; EURO: WHO European region; GH: General Hospital; GMC: Government Medical College; GUH: General University Hospital; H: Hospital; IMS SUM: Institute of Medical Science and Sum Hospital; MC: Medical College; NICU: neonatal intensive care unit; PC: Policlinic; PH: Provincial Hospital; RH: Referral/Regional Hospital; RRH: Regional Referral Hospital; SEARO: WHO South-East Asia region; SKIMS: Sher-i-Kashmir Institute of Medical Sciences; TH: Teaching Hospital; UH: University Hospital; ZRH: Zonal Referral Hospital.

### Case identification, recruitment and data collection

All births at the sites were prospectively recorded during a one-year period, and the following study outcomes occurring in the 28 days following birth were identified as part of routine care by the sites: LBW, preterm birth, SGA, stillbirth (antepartum or intrapartum), in-hospital neonatal death, neonatal infection (invasive bloodstream infection (BSI), respiratory infection or meningitis) and postnatally diagnosed congenital microcephaly. The outcomes were selected based on relevance in vaccine safety research and perceived ability to collect data on the outcome of interest. Cases were first identified by screening relevant data sources from the maternity and neonatal wards at the sites (e.g. labor room register, admission register, patient records; see full list in S2). Only study outcomes at the site were considered; no follow-up outside of the site was performed. Estimated rates of occurrence will be reported in a separate paper and are also accessible in the study report [26]. At each site, up to 100 cases of each study outcome were systematically recruited into the study (the first two cases per week, or all consecutive cases); informed consent was obtained from the mother. One hundred cases per outcome per site enabled the calculation of 20% relative precision around estimates of the proportion of cases meeting the GAIA definition, under the assumption that 50% of all cases met at least the lowest level definition. Exhaustive case report forms, including details on any vaccines received during pregnancy, were completed for recruited cases, based on existing routine medical records. Data sources included the mother’s antenatal care records, the antenatal care card, and inpatient records (full list in S2).

Study site staff were trained on the study procedures. All the data were captured through an app-based electronic data capture system, SOMAARTH III [27] using tablets. Data quality was monitored centrally, and on-site monitoring visits and regular tele-conferences with sites were conducted.

### Statistical analysis

We developed algorithms for the GAIA definitions for LBW [19], preterm birth [20], SGA [21], stillbirth (antepartum and intrapartum stillbirth) [22], neonatal death [18], neonatal infection (bloodstream infection (BSI), respiratory infection, meningitis) [23], postnatally diagnosed congenital microcephaly [24] and maternal immunization [18] to assess the level of diagnostic certainty of the GAIA definition met by recruited cases, if any. Cases classified as Level 1, 2 or 3 were said to meet the GAIA definition. Level 1 represented the highest levels of diagnostic certainty (most specific, least sensitive), and Level 3 the lowest (least specific, most sensitive). Levels 4 and 5, if present, were not considered as those events did not meet the case definition. The GAIA definitions have been summarized in S3a. First, it assessed whether Level 1 criteria were met. If yes, then the case was considered classified to Level 1. If no, it assessed whether Level 2 criteria were met, and so on. For each definition, the applicability was assessed by calculating the proportion of cases or maternal immunization exposures meeting the GAIA definition and the proportion classified to each level, by site. The most common reasons for not meeting GAIA definitions or, for non-classification of level 3 cases to levels 1–2 were summarized (or described) for each outcome.

We modified the GAIA definition so that criteria accepted at higher levels of diagnostic certainty (‘higher levels of evidence’) were also de facto acceptable at lower levels of diagnostic certainty. For example, in the case of LBW, we considered electronic scales (sufficient for levels 1 and 2) appropriate for a level 3 classification as well (S3b for further details). Several aspects of the maternal immunization definition were open to interpretation. For level 1, we interpreted ‘date/time’ as ‘date AND time’ and ‘details of vaccine’ as ‘lot number AND EITHER name of disease OR name of vaccine’. For level 2, we interpreted ‘details of disease’ as ‘name of the disease OR name of vaccine OR lot number’. For levels 1–2, primary sources such as the antenatal care card, vaccine card or vaccine register were required, and for level 3, secondary sources were accepted such as the patient case sheet or birth register.

For each outcome, the proportion of recruited cases that met the GAIA definition was stratified by country, health facility level (primary, secondary, tertiary/referral), and health facility ownership (public/private). A Chi-square test was used to assess whether there were any significant differences between the categories.

Double independent programming of all analyses was performed using R version 3.6.0 [28] by the company, P95 Epidemiology and Pharmacovigilance and Stata version 15.1 [29] by INCLEN Trust International. Output was compared and the differences were resolved.

### Ethics approval

The study was approved by the WHO Ethics Review Committee (protocol ID: ERC.0003114), and by local and national committees as appropriate [26].

## Results

Table 2 shows the number of cases recruited for each outcome by site. The number of cases recruited was highest for LBW and preterm birth, and lowest for meningitis, respiratory infection and congenital microcephaly. Table 3 shows exposure to (any) vaccination during pregnancy for each site. The median percentage of mothers identified by the sites as vaccinated during pregnancy was 82.4% (interquartile range (IQR): 61.2–88%); the exposure status was reported as unknown for 16.2% (median) of mothers (IQR: 5.6–31.7%).

**Table 2:** Number of cases recruited for each outcome by site.

					Cases recruited, n						
Country	Site	LBW	Preterm birth	SGA	Stillbirth		Neonatal death	Neonatal infection			Congenital microcephaly
					Antepartum	Intrapartum		BSI	Meningitis	Respiratory infection	
*AFRO*											
Ghana	St Joseph's H	100	88	82	16	13	25	63	0	12	2
Ghana	Ejisu H	59	27	18	5	7	0	15	0	0	1
Ghana	Tema GH	101	100	99	6	93	63	61	0	6	3
Ghana	Eastern RH	101	100	100	93	7	95	49	3	0	2
Tanzania	Mbeya ZRH	109	95	83	81	21	83	23	2	2	1
Tanzania	St Francis RH	100	98	- *	51	46	63	65	4	4	- *
Tanzania	Mbeya RRH	100	97	99	57	29	59	79	2	2	3
Zimbabwe	Mbare PC	106	76	74	3	8	8	8	0	0	0
Zimbabwe	Mutare PH	101	99	86	40	10	66	6	0	1	0
*EMRO*											
Iran	Mahdieh H	100	100	102	77	9	69	50	0	11	28
Iran	Shohada TH	73	92	16	19	4	14	46	1	5	7
*EURO*											
Spain	General Castellon UH	104	100	57	NA	NA	3	7	1	NA	11
Spain	Dr Peset UH	47	45	48	NA	NA	0	7	0	NA	14
*SEARO*											
India	JSS H	100	99	85	10	11	14	53	2	5	9
India	Grant GMC	98	96	24	34	16	28	4	2	0	0
India	IMS SUM H	100	99	98	58	NA	7	24	3	0	6
India	Kasturba MC	101	100	100	29	4	28	59	3	2	11
India	MP Shah MC	118	97	91	70	23	52	61	1	11	3
India	SKIMS	100	101	34	35	8	32	34	0	NA	1
Nepal	Patan H	101	101	66	35	5	15	15	22	65	3
Nepal	BP Koirala	131	104	105	88	12	27	54	11	29	15
Total		2050	1914	1467	807	326	751	783	57	155	120

More than 100 cases at some sites were due to overlapping case definition requirements.

* SGA and congenital microcephaly are not routinely diagnosed at St Francis RH.

AFRO: WHO African region; BP: BP Koirala Institute of Health Sciences; BSI: bloodstream infection; EMRO: WHO Eastern Mediterranean region; EURO: WHO European region; GH: General Hospital; GMC: Government Medical College; GUH: General University Hospital; H: Hospital; IMS SUM: Institute of Medical Science and Sum Hospital; MC: Medical College; NA: Not applicable; NICU: neonatal intensive care unit; PC: Policlinic; PH: Provincial Hospital; RH: Referral/Regional Hospital; RI: respiratory infection; RRH: Regional Referral Hospital; SEARO: WHO South-East Asia region; SKIMS: Sher-i-Kashmir Institute of Medical Sciences; TH: Teaching Hospital; UH: University Hospital; ZRH: Zonal Referral Hospital.

**Table 3:** Number of mothers assessed for exposure to (any) vaccination during pregnancy, by site.

Country	Site	Exposure to vaccination assessed, n	Exposed, n (%)	Unexposed, n (%)	Exposure unknown, n (%)
*AFRO*					
Ghana	St Joseph's H	284	250 (88)	27 (9.5)	7 (2.5)
Ghana	Ejisu H	94	60 (63.8)	13 (13.8)	21 (22.3)
Ghana	Tema GH	425	78 (18.4)	12 (2.8)	335 (78.8)
Ghana	Eastern RH	428	306 (71.5)	65 (15.2)	57 (13.3)
Tanzania	Mbeya ZRH	375	67 (17.9)	6 (1.6)	302 (80.5)
Tanzania	St Francis RH	343	210 (61.2)	56 (16.3)	77 (22.4)
Tanzania	Mbeya RRH	386	329 (85.2)	30 (7.8)	27 (7)
Zimbabwe	Mbare PC	183	106 (57.9)	19 (10.4)	58 (31.7)
Zimbabwe	Mutare PH	253	211 (83.4)	26 (10.3)	16 (6.3)
*EMRO*					
Iran	Mahdieh H	402	17 (4.2)	45 (11.2)	340 (84.6)
Iran	Shohada TH	188	0 (0)	128 (68.1)	60 (31.9)
*EURO*					
Spain	General Castellon UH	161	138 (85.7)	14 (8.7)	9 (5.6)
Spain	Dr Peset UH	86	76 (88.4)	4 (4.7)	6 (7)
*SEARO*					
India	JSS H	255	196 (76.9)	4 (1.6)	55 (21.6)
India	Grant GMC	204	168 (82.4)	3 (1.5)	33 (16.2)
India	IMS SUM H	270	268 (99.3)	2 (0.7)	0 (0)
India	Kasturba MC	285	235 (82.5)	0 (0)	50 (17.5)
India	MP Shah MC	436	412 (94.5)	10 (2.3)	14 (3.2)
India	SKIMS	271	263 (97)	7 (2.6)	1 (0.4)
Nepal	Patan H	298	193 (64.8)	0 (0)	105 (35.2)
Nepal	BP Koirala	373	370 (99.2)	2 (0.5)	1 (0.3)

AFRO: WHO African region; BP: BP Koirala Institute of Health Sciences; EMRO: WHO Eastern Mediterranean region; EURO: WHO European region; GH: General Hospital; GMC: Government Medical College; GUH: General University Hospital; H: Hospital; IMS SUM: Institute of Medical Science and Sum Hospital; MC: Medical College; NICU: neonatal intensive care unit; PC: Policlinic; PH: Provincial Hospital; RH: Referral/Regional Hospital; RRH: Regional Referral Hospital; SEARO: WHO South-East Asia region; SKIMS: Sher-i-Kashmir Institute of Medical Sciences; TH: Teaching Hospital; UH: University Hospital; ZRH: Zonal Referral Hospital.

**Fig. 1:**
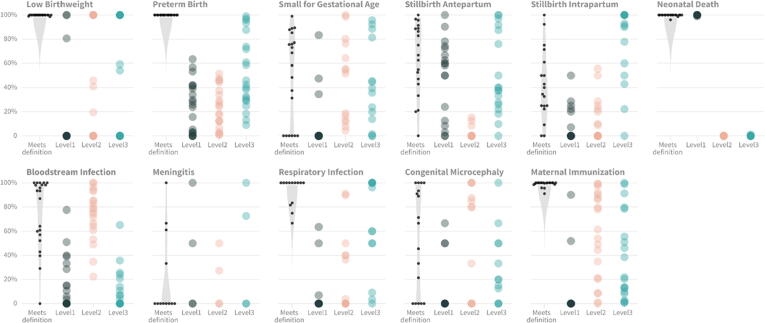
Applicability of GAIA definitions at the study sites. Study sites are represented by dots (column 1) and circles (columns 2–4). Column 1 (‘meets definition’) shows, for each outcome, the distribution of the percentage of recruited cases (or, for maternal immunization, identified exposures) that met the GAIA definition across the sites. Columns 2–4 show the distribution of the percentage of cases classified at level 1 (most specific), 2, and 3 (least specific), among cases that met the GAIA definition across the sites. For each individual site, columns 2–4 sum to 100%. The darker the circle, the larger the number of sites.

The site-specific percentage of cases or exposures that met the GAIA definition and, among those, the percentage that were classified to levels 1–3 have been summarized for all study outcomes in Fig. 1. Further, median and site-specific results are also available in S4. Differences in percentage of cases meeting the case definition by country, health facility level and public vs private ownership are available in the report [26].

*LBW:* Nearly 100% of recruited cases with LBW across the sites met the case definition (median: 100%; IQR: 100–100%). At most sites, all the cases within one site were classified to a single level of diagnostic certainty due to the standard operating procedures for birthweight measurements at the sites.

*Preterm birth:* All recruited preterm birth cases met the GAIA definition. At most sites, classified cases were spread over the three levels of diagnostic certainty. At least 10% of the cases at each site were classified at level 3.

*SGA:* The percentage of SGA cases that met the GAIA definition varied widely among the sites (median: 63.5%, IQR: 0–79%). At six sites (out of 20, one site did not routinely diagnose SGA) no cases met the definition. The majority of the classified cases were classified to either level 2 or 3.

*Stillbirth:* Stillbirths were recruited at all sites, except for one site in Spain where no stillbirths were observed during the study period. At all but two sites part of the recruited stillbirth cases met the GAIA definition; all sites had cases that did not meet the GAIA definition. The median percentage of stillbirth cases that met the GAIA definition across sites was 66.7% (IQR: 45–89%) for antepartum and 37.3% (IQR: 25–58%) for intrapartum stillbirths. Most classified antepartum stillbirths were classified as level 1 or level 3 and the majority of intrapartum stillbirths were classified as level 3.

*Neonatal Death:* Nearly all recruited neonatal death cases met the GAIA definition (median: 100%; IQR: 100–100%). All but one of the classified cases were classified as Level 1. A single case was classified as level 3.

*Neonatal Infection:* At all but one site, part of the neonatal infection cases met the GAIA definition. All sites recruited BSI cases. The median percentage of recruited BSI cases that met the GAIA definition across sites was 93% (IQR: 57–98%). At one site no cases met the GAIA definition. Level 1 was reached for some of the cases at 11 sites. At 16 sites, most classified cases were of level 2. Thirteen sites recruited meningitis cases, less than a quarter of all cases met the GAIA definition (median across sites 0%; IQR: 0–33%). Two cases were classified to level 1, four to level 2 and nine cases to level 3; 49 cases were not classified. Thirteen sites recruited cases of neonatal respiratory infection, the median percentage of cases that met the GAIA definition across sites was 100 % (IQR: 83–100%). Most classified cases were classified to level 3.

*Congenital Microcephaly:* Seventeen sites (out of 20, one site did not routinely diagnose congenital microcephaly) recruited cases of congenital microcephaly. At five of these sites none of the cases met the GAIA definition. The median percentage of recruited congenital microcephaly cases that met the case definition was 67% (IQR: 0–93%). Most cases were classified to level 2, with fewer cases meeting level 1 or 3.

*Maternal immunization:* The percentage of identified maternal immunization exposures that were classified using the GAIA definition was close to 100%. The majority of exposures were classified at levels 2 and 3, level 1 was reached by two sites.

### Reasons for not meeting levels of diagnostic certainty

The reasons for not meeting the GAIA definition to at least level 3 (least specific) were definition-specific (the most frequent reasons are summarized in Table 4, full details are available in S4). A recurrent limiting factor for level 3 cases not meeting levels 1–2 was the GA assessment, particularly that no or insufficient information on 1st or 2nd trimester ultrasound was available. For maternal immunization, the most important reason for not meeting levels 1–2 was the absence of a primary source documenting vaccine exposure during pregnancy, such as the antenatal card or a vaccine card.

**Table 4:** Most frequent reasons for not meeting level 3 (among cases not meeting the GAIA definition) and for level 3 cases not meeting levels 1 and 2.

	Not meeting the GAIA definition (at level 3)	Not meeting levels 1 and 2
Low birth weight	NA*	Unknown or inappropriate graduation or calibration of the scale
Preterm birth	NA	No or insufficient information on 1st or 2nd trimester ultrasound
Small for gestational age	The weight was over the 10th percentile for GA on the Intergrowth-21 charts	GA criteria not met (No or insufficient information on 1st or 2nd trimester ultrasound)
Stillbirth	No recorded data on absence or presence of fetal signs of life prior to the onset of labour	GA criteria not met (No or insufficient information on 1st or 2nd trimester ultrasound)
Congenital microcephaly	GA criteria not met (LMP date unknown)	GA criteria not met (No or insufficient information on 1st or 2nd trimester ultrasound)
Neonatal Death	NA*	Birth weight not available and although an estimate of GA was available, GA criteria not met
Neonatal bloodstream infection	Insufficient clinical criteria met	The lack of a sample from a sterile site
Neonatal respiratory infection	Insufficient clinical criteria met	No samples tested; no chest X-ray done
Neonatal meningitis	Fever criterion not met and an insufficient number of other clinical criteria met	Negative (no pathogen identified in the sample) or insufficient findings from a sample tested from CSF or another normally sterile site
Maternal immunization	NA**	No primary source

GA: gestational age; LMP: Last Menstrual Period; NA: not applicable.

*Unresolved queries sent to the site (n = 2 for low birth weight, n = 2 for neonatal death).

**Exposure did not meet the GAIA definition due to a mistake in the design of the case report form (n = 24; 0.6% of exposures assessed).

## Discussion

### Applicability of GAIA definitions

In this study, the applicability of GAIA definitions for seven perinatal and neonatal outcomes and for maternal immunization were assessed using routinely collected data, primarily at tertiary level referral hospitals. The definitions for LBW [19], preterm birth [20], and neonatal death [18] were applicable at the study sites, as nearly all cases recruited by the sites met the GAIA definitions. For neonatal death, nearly all cases met the criteria for level 1 of diagnostic certainty. Birth weight (BW), a key parameter for all the three outcomes, was routinely measured and recorded at all the sites, contributing to the applicability of these case definitions. Kochar et al. also successfully applied the preterm birth case definition using both prospective and retrospective data [30]. A significant proportion of cases of stillbirth [22], SGA [21], congenital microcephaly [24] and neonatal infection [23] (particularly meningitis) did not meet the GAIA definitions, hence these definitions were less applicable at the study sites. This study also gave insight into site-specific levels of diagnostic certainty and pinpointed aspects preventing higher levels of diagnostic certainty from being met, both of which will help inform the design of future vaccine safety studies in pregnancy. The main barrier to obtaining higher levels of diagnostic certainty (for all outcomes except low birth weight, neonatal infection and maternal immunization) was the lack of/ no access to sonographic documentation in first and second trimester and its impact on GA assessment. Better clinical record keeping or documentation of GA in the early stages of pregnancy as part of routine care across caregivers would improve assessment of outcomes requiring GA. In this study, we evaluated how certain we were that a case diagnosed by a site was truly a case; one limitation is that we did not assess how certain we were an outcome was truly absent if not diagnosed. This was not possible as the study was records-based.

The definition for maternal immunization [18] was applicable at the study sites. However, at 5 out of 21 sites, most exposures were classified to level 3, for which no formal documentation of the vaccination is required. The usefulness of level 3 may be limited in vaccine safety surveillance and studies due to the sparsity of information on vaccination. Level 1 was achieved at only two sites, which routinely collected the batch number for vaccines administered as part of antenatal care in their facility. Not all elements of level 1 are necessarily required when studying neonatal outcomes in the context of maternal immunization safety, such as time (hour) of vaccination and batch number. Whereas level 2 does not require the exact date of vaccination (only month and year), however, this information is key to understanding vaccine safety at different stages of fetal development. In the study, due to a mistake in the design of the case report form, 0.6% of identified exposures could not be classified. If the site indicated that the number of doses was not known, the question on data sources was not prompted and therefore not completed, preventing classification to any level. If the number of doses was not known it is likely the source was secondary, which would have enabled classification to Level 3.

Information on maternal immunization was collected from documents available at the sites at the time of case recruitment, frequently the antenatal care card (often allowing to reach level 2 of certainty), or at some sites, the patient record (e.g., whether vaccination was done as per recommendation, often enabling classification only to level 3); no interview with the mother was conducted as part of the study. Furthermore, the antenatal care card belonged to the mother and was normally not available post-discharge. Vaccine exposure status for some mothers was unknown, which might have resulted in missed exposures. Had interviews been conducted, the number of exposures classified to level 3 (instead of unknown) would likely have been higher. More intensive efforts and outreach may be critical for retrieval of information on vaccination status and details of the vaccination. As different COVID19 vaccines are being deployed worldwide and may be used alternatively or simultaneously in each country, the rigorous recording of vaccination exposure in pregnant women is of paramount importance to enable safety monitoring. Advocacy with health program managers and policy makers is needed for improved documentation and inclusion of additional fields vital for maternal immunization pharmacovigilance in the antenatal care card, and increased digitization could enhance reporting and utilizing immunization data in the context of vaccine safety.

We assessed whether the percentage of cases meeting the GAIA definition differed by country, health facility level and public vs private ownership. However, as sites were not selected to be representative of these categories and due to low number of sites in each stratification, we do not feel confident that any differences observed can be meaningfully extrapolated beyond this study.

### Challenges in using the GAIA definitions

Based on our experiences in field-testing the GAIA definitions, we have suggested several improvements to the GAIA definitions that may lead to their easier implementation and increased standardization across studies.

*Accepting higher levels of evidence at lower levels of diagnostic certainty:* In the study, we accepted higher levels of evidence at lower levels of diagnostic certainty to prevent cases from “falling in between” different levels and consequently not being classified. This has been previously documented for the stillbirth definition [25], [30] but we observed the similar issues in the LBW, SGA, neonatal meningitis and maternal immunization definitions (details in S3b).

*Consistency of GA and BW information:* The requirements for GA or BW are not identical across all case definitions. The GA assessment criteria are the same for preterm birth, stillbirth, neonatal death and SGA. However, the GA criteria for congenital microcephaly differ, and are at times more stringent (level 1a necessitates LMP, level 2a does not allow for 1st trimester physical exam, level 3a necessitates LMP and does not allow for other measures such as BW) and sometimes, more lenient (level 1a allows for second trimester ultrasound scan). In addition, for neonatal death level 1, either GA level 1 or BW is necessary for classification, however if GA were assessed using BW it would result in GA level 3.

BW requirements in the LBW case definition were based on the details regarding timing of the measurement and scale specifications. The criteria listed in the SGA case definition differs for SGA level 3, where scale specifications for SGA level 3a are more stringent (resolution of less than 50 g, tared to zero, calibrated) than in the LBW case definition. Furthermore, for the case definitions for neonatal death and preterm birth, no requirements are attached to the BW assessment.

Several other areas of improvement were identified in the maternal immunization (interpretation; see methods section), neonatal death (viability/maturity), stillbirth (GA, signs of life, combination of criteria), congenital microcephaly (chart use) and SGA definitions (S3c).

More countries are adopting policy recommendations for COVID-19 vaccination in pregnant women, based on WHO’s interim recommendations to use COVID-19 vaccines during pregnancy, when the benefits outweigh potential risks. As pregnant women were excluded from clinical trials, safety profile of COVID-19 vaccines will need to be evaluated from post marketing safety data. In this context, it is of critical importance to ensure harmonisation of terminologies, definitions, and methods of assessment to allow comparability and timely assessment through *meta*-analysis of data collected worldwide.

## Conclusion

This prospective study showed that the GAIA definitions for LBW, preterm birth, neonatal death and maternal immunization when vaccination was noted were applicable at the study sites, however, the ones for stillbirth, SGA, neonatal infections and congenital microcephaly were less so. The level of diagnostic certainty of GA was identified as a limiting factor to attaining higher levels of diagnostic certainty for multiple outcomes and several areas of improvement have been suggested following field-testing of the definitions. The introduction of COVID-19 vaccines, and their possible use in pregnant women, reinforces the urgency for improved documentation of vaccination and outcomes vital for maternal immunization pharmacovigilance.

## WHO GVS MCC Sites

Ghana

Joseph HK Donkor, Tema General Hospital, Tema, Ghana.
Richard Wodah-Seme, St. Joseph’s Hospital, Jirapa, Ghana.
Kwasi Baffour Gyimah, Ghana Health Service: Ejisu Government Hospital, Ejisu, Ghana.
Seth Twum, Eastern Regional Hospital, Ghana.

Tanzania

Issa Sabi, National Institute for Medical Research (NIMR), Mbeya Medical Research Center, Mbeya, Tanzania.
Rebecca Mokeha, Mbeya Zonal Referral Hospital, Mbeya, Tanzania.
Elias Kweyamba and Henry Marique, St. Francis Referral Hospital, Ifakara, Tanzania.
Ismail Macha, Mbeya Regional Referral Hospital, Mbeya, Tanzania.

Zimbabwe

Jaensch Masanga Mutede, Mutare Provincial Hospital, Ministry of Health and Child Care, Zimbabwe.
Prosper Chonzi, City of Harare, Health Department, Ministry of Health and Child Care (for Mbare Provincial Hospital), Zimbabwe.

Iran

Maryam Shariati and Elahe Rastkar Mehrabani, Clinical Research Development Center, Mahdiyeh Educational hospital, Shahid Beheshti University of Medical Sciences, Tehran, Iran.

Spain

Alejandro Orrico-Sánchez, Antonio Carmona and Dafina Petrova, Vaccine Research Department, Fundación para el Fomento de la Investigación Sanitaria y Biomédica de la Comunitat Valenciana, FISABIO-Public Health, Valencia, Spain.

India

Javeed Iqbal Bhat, Bashir Ahmad Charoo and Rabia Khurshid Department of Pediatrics, Sher-i-Kashmir Institute of Medical Sciences Srinagar Jammu & Kashmir, India.
Leslie Lewis, Muralidhar Pai, Shyamla G, Jyothi Shetty, Akhila Hebbar, Sripad Hebbar and Prathap Kumar, Kasturba Medical College, Manipal Academy of Higher Education, Manipal, India.
Bhadresh R Vyas, MP Shah Government Medical College, Jamnagar, India.
Lalit Sankhe, Department of Community Medicine, Grant Medical College, Mumbai, India.
Rachita Sarangi and Jagdish Prasad Sahoo, Indian Institute of Medical Sciences and SUM hospital, Bhubaneswar, Orissa, India.
M D Ravi and H V Prajwala, JSS Academy of Higher Education and Research, Mysuru, Karnataka, India.

Nepal

Nisha Keshary Bhatta, Shyam Prasad Kafle, Mukesh Bhatta and Mohan Chandra Regmi, B.P.Koirala Institute of Health Sciences, Dharan, Nepal.
Prerana Kansakar and Ganesh Shah, Department of Pediatrics, Patan Academy of Health Sciences, Nepal.

## CRediT authorship contribution statement

**Anke L. Stuurman:** Conceptualization, Investigation, Methodology, Visualization, Writing – original draft, Writing – review & editing. **Apoorva Sharan:** Conceptualization, Investigation, Methodology, Formal analysis, Writing – review & editing. **Shubhashri Jahagirdar:** Investigation, Data curation, Methodology, Formal analysis, Writing – review & editing. **Varalakshmi Elango:** Investigation, Project administration, Methodology, Writing – review & editing. **Margarita Riera-Montes:** Conceptualization, Investigation, Supervision, Resources, Methodology, Writing – review & editing. **Neeraj Kashyap:** Software, Data curation, Writing – review & editing. **Jorne Biccler:** Data curation, Formal analysis, Visualization, Writing – review & editing. **Ramesh Poluru:** Formal analysis, Data curation, Writing – review & editing. **Narendra Arora:** Conceptualization, Methodology, Resources, Writing – review & editing. **Matthews Mathai:** Conceptualization, Methodology, Writing – review & editing. **Punam Mangtani:** Conceptualization, Methodology, Writing – review & editing. **Hugo DeVlieger:** Conceptualization, Methodology, Writing – review & editing. **Steven Anderson:** Conceptualization, Methodology, Writing – review & editing. **Barbee Whitaker:** Conceptualization, Methodology, Writing – review & editing. **Hui-Lee Wong:** Methodology, Writing – review & editing. **Clare Cutland:** Conceptualization, Methodology, Writing – review & editing. **WHO GVS MCC Sites:** Conceptualization, Investigation, Writing- review and editing. **Christine Guillard Maure:** Conceptualization, Funding acquisition, Project administration, Supervision, Writing- review and editing

## Declaration of Competing Interest

The authors declare the following financial interests/personal relationships which may be considered as potential competing interests: Christine Guillard Maure reports financial support was provided by Bill & Melinda Gates Foundation.

## Appendix A Supplementary material

Supplementary data S1 Protocol (Original and Amended)

Supplementary data S1

Supplementary data S2 Data sources

Supplementary data S3 Using GAIA definitions

Supplementary data S4 Site-Specific Results (tables and figures)

## References

[1] Swamy G.K., Beigi R.H. (2015). Maternal benefits of immunization during pregnancy. Vaccine.

[2] Omer S.B., Goodman D., Steinhoff M.C., Rochat R., Klugman K.P., Stoll B.J. (2011). Maternal Influenza Immunization and Reduced Likelihood of Prematurity and Small for Gestational Age Births: A Retrospective Cohort Study. PLoS Med.

[3] World Health Organization (WHO), Maternal and Neonatal Tetanus Elimination - Progress towards global MNT elimination, 2020, https://www.who.int/immunization/diseases/MNTE_initiative/en/

[4] Englund J.A. (2015). Maternal immunization–Promises and concerns. Vaccine.

[5] Amirthalingam G., Andrews N., Campbell H., Ribeiro S., Kara E., Donegan K. (2014). Effectiveness of maternal pertussis vaccination in England: an observational study. Lancet.

[6] Madhi S.A., Cutland C.L., Kuwanda L., Weinberg A., Hugo A., Jones S. (2014). Influenza vaccination of pregnant women and protection of their infants. N Engl J Med.

[7] Turner H.C., Thwaites G.E., Clapham H.E. (2018). Vaccine-preventable diseases in lower-middle-income countries. Lancet Infect Dis.

[8] Keller-Stanislawski B., Englund J.A., Kang G., Mangtani P., Neuzil K., Nohynek H. (2014). Safety of immunization during pregnancy: a review of the evidence of selected inactivated and live attenuated vaccines. Vaccine.

[9] Centers for Disease Control and Prevention (CDC), Guidelines for Vaccinating Pregnant Women, 2016. https://www.cdc.gov/vaccines/pregnancy/hcp-toolkit/guidelines.html. (Accessed January 21 2021).

[10] Laris-Gonzalez A., Bernal-Serrano D., Jarde A., Kampmann B. (2020). Safety of Administering Live Vaccines During Pregnancy: A Systematic Review and Meta-Analysis of Pregnancy Outcomes. Vaccines (Basel).

[11] Lackritz E, Stergachis A, Stepanchak M, Englund J, Tavares Da Silva F, Sevene E, et al., Maternal Immunization Safety Monitoring in Low- and Middle-Income Countries: A Roadmap for Program Development. Building an approach that is practical, affordable, and sustainable, In: Eve M. Lackritz, Andy Stergachis, M. Stepanchak (Eds.) Global Alliance to Prevent Prematurity and Stillbirth, 2017.

[12] World Health Organization (WHO). Global vaccine safety blueprint, Department of Immunization, Vaccines and Biologicals, Geneva; 2012. https://apps.who.int/iris/handle/10665/70919.

[13] Maure C.G., Dodoo A.N., Bonhoeffer J., Zuber P.L. (2014). The Global Vaccine Safety Initiative: enhancing vaccine pharmacovigilance capacity at country level. Bull World Health Organ.

[14] Guillard-Maure C, Elango V, Black S, Perez-Vilar S, Castro JL, Bravo-Alcantara P, et al. Operational lessons learned in conducting a multi-country collaboration for vaccine safety signal verification and hypothesis testing: The global vaccine safety multi country collaboration initiative, Vaccine 36(3) (2018) 355-362.DOI: 10.1016/j.vaccine.2017.07.085. 10.1016/j.vaccine.2017.07.085 28780118

[15] Bonhoeffer J, Kochhar S, Hirschfeld S, Heath PT, Jones CE, Bauwens J, et al.,Global alignment of immunization safety assessment in pregnancy - The GAIA project, Vaccine 34(49) (2016) 5993-5997.DOI: 10.1016/j.vaccine.2016.07.006. 10.1016/j.vaccine.2016.07.006 27751641

[16] Bonhoeffer J., Kohl K., Chen R., Duclos P., Heijbel H., Heininger U. (2002). The Brighton Collaboration: addressing the need for standardized case definitions of adverse events following immunization (AEFI). Vaccine.

[17] Brighton Collaboration, Brighton Collaboration definitions, https://brightoncollaboration.us/about/. (Accessed 22 January 2021).

[18] Pathirana J., Muñoz F.M., Abbing-Karahagopian V., Bhat N., Harris T., Kapoor A. (2016). Neonatal death: Case definition & guidelines for data collection, analysis, and presentation of immunization safety data. Vaccine.

[19] Cutland CL, Lackritz EM, Mallett-Moore T, Bardaji A, Chandrasekaran R, Lahariya C, et al. Low birth weight: Case definition & guidelines for data collection, analysis, and presentation of maternal immunization safety data, Vaccine 35(48 Pt A) (2017) 6492-6500.DOI: 10.1016/j.vaccine.2017.01.049. 10.1016/j.vaccine.2017.01.049 PMC5710991 29150054

[20] Quinn JA, Munoz FM, Gonik B, Frau L, Cutland C, Mallett-Moore T, et al., Preterm birth: Case definition & guidelines for data collection, analysis, and presentation of immunisation safety data, Vaccine 34(49) (2016) 6047-6056.DOI: 10.1016/j.vaccine.2016.03.045. 10.1016/j.vaccine.2016.03.045 PMC5139808 27743648

[21] Schlaudecker EP, Munoz FM, Bardaji A, Boghossian NS, Khalil A, Mousa H, et al.,Small for gestational age: Case definition & guidelines for data collection, analysis, and presentation of maternal immunisation safety data, Vaccine 35(48 Pt A) (2017) 6518-6528.DOI: 10.1016/j.vaccine.2017.01.040. 10.1016/j.vaccine.2017.01.040 PMC5710996 29150057

[22] Tavares Da Silva F., Gonik B., McMillan M., Keech C., Dellicour S., Bhange S. (2016). Stillbirth: Case definition and guidelines for data collection, analysis, and presentation of maternal immunization safety data. Vaccine.

[23] Vergnano S, Buttery J, Cailes B, Chandrasekaran R, Chiappini E, Clark E, et al.,Neonatal infections: Case definition and guidelines for data collection, analysis, and presentation of immunisation safety data, Vaccine 34(49) (2016) 6038-6046.DOI: 10.1016/j.vaccine.2016.03.046. 10.1016/j.vaccine.2016.03.046 PMC5139809 27491687

[24] DeSilva M., Muñoz F., Sell E., Marshall H., Tse Kawai A., Kachikis A. (2017). Congenital microcephaly: Case definition & guidelines for data collection, analysis, and presentation of safety data after maternal immunisation. Vaccine.

[25] Stuurman AL, Riera M, Lamprianou S, Perez-Vilar S, Anderson SA, Mangtani P, et al.,Vaccine safety surveillance in pregnancy in low- and middle-income countries using GAIA case definitions: A feasibility assessment, Vaccine 36(45) (2018) 6736-6743.DOI: 10.1016/j.vaccine.2018.09.033. 10.1016/j.vaccine.2018.09.033 30266486

[26] Stuurman AL, Elango V, Riera M, Biccler J, Nagarajan A, Sharan A, et al. Global Vaccine Safety Multi Country collaboration project measuring risks of early childhood morbid conditions and assessing standardized methods, 2021. https://apps.p-95.com/WHO/.

[27] INCLEN, Somaarth-III, a tool for cross-sectional studies, http://inclentrust.org/inclen/somaarth-3/. (Accessed February 25 2021).

[28] R Core Team, R: A Language and Environment for Statistical Computing, R Foundation for Statistical Computing, Vienna, Austria; 2019.

[29] StataCorp., Stata Statistical Software: Release 15, StataCorp LLC, College Station, TX; 2017.

[30] Kochhar S, Clarke E, Izu A, Emmanuel Kekane – Mochwari K, Cutland CL. Immunization in pregnancy safety surveillance in low and middle-income countries- field performance and validation of novel case definitions. Vaccine 37(22) (2019) 2967-2974.DOI: https://doi.org/10.1016/j.vaccine.2019.03.074. 10.1016/j.vaccine.2019.03.074 31014963

