# Supplementary material for: WHO global vaccine safety multi-country collaboration project on safety in pregnancy: Assessing the level of diagnostic certainty using standardized case definitions for perinatal and neonatal outcomes and maternal immunization
Source: Vaccine X. 2021 Nov 3;9:100123. doi: 10.1016/j.jvacx.2021.100123 (PMC8605263; doi:10.1016/j.jvacx.2021.100123)
Supplement: Supplementary data S1 — Protocol (Original and Amended) [file mmc1.pdf]

Protocol

**Short title**	
**Title**	Global Vaccine Safety Multi Country collaboration project measuring risks of early childhood morbid conditions and assessing standardized methods
**Study ID**	
**Sponsor**	WHO
**Date of version**	November 1, 2019
**Version**	3.0 (amendment 2)

Version	Version Date	Reason of change
1.0	15Oct18	
2.0	30Nov18	Clarify the timelines for data collection.<br>Change in information recorded on deliveries.<br>Update the CRFs accordingly
2.1	26 June 2019	Correction of one of the two satellite sites in Spain
3.0	1 Nov2019	Collection of 4 additional data points to obtain reliable count of outcomes of interest<br>Simplify the recruitment rules of study participants<br>Update information on participating sites, scientific committee members and timelines

Table of Contents

1 Study teams 5
2 Background 9
2.1 Rationale 10
3 Objectives 12
3.1 Primary objectives 12
3.2 Secondary objectives 12
3.3 Exploratory objectives 12
4 Methodology 13
4.1 Study design 13
4.2 Study setting 13
4.3 Study population 14
4.4 Study period 15
4.5 GAIA definitions 15
4.6 Registration of all deliveries 15
4.7 Case identification and vaccine exposure assessment 15
4.7.1 Identification of congenital microcephaly 16
4.7.2 Identification of low birth weight 16
4.7.3 Identification of neonatal death 16
4.7.4 Identification of neonatal infection 16
4.7.5 Identification of preterm birth 16
4.7.6 Identification of small for gestational age 16
4.7.7 Identification of stillbirth 16
4.7.8 Ascertainment of vaccine exposure 16
4.8 Data sources 17
4.9 Data collection 18
4.10 Case classification 19
5 Statistical methods 20
5.1 Sample size 20
5.1.1 Primary study outcome 1: Minimum detectable risk 20
5.1.2 Primary study outcome 2: Applicability of GAIA case definitions 20
5.2 Data analysis: Primary study outcomes 21
5.2.1 Primary study outcome 1 – Minimum detectable risk 21
5.2.2 Primary study outcome 2: Applicability of GAIA case definition 22

5.3 Secondary study outcomes 22
5.3.1 Secondary study outcome 1 – Proportion of cases classified by level 22
5.3.2 Secondary study outcome 2 – Factors influencing applicability 23
5.4 Exploratory study outcomes 24
5.4.1 Missing elements to meet GAIA case definition 24
5.4.2 Missing elements to reach a better level of diagnostic certainty 24
5.5 Handling of missing data 24
6 Limitations 25
7 Data reporting 26
7.1 Study Report 26
7.2 Dissemination strategy 26
8 Study management 27
8.1 Data management 27
8.2 Data entry/ EDC 27
8.3 Source documents 27
8.4 Data security 28
8.5 Data transfer procedure 28
8.6 File retention and archiving 29
8.7 Quality assurance and monitoring 29
8.8 Changes to the protocol 29
9 Ethical and Regulatory Considerations 31
9.1 Respecting participant autonomy 31
9.2 Study participant confidentiality 32
9.3 Independent Ethics Committee/ Institutional Review Board 32
10 Expected Timelines 33
11 References 34
Annex I. Site Selection 36
Annex II. Site descriptions 37
Annex III. GAIA case definitions 47
Annex IV. ICD codes 64
Annex V. CRFs 72
Annex VI. Patient information sheet 95

Acronyms

AEFI	Adverse Event Following Immunization
CI	Confidence interval
CRF	Case report form
CIOMS	Council for International Organizations of Medical Sciences
DMP	Data management plan
eCRF	Electronic case report form
EDC	Electronic data capture
GAIA	Global Alignment of Immunization Safety Assessment in Pregnancy
GACVS	Global Advisory Committee on Vaccine safety
GDPR	General Data Protection Regulation
HICs	High income countries
ICD	International Classification of Diseases
LMICs	Low and middle-income countries
LMP	Last menstrual period
PPV	Positive predictive value
RSV	Respiratory Syncytial Virus
USA	United States of America
WHO	World Health Organization

1 Study teams

Study teams

Organization	Name	Capacity
**Research team**		
WHO	Christine Maure	
WHO	Patrick Zuber	
P95	Varalakshmi Elango	Project manager
P95	Anke Stuurman	Epidemiologist
P95	Margarita Riera	Epidemiologist
P95	Kaatje Bollaerts	Statistician
P95	Maria Alexandridou	Data analyst
P95	Jorne Biccler	Data analyst
P95	Thomas Verstraeten	Epidemiologist
INCLEN	Apoorva Sharan	Project officer
INCLEN	Neeraj Kashyap	Project officer IT
INCLEN	Shubhashri Jahagirdar	Project officer
INCLEN	Jayant Kumar	IT research assistant
INCLEN	Ramesh Poluru	Statistician
INCLEN	Vaibhav Miglani	Statistician
INCLEN	tbd	Data manager/IT
INCLEN	Narendra Arora	Project coordinator
**Sites**		
Ghana, St. Joseph's Hospital	Dr. Richard Wodah-Seme	Principal Investigator
Ghana, Ejisu Government Hospital	Dr. Kwasi Baffour Gyimah	Principal Investigator
Ghana, Tema General Hospital	Dr Joseph Horatius Kojo Donkor	Principal Investigator
Ghana, Eastern Regional Hospital	Dr Seth Twum	Principal Investigator
India, JSS Hospital, Department of Pediatrics	Dr Mandyam Ravi	Principal Investigator
India, Grants Government Medical College and Sir JJ Group of Hospitals	Pr Lalit Sankhe	Principal Investigator
India, Institute of Medical Sciences (IMS) and Sum Hospital	Dr Rachita Sarangi	Principal Investigator
India, Kasturba Medical College, Department of Pediatrics	Pr Leslie Lewis	Principal Investigator
India, MP Shah Government Medical College	Pr Bhadresh Vyas	Principal Investigator

India, Sher-i-Kashmir Institute of Medical Sciences (SKIMS)	Dr Javeed Iqbal Bhat	Principal Investigator
Iran, Mahdieh Hospital	Dr Maryam Khoshnoud Shariati	Principal Investigator
Iran, Shohada Teaching Hospital	Dr Mahta Basir	Principal Investigator
Iran, Akbar Abadi Maternity and Neonatal Hospital	Dr Maryam Rahimi	Principal Investigator
Nepal, Patan Academy of Health Sciences-PAHS (Patan Hospital), Department of Paediatrics	Prof. Imran Ansari	Principal Investigator
Nepal, B.P. Koirala Institute of Health Sciences, Head, Department of Paediatrics	Prof. Dr. Nisha Keshari Bhatta	Principal Investigator
Spain, FISABIO, Fundación para el Fomento de la Investigación Sanitaria y Biomédica de la Comunitat Valenciana, Hospital General Universitario de Castellón	Dr Javier Diez<br>Dr Alejandro Orrico-Sanchez	Principal Investigator and Co- Principal Investigator
Spain, FISABIO, Fundación para el Fomento de la Investigación Sanitaria y Biomédica de la Comunitat Valenciana, Hospital Universitario Doctor Peset, Valencia.	Dr Javier Diez<br>Dr Alejandro Orrico-Sanchez	Principal Investigator and Co- Principal Investigator
Tanzania, St.Francis Referral hospital	Dr Elias Kweyamba	Principal Investigator
Tanzania, National Institute for Medical Research - Mbeya Medical Research Center, Mbeya Regional Referral Hospital (MRRH)	Dr Issa Sabi (NIMR-MMRC)<br>Dr Ismail Macha	Principal Investigator and Co- Principal Investigator
Tanzania, National Institute for Medical Research - Mbeya Medical Research Center, Mbeya Zonal Referral Hospital (MZRH)	Dr Issa Sabi (NIMR-MMRC)<br>Dr. Rebecca Mokeha	Principal Investigator and Co- Principal Investigator
Zimbabwe, Mutare Provincial Hospital	Dr Jaensch Dorcas Masanga-Mutede	Principal Investigator

Zimbabwe, Mbare Polyclinic, (Edith Opperman Maternity Hospital)	Ms Phillomina Chitando	Principal Investigator
**Scientific committee**		
LSHTM	Punam Mangtani	Epidemiologist
Universitair Ziekenhuis Leuven	Hugo DeVlieger	Neonatologist
US FDA	Steven Anderson	Biostatistician and epidemiologist
PAHS, Kathmandu, Nepal	Neelam Adhikari	Paediatrician
US FDA	Barbee Whitaker	Infectious disease expert and epidemiologist
LSTM	Matthews Mathai	Obstetrician
African Leadership in Vaccinology Expertise (Alive), University of the Witwatersrand	Clare Cutland	Clinical researcher and Scientific coordinator

Roles and responsibilities

Scientific committee
Provide scientific guidance in protocol development and implementation
Review study data analysis and study results
WHO (Headquarters)
Oversight and coordination
Site selection
Ethics and other approval at WHO level
Country coordinators/managers
Primary contact point between sites in a country and WHO
P95/INCLEN
Development of EDC system and pilot testing
Country-level training
Data management plan and statistical analysis plan
Coordinates receipt of data from all sites
For-cause site monitoring
Data cleaning, including sending site-specific queries
Data analysis
Reporting

Study Sites
Ethics and other approval at local level
Pilot testing of EDC at selected sites
Obtaining informed consent
Case identification
Completion of eCRFs
Answering site-specific queries

2 Background

Immunological and physiological changes during pregnancy may increase a woman's risk of infection. The relative immunological inexperience of the foetus and neonate poses additional risk of infection for the developing infants as well [1]. Through 2015, neonatal death accounted for approximately 46% of mortality among children less than 5 years of age globally and of these deaths, 25% were caused by infection [2].

Immunization during pregnancy can protect the pregnant woman and her child, both in the womb and in early life. The goal of immunizing women during pregnancy is to increase their antibody titres against certain vaccine-preventable diseases, so that high and protective levels of antibody are transferred to the infant during the pregnancy. Hence it can prevent infectious diseases in infants who are too young to benefit directly from primary immunization and protect the foetus from the effects of maternal infection during development.

Many diseases have a disproportionate impact on the young infant. These include pertussis, influenza, group B streptococcal (GBS) infection, respiratory syncytial virus (RSV) and tetanus. For tetanus, pertussis and influenza, immunization in pregnancy is a key strategy to prevent significant morbidity and mortality amongst young infants globally and it also holds great promise as a strategy to protect infants from other infections [3-5]. The ultimate goal of immunization in pregnancy is to decrease morbidity and mortality among pregnant women, neonates and infants. This public health strategy is of specific interest for low and middle-income countries (LMICs), where access to basic health services may be limited and the burden of vaccine-preventable diseases is largest. Promising new vaccine products are being developed for use during pregnancy to protect unborn children and neonates from infection, such as vaccines against Zika, RSV, group B streptococci and hepatitis E vaccines. [6]

The risk-benefit ratio of vaccines in pregnancy must be assessed by balancing vaccine safety with the risk of infection for the pregnant woman and her foetus or infant in the absence of immunization [1]. Vaccine safety can only be inferred from low rates of adverse events following immunization (AEFI) that have been assessed in a relatively large population, and cannot be evaluated directly [7]. Vaccine safety surveillance is complex, and even more so during pregnancy, where the risk of adverse outcomes may change as a result of exposure to infection or vaccination over the gestational period [1]. In LMICs, safety monitoring is further challenged by the general lack of pharmacovigilance infrastructure [8].

The World Health Organization (WHO) Global Advisory Committee on Vaccine safety (GACVS) has evaluated the data on the safety of immunization of pregnant women for several inactivated and live attenuated vaccines. It concluded that there is no evidence of adverse pregnancy outcomes from the vaccination of pregnant women with inactivated virus, bacterial vaccine, or toxoid [1].

Post-marketing safety surveillance of new vaccines used during pregnancy is important for the detection of any AEFI in pregnant women and their infants for a number of reasons. First, background rates for the various outcomes of interest are not available in most LMICs; second, large populations are required to identify potential rare AEFI [7]. In addition, post-marketing safety surveillance may help to overcome existing barriers to vaccination during pregnancy as they will inform on the supposed risks to the foetus [1].

In response to the call of WHO for a globally concerted approach to monitor the safety of vaccine in pregnancy, the Global Alignment of Immunization Safety Assessment in Pregnancy (GAIA) project was launched in 2015. The aim of GAIA, managed by the Brighton Collaboration [9], was to improve generated data to facilitate data comparability and interpretation across surveillance systems, ultimately leading to strengthened programs of immunization in pregnancy [10, 11]. To this end, standardized definitions for obstetric and neonatal health outcomes were developed. The purpose of this harmonization and standardization is to increase the ability to conduct meaningful comparisons of safety data across studies [7, 9]. Within each case definition, multiple 'Levels of diagnostic certainty' are recognized, so that the definitions are globally applicable for all immunization safety purposes and in settings with different levels of diagnostic capacities [9].

As several promising vaccines in development would be of great benefit specifically for women and children in low and middle-income countries (LMICs), due to higher perinatal and infant mortality rates, it is now critical that those countries develop vaccine safety monitoring beyond passive surveillance, in this specific population.

In an effort to address WHO's Global Vaccine Safety Blueprint (5) strategic goal of enhanced pharmacovigilance capacity, the Global Vaccine Safety Initiative (6), through its WHO secretariat, tested the development of a global network of hospital-based sentinel sites of LMICs across the WHO regions for vaccine safety signal verification and hypothesis testing. The aim of the project was to identify optimal models and processes for establishment and operationalization of such a network. The lessons learnt from this proof of concept project will be integrated in the development of this study [12].

2.1 Rationale

The goal of this prospective study is to estimate the minimum detectable risk for selected neonatal outcomes (neonatal death, neonatal infection, congenital microcephaly, low birth weight, preterm birth, small for gestational age, and stillbirth) in each study site using standardised case definitions and to assess the applicability of GAIA case definitions in LMICs, in order to inform future vaccine safety studies.
The use of GAIA case definitions can be challenging in LMICs, as the proposed criteria for some definitions can exceed the clinical capacity of some sites. The study will assess the applicability of GAIA definition for health outcomes. The health outcomes were selected based on relevance and perceived complexity of data collection. For future vaccine safety

study, sites should be able to link vaccination status information of pregnant women to obstetric and neonatal outcomes. Hence the project will also assess the current sites' capacity for data identification and collection on maternal immunisation status.

3 Objectives

The goals of this study are to estimate the minimum detectable risk of selected neonatal outcomes and to assess the applicability of GAIA case definitions in LMICs.

The GAIA definitions of interest for this study are: congenital microcephaly, low birth weight, neonatal death, neonatal infection, preterm birth, small for gestational age, stillbirth, and maternal immunisation.

3.1 Primary objectives

1. To calculate the minimum detectable risk for each health outcome
2. To assess the applicability of the GAIA definitions for the health outcomes of interest and maternal immunisation.

3.2 Secondary objectives

1. To assess the proportion of cases or exposures identified than can be classified according to each GAIA definition *level* of diagnostic certainty.
2. To identify factors that influence the applicability of the GAIA definitions.

3.3 Exploratory objectives

1. To assess which missing data elements are preventing identified cases from meeting the GAIA case definition lowest level of diagnostic certainty.
2. To assess which missing data elements are preventing classified cases from meeting a better level of diagnostic certainty.

4 Methodology

4.1 Study design

This will be a prospective observational study at selected sentinel sites, mostly in LMICs.

As this study is observational, no changes to routine clinical and diagnostic practice will take place, however data recording from routine clinical practice is expected to be more complete and data access easier than in a retrospective study. During a feasibility assessment it was found that, at the majority of sites, medical records are not available in electronic format and archives are not easily accessible. Sites also expressed concerns on the limitation of their medical records maintenance: data that had been generated might not be recorded or might not be retrieved. Hence a prospective study design is proposed to ease access to quality data.

4.2 Study setting

The study will be performed at 25 sites in seven LMICs (Ghana, South Africa, Tanzania, Zimbabwe, Iran, India, Nepal) and one high-income country (HIC) (Spain). The sites consist of one primary care centre, four secondary and eighteen tertiary hospitals with a maternity ward. Sites have been selected based on site selection criteria and acceptable performance in a simulation exercise that tested sites' actual minimum capacity in data collection (Annex 1).

The following sites will participate (country, city, site). Sites characteristics are further described in Annex 2:

African Region

- Ghana, Jirapa, St Joseph's Hospital
- Ghana, Ejisu-Juaben, Ejisu Government Hospital
- Ghana, Tema, Tema General Hospital
- Ghana, Koforidua, Eastern Regional Hospital city,
- Tanzania, Mbeya, Mbeya Regional Referral hospital
- Tanzania, Mbeya, Mbeya Zonal Referral hospital
- Tanzania, Ifakara Morogoro, St Francis Referral Hospital
- Zimbabwe, Harare, Mbare Policlinic
- Zimbabwe, Mutare, Mutare Provincial Hospital

Eastern Mediterranean Region

- Iran, Teheran, Mahdieh
- Iran, Teheran, Shohada
- Iran, Teheran, Akbarabadi

European Region

- Spain, Valencia, Fundación para el Fomento de la Investigación Sanitaria y Biomédica de la Comunitat Valenciana, Hospital General Universitario de Castellón
- Spain, Valencia, Fundación para el Fomento de la Investigación Sanitaria y Biomédica de la Comunitat Valenciana, Hospital Universitario Doctor Peset, Valencia.
-

South-East Asia Region

- India, Mysore, JSS Hospital
- India, Mumbai, Grants Government Medical College and Sir JJ Group of Hospitals (GMC)
- India, Bhubaneswar, Institute of Medical Sciences (IMS) and Sum Hospital
- India, Manipal, Kasturba Medical College
- India, Jamnagar, MP Shah
- India, Soura, Sher-i-Kashmir Institute of Medical Sciences (Skims) Soura
- Nepal, Kathmandu, Patan Hospital
- Nepal, Dharan, B.P. Koirala Institute of Health Sciences

4.3 Study population

The study population will consist of all infants delivered (including stillborn) at the site during a one-year period after study start and their mother.

Mothers of all infants (including prenatally and stillborn) with a diagnosis of at least one condition of interest during the study period delivered at the site may be invited to participate in the study.

Since some sites may exceed the required number of infants with the outcome (as per section 5.1), 100 cases for each outcome will be consecutively recruited.

Inclusion criteria

The inclusion criteria for infants (alive or stillborn) are listed below. Mothers of eligible infants will also be enrolled.

- Delivered at the site at which the study takes place
- With a diagnosis of one of the health outcomes of interest at the same site during the study period
- Informed consent from mother

Exclusion criteria

- Infants not delivered at the site; including children diagnosed with one of the outcomes of interest at the site during the study period but not delivered at the site
- Spontaneous abortions (i.e. pregnancy loss prior to the prespecified gestational age that defines a stillbirth)
- No informed consent given

4.4 Study period

Infants (including stillborn) delivered over a one-year period (tentatively between February 1, 2019 and January 31st, 2020) with one of the outcomes of interest will be eligible for inclusion. Data collection on identified cases will continue for an additional 28 days, covering the neonatal period for the last delivery registered.

4.5 GAIA definitions

GAIA case definitions for the following health outcomes will be used (Annex 3):

- Congenital microcephaly [13]
- Low birth weight [14]
- Neonatal death [15]
- Neonatal infection [16]
- Preterm birth [15]
- Small for gestational age [17]
- Stillbirth [18]

The GAIA definition for maternal immunization [15] will be used to assess exposure to maternal tetanus vaccination or any other vaccine provided during pregnancy.

4.6 Registration of all deliveries

All deliveries that take place at the site within one year of study start will be registered. This will include the date of delivery, the delivery outcome (live birth, foetal death), the sex, birth weight, gestational age, head circumference and the presence of any of the conditions of interest within 28 days of birth.

4.7 Case identification and vaccine exposure assessment

Identified cases are defined as children delivered at the site with a diagnosis of one of the health outcomes of interest at the same site. A list of potential cases will be drawn on a regular basis (e.g. weekly) from relevant data sources (see section 3.7).

4.7.1 Identification of congenital microcephaly

Congenital microcephaly is defined by the measurement of occipital-frontal circumference (head circumference) that is more than 2 standard deviations below the mean for age and sex or less than the 3rd percentile for age and sex [13]. Congenital microcephaly can be diagnosed *prenatally* (≥24 weeks of gestational age) or *postnatally* (measurements up to 6 weeks after birth). For this study, we will only consider postnatally diagnosed microcephaly.

4.7.2 Identification of low birth weight

Low birth weight is defined as first weight recorded within hours of birth at <2500 grams. It comprises *very low birth weight* (< 1500 grams) and *extremely low birth* weight (<1000 grams) [15].

4.7.3 Identification of neonatal death

Neonatal death is defined as death of a live born child within 28 days of birth. It comprises neonatal death in *non-viable live births*, *extremely preterm live births*, *preterm live births* (≥28 weeks to <37 weeks), and in *term live births* [18].

Gestational ages cut-offs will be used regarding what is considered a non-viable live birth at each site/country.

4.7.4 Identification of neonatal infection

Neonatal infection is defined as an *invasive bloodstream infection*, *respiratory infection* or *meningitis* within 28 days of birth [14].

4.7.5 Identification of preterm birth

Preterm birth is defined as gestational age at birth of <37 weeks [19].

4.7.6 Identification of small for gestational age

Small for gestational age is defined as birth weight below 10th percentile for gestational age [16].

4.7.7 Identification of stillbirth

Stillbirth is defined as foetal death occurring before birth after a selected, predefined duration of gestation. It comprises intrapartum stillbirth and antepartum stillbirth [17].

4.7.8 Ascertainment of vaccine exposure

To assess whether sites are able to determine whether a vaccine had been administered during pregnancy or within 30 days prior to the last menstrual period (LMP). Maternal vaccination status for tetanus or any other vaccines administered during pregnancy will be ascertained for all mothers of identified cases with the health outcome of interest.

A mother who has any mention of maternal vaccination will be considered vaccinated (exposed). This could be a formal recording of vaccination or any other mention in the documents available to the sites.

A mother who has documented evidence of absence of maternal vaccination will be considered non-vaccinated (non-exposed).

Any mother without any indication will be considered to have exposure status 'unknown'.

4.8 Data sources

Number of live births

The number of live births at each site, for a period of one year will be ascertained using data from maternity wards.

Case identification

Cases will be identified based on manual review of registries and medical records from maternity and pediatric wards and neonatal intensive care units, through free text search or using ICD9 or ICD10 codes (Annex 4). Registries of interest include, but are not limited to, delivery, admission and discharge registries. Data sources vary between health outcomes and between sites, subject to relevance and availability. All relevant data sources to which a site has access may be used. Data collected during this prospective study must be based on data recorded on the appropriate source documents.

CRF completion

Individual patient records from the mother and the child (including medical records, antenatal care records, antenatal care card, vaccination records), will be used to complete the CRFs.

Factors that potentially affect classification

Information on factors that potentially affect classification were collected through a one-off questionnaire that will be completed once by each site. This will include questions on:

- Health facility level (primary, secondary, tertiary/referral)
- Health facility type (public, private)
- Qualification of staff to identify cases and to complete CRFs
- Availability of technical equipment (e.g. laboratory, ultrasound, type of scale etc.)
- Record keeping.

In addition, the questionnaire will contain a question on the gestational age cut-off that is considered non-viable live birth at each site/country (see section 4.6.3).

4.9 Data collection

Each delivery that takes place at the site will be registered and the occurrence of any outcomes of interest within 28 days of birth will be recorded in the electronic data capture system (EDC). In addition, the sex, birth weight, gestational age and the head circumference will be collected. From this, the total number of live births over one year and the total number of cases identified among those delivered at the site will be derived.

Once an outcome occurs and the inclusion and exclusion criteria are met (including informed consent), the infant enters the study. At each site, cases with outcomes of interest will be consecutively recruited into the study, until 100 cases for each outcome have been recruited, or the end of the study period is reached, whichever occurs first.

One infant may present with more than one outcome under study; cases will be recruited for all outcomes until 100 cases for each outcome has been reached. . Data on case ascertainment for each case and maternal vaccination status of the case's mother will be collected in an electronic CRF in an electronic data capture (EDC) system. CRFs are available in Annex 5. If necessary, a paper CRF may be used as an intermediary step.

The EDC system will generate a unique ID for each child. Forms for child and mother shall be automatically linked to this unique ID where appropriate. Country and site codes shall also be assigned by the data management centre.

Investigators at the sites need to be able to link the mother-child pair enrolled in the study with their respective medical records (including vaccination records for the mother). To this end, a password-protected Excel/CSV file containing the mother-child pair's study number and personal data required for unequivocal patient identification (name and surname, gender, date of birth, unique personal identification number if available) will be created and kept locally. No additional data will be included in this file. This file will never leave the site or be shared with anybody not involved in the investigation. This file will be stored in the tablet in which the EDC application will be installed. The EDC will be able to read the file when required during data collection at the site. Access to the files stored in the tablet will be restricted to authorised staff at the site only, and the EDC will not upload the file on the centralized study server. Backup of the file and source documents will be maintained at study sites on periodic basis.

Harmonized data collection procedures will be described in a specific manual of study procedures.

4.10 Case classification

Identified cases and exposures will be classified using the Brighton Collaboration ABC tool (Automatic Brighton Classification) [20] according to the levels of diagnostic certainty present in the GAIA definitions (Level 1-3, and Level 4-5 when applicable).

5 Statistical methods

Data will be analysed by objective and by health outcome, using appropriate statistical software (e.g. SAS, R, SPSS).

5.1 Sample size

5.1.1 Primary study outcome 1: Minimum detectable risk

No sample size calculation is required for this outcome. All cases identified during the study period will be included in the study.

5.1.2 Primary study outcome 2: Applicability of GAIA case definitions

Sample size estimations for these outcomes are provided as an indication of the precision level that can be achieved, depending on the number of cases identified per health outcome per site during the study period.

Table 1 shows the number of cases required for different levels of precision and expected proportions of applicability.

Table 1. Number of cases required for different levels of precision

	**Expected proportions (applicability)**		
**Relative precision**	**20%**	**50%**	**80%**
**10% (+/- 5%)**	246	384	246
**20% (+/-10%)**	62	97	62
**30% (+/- 15%)**	28	43	28
**40% (+/- 20%)**	16	25	16

Assuming proportions of 50% ('worst case'), 97 cases would enable the calculation of proportions with 20% relative precision.

In the analysis, proportions for the primary analysis will be calculated overall and stratified by site, country, region, health facility level of care. The smallest unit is the site. Therefore, 97 cases per outcome per site would be required for 20% precision around proportions of 50%.

For this reason, we aim to enroll 100 cases per outcome per site. Failure to achieve the target of 100 for some outcomes and sites due to a low occurrence of the outcome under study will result in a loss of precision.

For some sites and outcomes, the number of subjects identified with the outcome of interest may exceed 100 during the study period. At each site, cases with outcomes of interest will be consecutively recruited into the study, until 100 cases for each outcome have been recruited, or the end of the study period is reached, whichever occurs first.

5.2 Data analysis: Primary study outcomes

5.2.1 Primary study outcome 1 – Minimum detectable risk

The minimum detectable risk that can be detected for each health outcome in a future study will be quantified for each GAIA case definition. The minimum detectable risk will be the minimum detectable odds ratio (OR) in a case-control study design and the minimum detectable relative risk (RR) in a cohort study design. This information will be useful to establish the sample size that can be achieved in a one-year period for a specific health outcome in future studies, and the minimum detectable risk that could be achieved. Note that the data collected on maternal vaccination during the study will not be used in this calculation, as this data were collected solely to assess the applicability, and not to assess risk associated with maternal vaccinations currently in use.

For the case-control study design, the minimum detectable OR for each health outcome will be calculated based on:

- Number of cases classified during the study period,
- Different theoretical levels of exposure (maternal vaccination) among cases and controls,
- The ratio of cases to controls,
- 80% power and 95% confidence level,
- Per year of study duration.

For the cohort study design, the minimum detectable RR for each health outcome will be calculated based on:

- Different theoretical percentages of exposed (maternal vaccination) and non-exposed (no maternal vaccination) with each health outcome,
- Different theoretical levels of exposed to unexposed ratio,
- 80% power and 95% confidence level,
- Per year of study duration

5.2.1.1 Sensitivity analysis

Three types of rates will be calculated:

1) Outcomes identified by sites pre-amendment
2) Outcomes identified by sites post-amendment
3) Outcomes based on additional information collected and interpreted at the backend (preterm birth based on data point GA, low birth weight based on data point birth weight, SGA based on data points sex and GA (and interpreted using chart), congenital microcephaly based on sex, GA and head circumference (and interpreted using chart; only for sites at which head circumference is routinely collected).

5.2.2 Primary study outcome 2: Applicability of GAIA case definition

5.2.2.1 Health outcomes

For each health outcome, applicability of GAIA case definition will be assessed by calculating the proportion of cases identified that can be classified[1] according to the GAIA case definition.

The denominator will be the number of cases identified for which the CRF was completed for one health outcome; the numerator will be the number of cases identified for that health outcome that can be classified according to the GAIA case definition.

The applicability of the GAIA case definitions for each health outcome will be reported overall, and stratified by site/country/region, and by health facility level (primary/secondary/tertiary).

5.2.2.2 Exposure: level of certainty for maternal immunisation

The applicability of the GAIA definition for maternal immunization will be assessed by calculating the proportion of vaccination mothers identified that can be classified according to the GAIA definition.

The denominator will be the number of vaccination mothers identified; the numerator will be the number of vaccination mothers identified that can be classified according to the GAIA definition.

The applicability of the GAIA definition will be reported overall, and stratified by site/country/region, by health facility level (primary/secondary/tertiary) and by type of data (paper vs. electronic).

The proportion of all mothers whose maternal vaccination status was assessed as exposed, non-exposed and unknown will be described.

5.3 Secondary study outcomes

5.3.1 Secondary study outcome 1 – Proportion of cases classified by level

5.3.1.1 Health outcomes

For each health outcome, the proportion of cases that can be classified according to each GAIA case definition level will be assessed.

Proportion of cases identified that can be classified by level

- For each health outcome, the proportion of cases identified that can be classified according to each GAIA case definition level will be assessed.

[1] In this document, 'classified' will mean 'classified according to the GAIA case definitions

- The denominator will be the number of cases *identified* for one outcome; the numerator will be the number of classified cases for each level of the GAIA case definition.

Proportion of cases classified by level

- For each health outcome, the proportion of cases classified by level will be calculated.
- The denominator will be the number of cases *classified* for one outcome for all levels; the numerator will be the number of classified cases for each level of the GAIA case definition.

These proportions will be reported overall, and stratified by site/country/region, and by health facility level (primary/secondary/tertiary).

5.3.1.2 Exposure to vaccination

For the exposure to maternal vaccination, the proportion of maternal vaccinations that can be classified according to each GAIA exposure definition level will be assessed.

Proportion of vaccinated mothers identified classified by level

- The proportion of maternal vaccinations identified that can be classified according to each GAIA definition level will be assessed.
- The denominator will be the number of *identified* maternal vaccinations; the numerator will be the number maternal vaccinations classified for each level of the GAIA definition.

Proportion of cases classified by level

- The proportion of vaccinated mothers classified by level will be calculated.
- The denominator will be the number of *classified* maternal vaccinations; the numerator will be the number of maternal vaccinations classified for each level of the GAIA definition.

These proportions will be reported overall, and stratified by country/region, and by health facility level (primary/secondary/tertiary).

5.3.2 Secondary study outcome 2 – Factors influencing applicability

It will be assessed whether the following factors influence GAIA case definition applicability:

- Health facility level (primary, secondary, tertiary/referral)
- Region
- Qualification of research staff to identify cases and to complete CRFs
- Availability of technical equipment (e.g. laboratory, ultrasound, type of scale etc.)

The proportion of cases identified that can be classified across all sites will be tabulated and then stratified by the factors above. A Chi-square test will be used to assess whether there are any significant differences between the categories.

5.4 Exploratory study outcomes

5.4.1 Missing elements to meet GAIA case definition

To better understand why GAIA case definitions are not met, the type of data elements that are required by the GAIA case definition but that are missing will be described.

For each health outcome, the reasons why identified cases could not be classified will be tabulated (e.g. four identified cases of neonatal death could not be classified because date of death was unknown).

If feasible, reasons will be grouped, and proportions will be calculated.

5.4.2 Missing elements to reach a better level of diagnostic certainty

To better understand why the highest levels of diagnostic certainty are not met, cases that are classifiable at the Lowest levels of diagnostic certainty (Level 3, 4), the type of data elements that are required to meet a better level of diagnostic certainty will be described.

For each health outcome, the reasons why Level 3 or 4 cases could not be classified as Level 1 or Level 2 will be tabulated.

5.5 Handling of missing data

Missing data will not be imputed, and the data will be analyzed as they are recorded in the eCRFs. Missing data will be tabulated and explored as described in the exploratory objectives.

6 Limitations

We have identified the following limitations for this study:

- Feasibility of data collection for GAIA case definitions are site-specific and may not be readily generalized to country-level or LMIC-level.
- In the absence of a GAIA definition for 'no maternal immunization', any mother without any evidence for and no history of vaccination will be considered not vaccinated during pregnancy. However, this may result in false-negative exposures.
- Health outcomes during the first month of life cannot be ascertained for children delivered at the site but who do not seek health care or who seek health care elsewhere. Therefore, the background rates may underestimate the true rates of the outcomes in the study populations.
- Children diagnosed with health outcomes of interest at the site but who were born elsewhere cannot be included as denominator information (number of deliveries) will not be available

7 Data reporting

7.1 Study Report

A study report based on the results obtained will be prepared. The final study report should be available within 6 months from collection of the last data point. The participating sites will be informed about the results when the report is finalized.

7.2 Dissemination strategy

The dissemination actions will comprise at least:

1. Publication of scientific papers in indexed international scientific journalsurce
2. Participation in scientific meetings and congresses, through oral communications, and posters
3. Oral presentation during the WHO annual Global Vaccine Safety Initiative meeting
4. Presentation of study results and submission of the final report to institutions involved

8 Study management

8.1 Data management

A data management plan (DMP) will be created before any data collection starts. This DMP will describe all functions, processes, and specifications for data collection, cleaning and validation. The eCRFs will include programmable checks to obtain immediate feedback if data are missing, out of range, illogical or potentially erroneous. Concurrent manual data review will be performed based on parameters dictated by the plan. Ad hoc queries will be generated within the EDC system and followed up for resolution.

8.2 Data entry/ EDC

An EDC system will be provided. The EDC system will consist of an android module and a web module. The android module will be pre-installed on tablets that will be distributed to the sites. The web module will be used for data processing and management by INCLEN and P95. The software will be developed using open source (PHP and MySQL). Sites will be provided with comprehensive training on the use of the EDC system, including eCRF completion guidelines.

User testing will be performed prior to deployment (see 8.1). The data collection tool functionality will be based on the CRFs.

Android module

The android module will be used at the sites for screening, recruitment, data collection through eCRFs, tracking, on-site quality check and validation. Skipping patterns and logical checks will be put in place. The android module can be used online and offline for data entry. The data collected by tablets will be uploaded in real-time to the server, at least once a day.

Web module

The web module will be used by the INCLEN and P95 central investigation team for central monitoring of site activities (e.g. tracking study progress), user management, data management (including checking, validating, cleaning), and archiving. The queries generated after logical checks (range and consistency checks) will be sent to the sites for resolution/ completion.

8.3 Source documents

In most cases, the source document will be the patient medical record. All source documents for all data to be collected during the study will be mapped site by site prior to data collection start. A document will be prepared with all data that needs to be

collected per outcome, where the exact source that will be used to provide the data will be specified. This will be requested to the study sites before study start and reviewed during the training session. In addition, it will be regularly updated if any changes occur during the course of the study. All original source documentation is expected to be stored at the site for the longest possible time required by local applicable regulations. The sites will be instructed to notify WHO before any destruction of medical records of study participants.

The tablet allows the optional scanning of source documents for organizational support of the site. This could help during query resolution, as well as allowing site primary investigators to verify data entered against these original source documents. Any scanned documents will only be accessible for authorized study personnel within the study site and it will not be possible to upload these to the central server. Access to such documents shall be restricted to site PI level based on user rights granted. No source documents shall be visible to persons outside the site.

8.4 Data security

General Data Protection Regulation (GDPR) regulation will apply [21].

EDC – android module

The modules will have user-specific login features and data protection measures to enable access and data entry considering the data safety and archival aspects. The system will have Secure Socket Layer (SSL) certificate with 256 bit SSL encryption during data transfer. Appropriate security system for data safety and confidentiality will be put in place.

EDC – web module

In-built data security processes will also be put in place.

The team will be provided login details and rights will be assigned for specific roles.

Sites PIs will also have access to supervisory web module from where they can view data of their site, and monitor study progress in real-time.

Data storage

Data will be saved on a secure server complying with data safety regulations for patient personal health information.

8.5 Data transfer procedure

Data will be saved directly on an online secure server (see 7.4) complying with patient data safety regulations. No data transfers will be required.

8.6 File retention and archiving

GDPR will be applied to data storage. Data will be stored for 5 years.

Good Epidemiological Practice (GEP) will be applied for retention of paper files at the sites [22]. Standards for data archiving will be applied.

8.7 Quality assurance and monitoring

Training

Face-to-face training on study protocol, field procedures and data collection (including proper completion of the CRFs), data entry in the EDC will be provided to the study teams of all sites prior study start.

Study initiation

During the first month after recruitment starts at each site, a weekly TC will be organized between the central team and the study site team to review the implementation of study procedures and EDC system, in order to identify and solve any issues. Standardized reports shall be generated and shared with the investigator team on a weekly basis (initially) to track study progress and data quality. Reports shall allow for monitoring site performance based on selected indicators. Poor performing sites shall be provided feedback (weekly at first, then on a monthly basis after network stabilization). This will be replaced by centralized monitoring from month 2.

Centralized monitoring

Centralized monitoring will be performed during the data collection through the EDC system or through Tableau. This will allow the identification of outliers or trends that may needs attention from the research team to identify and mitigate problems during the study period. Details of centralized monitoring will be outlined in the DMP.

For-cause monitoring visits

During the course of the study, for-cause on-site monitoring visits may be made, to check adherence to the protocol and quality of data collection. These visits will be organized for those sites for which performance issues are identified. Follow-up calls may be organized with study sites to address any pending issues.

The sites will permit study-related on-site monitoring and audits, providing direct access to data in the various data sources (documents) as required.

8.8 Changes to the protocol

Any changes to the protocol will be documented as amendments.

Amendments that impact the study objectives, procedures and participant safety will require submission to the WHO/ERC and all relevant independent ethics

committee/institutional review board (IEC/IRB) for approval. In this case, the amendment won't be implemented until approval has been obtained.

Minor amendments like change of staff in the study sites, will be intimated to the relevant IEC/IRB as appropriate.

Any amendment that may have an impact on the subject's agreement to participate in the study will require re-consent.

9 Ethical and Regulatory Considerations

All study procedures will be conducted according to the International Ethical Guidelines for health-related research involving humans (CIOMS, 2016) [15], under the principles of the Declaration of Helsinki [18] and considering local legislation on medical research in humans and data sharing from clinical records beyond national or administrative borders.

This is an observational study without medical intervention or change in the clinical and diagnostic capacity. Therefore, there is no direct benefit to the participants. Nevertheless, there are important potential societal benefits derived from this international vaccine safety study. The assessment of minimum detectable risk for the various health outcomes, gaps and deficiencies in the applicability of GAIA definitions, will pave the way for planning the next steps, in countries with limited capacity for assessment of adverse events following maternal vaccination.

The long-term goal is for countries to have an appropriate and sustainable public health surveillance system with capacity to collect quality data for monitoring vaccine safety. This study is a model for how the global community supports countries that lack their own capacity to perform surveillance.

9.1 Respecting participant autonomy

The study will utilise data collected as part of health care provision; no additional data collection specifically for the purpose of the study is envisaged. No data will be used, either alone or in conjunction with any other information, in any effort whatsoever to establish the individual identities of any of the participants from whom data were obtained. Details of data protection are spelt out in sections 8.4. Site can choose to implement one (or more) of three approaches to obtaining informed consent (Annex 2).

1. **Study-specific informed consent upon case identification.** In this scenario, mothers of identified cases would be approached for informed consent (Annex 6).
2. **Study-specific informed consent prior to case identification.** In this scenario, pregnant women would be approached for informed consent during antenatal care visits or upon admission to the maternity ward, prior to knowing whether their child would be eligible or not eligible for the study (Annex 6).
3. **Broad informed consent upon registration at the health facility, as per facility's standard operation procedures**. This scenario is only available to health facilities that routinely ask patients for informed consent to use their data for research purposes (Annex 6).

The study-specific informed consent forms will spell out the purpose of the data collection, the foreseeable uses of the data, the intended goal of such use, who has access to and the conditions and duration of data storage; the ways in which the participant can contact the custodian and remain informed about future use. For those mothers, not willing to share their data, an opt-out option will be provided. It will explain that their participation is completely voluntary and that they may choose to withdraw at any time during the study.

9.2 Study participant confidentiality

Confidentially of data will be maintained at all times according to global and local regulatory requirements. The only document with personal identifiers will be an excel sheet or paper register with minimum information relating to participants identity (detailed in section 3.8). This will be stored in the study site and will at no cost be shared outside the study site. Each site will take all reasonable measures to safeguard this document through password protection and restricted access only to authorised personnel. This document will be destroyed after the study report will be finalized.
The study data will be accessible only to the investigators selected to perform the quality and consistency checks and statistical analysis. WHO will be the data owner.

9.3 Independent Ethics Committee/ Institutional Review Board

WHO will ensure that the master protocol (and any amendments) with study sites specific details, are submitted to the WHO Research Ethics Review Committee for review and approval. Each participating centre will be responsible for submission of the study protocol and any amendment to local/national/ Independent Ethics Committees and/or Institutional Reviews Boards according to local requirements.

Any additional regulatory clearances, as required by the country law and regulations will be adhered to in respect of each participating country.

10 Expected Timelines

Month *planned*	Tasks
By December 2018	- Preparation of EDC system (eCRFs, user profiles)<br>- Data management plan<br>- Development of site-specific study procedures<br>- Obtaining WHO Research Ethics Review Committee and local approvals<br>- Preparation of administrative approvals
February – April 2019	- Workshops (training on protocol and EDC system)
May 2019 – August 2020	- Case Identification<br>- Data collection, Data monitoring, data cleaning
September – October 2020	- Data analysis<br>- Study report

11 References

[1] Keller-Stanislawski B, Englund JA, Kang G, Mangtani P, Neuzil K, Nohynek H, et al. Safety of immunization during pregnancy: a review of the evidence of selected inactivated and live attenuated vaccines. Vaccine. 2014;32:7057-64.
[2] WHO. WHO fact sheet no. 333: newborns: reducing mortality. 2016.
[3] Englund JA. Maternal immunization–Promises and concerns. Elsevier; 2015.
[4] Amirthalingam G, Andrews N, Campbell H, Ribeiro S, Kara E, Donegan K, et al. Effectiveness of maternal pertussis vaccination in England: an observational study. The Lancet. 2014;384:1521-8.
[5] Madhi SA, Cutland CL, Kuwanda L, Weinberg A, Hugo A, Jones S, et al. Influenza vaccination of pregnant women and protection of their infants. New England Journal of Medicine. 2014;371:918-31.
[6] Kochhar S. Harmonized Safety Monitoring of Immunization in Pregnancy- the Global Alignment of Immunization Safety Assessment in Pregnancy (GAIA) project. Impatient Optimists. Bill & Melinda Gates FoundationMarch 15, 2017.
[7] Chen RT, Moro PL, Bauwens J, Bonhoeffer J. Obstetrical and neonatal case definitions for immunization safety data. Vaccine. 2016;34:5991.
[8] Lackritz E, Stergachis A, Stepanchak M, Englund J, Tavares Da Silva F, Sevene E, et al. Maternal Immunization Safety Monitoring in Low- and Middle-Income Countries: A Roadmap for Program Development. Building an approach that is practical, affordable, and sustainable. In: Eve M. Lackritz, Andy Stergachis, Stepanchak M, editors.: Global Alliance to Prevent Prematurity and Stillbirth; 2017.
[9] Bonhoeffer J, Kohl K, Chen R, Duclos P, Heijbel H, Heininger U, et al. The Brighton Collaboration: addressing the need for standardized case definitions of adverse events following immunization (AEFI). Vaccine. 2002;21:298-302.
[10] GAIA consortium. GAIA - a global project.
[11] Bonhoeffer J, Kochhar S, Hirschfeld S, Heath PT, Jones CE, Bauwens J, et al. Global alignment of immunization safety assessment in pregnancy–The GAIA project. Vaccine. 2016;34:5993-7.
[12] Guillard-Maure C, Elango V, Black S, Perez-Vilar S, Castro JL, Bravo-Alcantara P, et al. Operational lessons learned in conducting a multi-country collaboration for vaccine safety signal verification and hypothesis testing: The global vaccine safety multi country collaboration initiative. Vaccine. 2017.
[13] Bonhoeffer J, DeSilva M, Muñoz F, Sell E, Marshall H, Tse Kawai A, et al. Congenital Microcephaly: Case Definition & Guidelines for Data Collection, Analysis, and Presentation of Safety Data after Maternal Immunisation. In: Collaboration B, editor. The GAIA project - Global Alignment of Immunisation Safety Assessment in Pregnancy. Submitted2016.
[14] Quinn J-A, Munoz FM, Gonik B, Frau L, Cutland C, Mallett-Moore T, et al. Preterm birth: Case definition & guidelines for data collection, analysis, and presentation of immunisation safety data. Vaccine. 2016;34:6047-56.
[15] Pathirana J, Muñoz FM, Abbing-Karahagopian V, Bhat N, Harris T, Kapoor A, et al. Neonatal death: Case definition & guidelines for data collection, analysis, and presentation of immunization safety data. Vaccine. 2016;34:6027-37.
[16] Cutland C, Lackritz E, Mallett-Moore T, Bardaji A, Chandrasekaran R, Lahariya C, et al. Low birth weight: Case definition & guidelines for data collection, analysis, and presentation of maternal immunization safety data. Vaccine. 2017;35:6492-500.

[17] Schlaudecker EP, Munoz FM, Bardají A, Boghossian NS, Khalil A, Mousa H, et al. Small for gestational age: Case definition & guidelines for data collection, analysis, and presentation of maternal immunisation safety data. Vaccine. 2017;35:6518-28.
[18] Vergnano S, Buttery J, Cailes B, Chandrasekaran R, Chiappini E, Clark E, et al. Neonatal infections: Case definition and guidelines for data collection, analysis, and presentation of immunisation safety data. Vaccine. 2016;34:6038-46.
[19] Da Silva FT, Gonik B, McMillan M, Keech C, Dellicour S, Bhange S, et al. Stillbirth: Case definition and guidelines for data collection, analysis, and presentation of maternal immunization safety data. Vaccine. 2016;34:6057-68.
[20] Brighton Collaboration. Virtual Institute Academy - Tools. Basel. 2018.
[21] Regulation (EU) 2016/679 of the European Parliament and of the Council of 27 April 2016 on the protection of natural persons with regard to the processing of personal data and on the free movement of such data, and repealing Directive 95/46/EC (General Data Protection Regulation). Official Journal of the European Union. 2016;L119:1-88
[22] IEA members at the IEA Meeting in Brazil in April 2007, edited by, Charles du V Florey, Neil Pearce, Susie Stewart. Good epidemiological practice (GEP) - IEA Guidelines for proper conduct of epidemiological research. 2007.
[23] Stuurman AL, Riera M, Lamprianou S, Perez-Vilar S, Anderson SA, Mangtani P, et al. Vaccine safety surveillance in pregnancy in low-and middle-income countries using GAIA case definitions: A feasibility assessment. Vaccine. 2018.

Annex I. Site Selection

The process of site selection is described in the following article[23], accessible here: https://www.sciencedirect.com/science/article/pii/S0264410X18312933?via%3Dihub

Annex II. Site descriptions

**Country: GHANA**		**National Authority involved in study oversight:** Vaccine Pharmacovigilance, Ghana Food and Drug Authority		
**Site Name**	**Site address**	**Principal Investigators**	**Details of study team - roles and responsibilities**	**Type of care at center; nr of deliveries per year**
St. Joseph's Hospital	Post Office Box 3, Jirapa, Upper West region	Dr. Richard Wodah-Seme	Miss Jane kundaanur (data collection maternity ward); Miss Esther Gagla (data collection NICU); Mr Augustine Allandu (OPD); Dr. Sulemana (clinical coordinator; case identification); Madam Judith Kpankori (finances); Mr yakubu Tifere (data entry); Miss Alfredina Dery (administration); Mr Michael Gansah (biomedical scientist); Miss Lucy Sarfo (ANC)	Secondary<br><br>1700
Ejisu Government Hospital	PO box 87, Ejisu-Juaben	Dr. Kwasi Baffour Gyimah	Dr. Joel Duah (Clinical coordinator); Ms. Juliana Addae (maternity I/C); Ms. Aboagyewaa (NICU); Thelma Daniels (ANC/PNC); Mawumenyo Kwawukume (Biostats); Stephen (RCH UNIT)	Secondary<br><br>1200
Tema General Hospital	Post Office Box 14, Tema	Dr Joseph Horatius Kojo Donkor	Mrs. Alice Amanor Kosa dik (labour ward); Mrs. Nelly Enyimah (NICU); Divine Etonam Doe (Disease control); Mrs. Martha Ampabin (RCH); Mrs Monica Afoakwa (PH); Mrs Rose Quaeyson(ANC)	Secondary<br><br>8500
Eastern Regional Hospital	P.O. box 201, Eastern Region Koforidua	Dr Seth Twum	Dr. Augusta Worlase Ama Kutor (identification of Neonatal infections, congenital microcephalies and neonatal deaths); Mrs. Philomina Mireku (identification of stillbirths and preterm birth at labour ward); Grace Adu-Larbi (data collection NICU and labour ward); Ester Ba-Iredine (immunization/vaccines exposure at RCH); Dr. Kwame Anim-Boamah (case confirmation); Cynthia Martey (Data collection paediatric ward).	Secondary<br><br>5000

Country: GHANA				
**Site Name**	**Principal Investigators**	**Identification of cases**	**Source documents**	**Informed Consent process**
St. Joseph's Hospital	Dr. Richard Wodah-Seme	Free text	Delivery registers at labour ward, admission and discharge registers at NICU and ANC. Antenatal record cards.	Broad consent at time of antenatal care.
Ejisu Government Hospital	Dr. Kwasi Baffour Gyimah	Free text	Folders and records from the units - NICU register (admissions and discharges, neonatal audit, delivery). Maternal pregnancy records.	Upon Case Identification
Tema General Hospital	Dr Joseph Horatius Kojo Donkor	Free text	Registers at the labour ward, Neonatal intensive care, Disease control unit. Reproductive and child unit, public health unit and Antenatal unit. Electronic system in the hospital to retrieve all folders. ANC registers	Upon Case Identification, following group information sessions at antenatal clinic
Eastern Regional Hospital	Dr Seth Twum	Free text and ICD10	Medical records and antenatal cards from the NICU, LABOUR WARD-still birth and preterm birth, PAEDIATRIC WARD. RCH UNIT- immunization registers for vaccines exposure	Broad consent at time of antenatal care.

**Country: INDIA**		**National Authority involved in study oversight:** The INCLEN Trust International		
**Site Name**	**Principal Investigators**	**Site address**	**Details of study team - roles and responsibilities**	**Type of care at center; nr of deliveries per year**
JSS Hospital	Dr Mandyam Ravi	Mahathma Gandhi Road, 570 004 - Mysore, Karnataka	Dr Prajwala, Dr Juny Sebastian, Dr Sujatha	Tertiary<br><br>5800
Grants Government Medical College and Sir JJ Group of Hospitals	Pr Lalit Sankhe	JJ Marg, Nagpada-Mumbai Central Off Jijabhoy Road, Maharashtra, Mumbai 400008	Dr. Chhaya Rajguru; Dr. Ashok Anand; Dr. Preeti Lewis; Dr. Nita Sutye; Dr. Sushant Mane	Tertiary<br><br>3300
Institute of Medical Sciences (IMS) and Sum Hospital	Dr Rachita Sarangi	K 8 Kalinga Nagar, Shampur, Bhubaneswar, Odisha 751003	Dr. Trimul subudhi (Neonatologist); Dr.J Bikrant Prusty (MD.Ped); Dr.Tapan Pattanayak (MD,O & G)	Tertiary<br><br>3500
Kasturba Medical College	Pr Leslie Lewis	Tiger Cir Rd, Madhav Nagar, Manipal, Karnataka 576104	Dr. Murulidhar Pai (Dept Head OBG); Dr. Prathap Kumar (OBG); Dr.Jyothi Shetty(OBG); Dr. Shymala(OBG); Dr. Sripad Hebbar (OBG)	Tertiary<br><br>2400
MP Shah Government Medical College	Pr Bhadresh Vyas	Bedi Rd, Indradeep Society Jamnagar Gujarat 361008	Dr. Nalini Anand; Dr. Maulik Shah; Mr. Nimesh Choksi	Tertiary<br><br>8400
Sher-i-Kashmir Institute of Medical Sciences (SKIMS)	Dr Javeed Iqbal Bhat	Soura, Srinagar, Jammu & Kashmir 190011	Dr. Qazi Iqbal; Dr. Rabiya; Dr. Syed Wajid Ali; Dr Bashir Ahmad Charoo	Tertiary<br><br>3000

Country: INDIA				
**Site Name**	**Principal Investigators**	**Identification of cases**	**Source documents**	**Informed Consent process**
JSS Hospital	Dr Mandyam Ravi	ICD10	Electronic and manual records. Health Information System - all labs and x-rays, discharge summaries, Manual records are case sheets, ward admission and discharge register, labour ward register. Maternal health record	Routine Institutional Broad IC for further research
Grants Government Medical College and Sir JJ Group of Hospitals	Pr Lalit Sankhe	Free text	Patient records. Immunization card.	Upon Case Identification
Institute of Medical Sciences (IMS) and Sum Hospital	Dr Rachita Sarangi	Free text	Patient records. Immunization card.	Upon Case Identification
Kasturba Medical College	Pr Leslie Lewis	Free text	Patient records. Immunization card.	Upon Case Identification
MP Shah Government Medical College	Pr Bhadresh Vyas	Free text	Patient records. Immunization card.	Upon Case Identification
Sher-i-Kashmir Institute of Medical Sciences (SKIMS)	Dr Javeed Iqbal Bhat	Free text	Patient records. Immunization card.	Upon Case Identification

Country: ISLAMIC REPUBLIC of IRAN			National Authority involved in study oversight:<br>EPI manager from the Communicable Diseases Control Department, as representative of vaccine immunization programme	
Site Name	Principal Investigators	Site address	Details of study team - roles and responsibilities	Type of care at center; nr of deliveries per year
Mahdieh Hospital	Dr Maryam Khoshnoud Shariati	Shoosh Square Fadaeiyan Eslam St, Tehran	Institution personnel for interview with mother, for filling in forms, for data entry.<br>Dr Dadkhah and Dr Afjeh (heads of NICU Department)	Tertiary<br><br>6000
Shohada Teaching Hospital	Dr Mahta Bassir	Tajrish square, Tehran	3 residents (CRF completion), 3 neonatologists and 2 gynaecologists (case identification), 3 MA and 2 GP (data entry), 1 bachelor medical records section; Dr. Ladan Ajori (Obyn)	Tertiary<br><br>1500
Akbar Abadi Hospital	Dr Maryam Rahimi	Molavi Baghferdos Station, Tehran	Institution personnel for interview with mother; Responsible for filling the special form; Responsible for entering information to computer; Responsible for supervision. Support from Dr Esteghamati	Tertiary<br><br>10000

Site Name	Principal Investigators	Identification of cases	Source documents	Informed Consent process
Mahdieh Hospital	Dr Maryam Khoshnoud Shariati	ICD10	Electronic database (with all records)	Routine Institutional Broad IC for further research
Shohada Teaching Hospital	Dr Mahta Bassir	Free text and ICD10	Maternity delivery records, NICU recording document, discharge diagnosis	Upon Case Identification
Akbar Abadi Hospital	Dr Maryam Rahimi	Free text and ICD10	Maternity delivery records, NICU recording document, discharge diagnosis	Upon Case Identification

Country: NEPAL		National Authority involved in study oversight:<br>Adverse Event Following Immunization (AEFI) Committee Chair, Nepal		
Site Name	Principal Investigators	Site address	Details of study team - roles and responsibilities	Type of care at center; nr of deliveries per year
Patan Academy of Health Sciences-PAHS \ Patan Hospital	Prof. Imran Ansari<br>Dr. Ganesh Shah	Lagankhel, Latipur. Mailing address PAHS: P. O. Box 26500, Kathmandu	2 Research Officers (data collection, recording and organization of data); study assistant (transport record files, maintenance of office, IT management, assisting research officers).	Tertiary<br><br>8000
B.P. Koirala Institute of Health Sciences	Dr Rupa Rajbhandari Singh<br>Prof.Dr. Nisha Keshari Bhatta	Ghopa Camp, Dharan-18	Dr Shyam Prasad Kafle (Asst. Professor, Paediatrics); Dr Mohan Chandra Regmi (Additional Prof. & HOD, OBS &Gyn); Prof. Nilam Adhikari; 2 research officers; 1 office assistant.	Tertiary<br><br>10000

Site Name	Principal Investigators	Identification of cases	Source documents	Informed Consent process
Patan Academy of Health Sciences-PAHS \ Patan Hospital	Prof. Imran Ansari<br>Dr. Ganesh Shah	Free text and ICD10	Record files. Mothers' medical chart	Upon Case Identification
B.P. Koirala Institute of Health Sciences	Dr Rupa Rajbhandari Singh<br>Prof.Dr. Nisha Keshari Bhatta	Free text, ICD10, keywords	Antenatal ward/Labour room/ Obs OT / MCH/ Postnatal: register and patient file. Ped. Adm./ discharge register, NICU/nursery/neonatal or Ped. Ward patient register/file. Patient OPD card and the register.	Upon Case Identification

Country: SPAIN			
**Site Name**	**Principal Investigators**	**Site address**	**Details of study team - roles and responsibilities**
FISABIO - Fundación para el Fomento de la Investigación Sanitaria y Biomédica de la Comunitat Valenciana, Hospital General Universitario de Castellón	Dr Javier Diez	Avda. de Catalunya, 21 - 46020 Valencia	Project Manager: Dr Alejandro Orrico-Sánchez<br>Nurses at the maternity ward, paediatricians in new-born room
FISABIO - Fundación para el Fomento de la Investigación Sanitaria y Biomédica de la Comunitat Valenciana, Hospital Universitario Doctor Peset, Valencia.	Dr Javier Diez	Avda. de Catalunya, 21 - 46020 Valencia	Project Manager: Dr Alejandro Orrico-Sánchez<br>Nurses at the maternity ward, paediatricians in new-born room
**Type of care at centre**	**Identification of cases**	**Informed Consent process**	**Source documents**
Secondary	Free text and identifying patients on the wards	Upon Case Identification	Medical chart. Vaccine registry.

**Country: TANZANIA**		**National Authority involved in study oversight:**<br>Clinical Trials and Pharmacovigilance, Tanzania Food and Drugs Authority		
**Site Name**	**Principal Investigators**	**Site address**	**Details of study team - roles and responsibilities**	**Type of care at center; nr of deliveries per year**
St.Francis Referral Hospital	Dr Elias Kweyamba	P.O.box 73, Ifakara Morogoro	Rose Chipa (Case identification, data collection and record in paper CRF at labour ward); Gideon Kipangua (Case identification, data collection and record in paper CRF at labour ward); Namsifu Lyimo (Case identification, data collection and record in paper CRF at Neonatal ward); Florence Ndauka (Case identification, data collection and record in paper CRF at Neonatal ward); Denis Nganda (Data verification an entry in electronic CRF)	Tertiary<br>4500
National Institute for Medical Research - Mbeya Medical Research Center	Dr Issa Sabi (NIMR-MMRC)<br>*Dr Ismail Macha- Mbeya Regional Referral Hospital (MRRH)*	P.O.box 2410, Mbeya	Dr Ismail Macha, site Co-PI for MRRH.	Tertiary<br>3500
National Institute for Medical Research - Mbeya Medical Research Center	Dr Issa Sabi (NIMR-MMRC)<br>*Dr. Rebecca Mokeha- Mbeya Zonal Referral Hospital (MZRH)*	P.O.box 2410, Mbeya	Dr Rebecca Mokeha, site Co-PI for MZRH.	Tertiary<br>10500

Country: TANZANIA				
**Site Name**	**Principal Investigators**	**Identification of cases**	**Source documents**	**Informed Consent process**
St.Francis Referral Hospital	Dr Elias Kweyamba	Free text	Registers in labour wards and Neonatal wards. Mother's Reproductive Health cards.	Upon Case Identification
National Institute for Medical Research - Mbeya Medical Research Center	Dr Issa Sabi (NIMR-MMRC) *Dr Ismail Macha- Mbeya Regional Referral Hospital (MRRH)*	Free text	Admission and discharge registers, patient notes, Ante natal cards.	Upon Case Identification
National Institute for Medical Research - Mbeya Medical Research Center	Dr Issa Sabi (NIMR-MMRC) *Dr. Rebecca Mokeha- Mbeya Zonal Referral Hospital (MZRH)*	Free text	Admission records, delivery register, report book in maternity ward, treatment books, ANC booklet, postnatal care, tetanus-toxoid registers.	Upon Case Identification

Country: ZIMBABWE		National Authorities involved in study oversight:<br>Pharmacovigilance and Clinical Trials Unit , Medicines Control Authority of Zimbabwe<br>Ministry of Health and Child Care		
**Site Name**	**Principal Investigators**	**Site address**	**Details of study team - roles and responsibilities**	**Type of care at center; nr of deliveries per year**
Mbare Polyclinic (Edith Opperman Maternity Hospital)	Dr. Prosper Chonzi	6876 Glen Norah, Harare	Head Nursing Services: Mrs Phillomina Chitando: Coordination of training, support and supervision and Coordination of research activities, provision of resources. Senior Health Promotion Officer- mobilization of clients for early ANC booking. Sister in Charge- supervision of care, Tetanus Toxoid vaccination to pregnant mothers, and case identification, data management, safe and secure record keeping.	Primary<br><br>5000
Mutare Provincial Hospital	Dr Jaensch<br>Dorcas Masanga-Mutede	Robert Mugabe way, Box 30, Mutare	Dr Matron (supervises team in maternity and neonatal unit); Sister in charge maternity (identification of cases in maternity for example still births, extracting data); Health information officer (Data entry, data management)	Tertiary<br><br>4600

Site Name	Principal Investigators	Identification of cases	Source documents	Informed Consent process
Mbare Polyclinic (Edith Opperman Maternity Hospital)	Dr. Prosper Chonzi	Free text	Data for the preterm and maternal factors will be identified from antenatal care, delivery, postnatal care, TT registers and report books. Maternal data for stillbirth cases will be extracted from ANC, delivery registers.	Upon Case Identification, following group information sessions at antenatal clinic
Mutare Provincial Hospital	Dr Jaensch<br>Dorcas Masanga-Mutede	Free text	Maternity delivery register, Perinatal death register, Special Care Baby Unit (SCBU) admission register, children’s ward admission register. ANC card.	Broad consent at time of maternity admission

Annex III. GAIA case definitions

Congenital microcephaly

Post-natally diagnosed congenital microcephaly

Level 1 of diagnostic certainty

1. Live birth, stillbirth, or spontaneous or therapeutic abortion of at least 24 weeks of Gestational Age (GA)~
   AND
2. HC 2 SD below mean or <3 percentile according to GA and gender, using appropriate standardized reference charts for the population (e.g., WHO growth reference charts if GA ≥37 weeks and Intergrowth-21st reference charts for GA 24–36 weeks)
   AND
3. Measured between 24 and 36 h after birth or end of pregnancy.

~GA assessed based on certain LMP with confirmatory 1st trimester or 2nd trimester US scan, IUI, or embryo transfer date.

Level 2A of diagnostic certainty

1. Live birth, stillbirth, or spontaneous or therapeutic abortion of at least 24 weeks of GA~
   AND
2. HC 2 SD below mean or <3 percentile according to GA and gender, using appropriate standardized reference charts for the population (e.g., WHO growth reference charts if GA ≥37 weeks and Intergrowth-21st reference charts for GA 24–36 weeks)
   AND
3. Measured within the first 24 h § OR Measured >36 h and up to 6 weeks after birth or end of pregnancy with no apparent post-natal insult resulting in microcephaly

~GA assessed based on certain LMP with confirmatory 1st trimester or 2nd trimester US scan, IUI, or embryo transfer date

Level 2B of diagnostic certainty

1. Live birth, stillbirth, or spontaneous or therapeutic abortion of at least 24 weeks of GA~
   AND
2. HC 2 SD below mean or <3 percentile according to GA and gender, using appropriate standardized reference charts for the population (e.g., WHO growth reference charts if GA ≥37 weeks and Intergrowth-21st reference charts for GA 24–36 weeks)
   AND

3. Measured within the first 24 h § OR Measured >36 h and up to 6 weeks after birth or end of pregnancy with no apparent post-natal insult resulting in microcephaly

~GA assessed based on uncertain LMP with 2nd trimester US scan
§Take into account the variability in this period based on molding of the head

<u>Level 3A of diagnostic certainty</u>

1. Live birth, stillbirth, or spontaneous or therapeutic abortion of at least 24 weeks of GA~
   AND
2. HC 2 SD below mean or <3 percentile according to GA and gender, using appropriate standardized reference charts for the population (e.g., WHO growth reference charts if GA ≥37 weeks and Intergrowth-21st reference charts for GA 24–36 weeks)
   AND
3. Measured up to 6 weeks after birth or end of pregnancy with no apparent post-natal insult resulting in microcephaly

~GA based on LMP without confirmatory 1st or 2nd trimester ultrasound

<u>Level 3B of diagnostic certainty</u>

1. Live birth, stillbirth, or spontaneous or therapeutic abortion
   AND
2. Case meets criteria for microcephaly using a validated algorithm: 1 inpatient diagnosis OR 2 outpatient diagnoses OR 1 outpatient diagnosis AND death in first year using the following diagnostic codes ICD-9-CM code 742.1 or ICD-10-CM code Q02

<u>Level 4 of diagnostic certainty</u>

1. Live birth, stillbirth, or spontaneous or therapeutic abortion
   AND
2. Diagnosis of congenital microcephaly based on physical inspection without HC measurement OR Diagnosis of congenital microcephaly based on ICD-9-CM orICD-10-CM code that does not meet validated algorithm criteria above.

Low birth weight

<u>Level 1 of diagnostic certainty</u>

Newborn infant weighed within 24 h of birth
AND
Use electronic scale which is graduated to 10 g
AND

Scale is calibrated at least once a year
AND
Scale placed on level, hard surface
AND
Scale tared to zero grams
AND
Weight recorded as <2500 g
OR
Birth weight recorded as <2500 g
AND
Birth weight assessed as per health care facility's standard operating procedure, which fulfills criteria 1 to 5 of LOC1

Level 2 of diagnostic certainty

Newborn infant weighed within 24 h of birth
AND
Scale (electronic/spring) is graduated to at least 50 g
AND
Scale is calibrated at least once a year, or more often if moved
AND
Scale tared to zero grams or 0.00 kg
AND
Weight recorded as <2500 g
OR
Birth weight recorded as <2500 g
AND
Birth weight assessed as per health care facility's standard operating procedure, which fulfills criteria 1 to 4 of LOC2

Scale used: could be electronic or spring scale, including color-coded scale.

Level 3 of diagnostic certainty

Newborn infant weighed on day 1 or 2 of life (first 48 h of life)
AND
Weight measured using dial/spring/color-coded scale
AND
Weight assessed as <2500 g

Level 4 of diagnostic certainty

Newborn infant 'weight' assessed on day 1 or 2 of life (first 48 h of life)
AND
Proxy measure of birth weight used

AND
Weight CATEGORY assessed as <2500 g

Neonatal death

Neonatal death in a non-viable live birth

Level 1 of diagnostic certainty

1. Live born infant
AND
2. Gestational age <22 weeks (GA level of certainty = 1) [28]
OR
3. Birth weight <500 g
AND
4. Death of infant in first 28 days of life
AND
5. Medically-confirmed death

Level 2 of diagnostic certainty

1. Live born infant
AND
2. Gestational age/size of newborn assessed as at least one of:
    a. Gestational age <22 weeks (GA Level of Certainty = 1 OR 2) [28]
    b. Birth weight <500 g

AND
3. Death of infant in first 28 days of life
AND
4. Medically-confirmed death OR non-medically-confirmed death [3]

Level 3 of diagnostic certainty

1. Live born infant [1]
AND
2. Gestational age <5 months according to parent/family member/delivery attendant (GA Level of Certainty = 2 OR 3) [28]
AND
3. Death of infant in first 28 days of life
AND
4. Medically-confirmed death [2] OR non-medically-confirmed death

Neonatal death in an extremely preterm live birth
MAY apply to LMIC- or may be non-viable in LMIC (Gestational age 22 to <28 weeks; should fit in with GA of stillbirth definition in region)

Level 1 of diagnostic certainty

1. Live born infant
AND
2. Gestational age ≥22 and <28 weeks (GA Level of Certainty = 1) [28]
OR
3. Birth weight ≥500 g but <1000 g
AND
4. Death of infant in first 28 days of life
AND
5. Medically-confirmed death

Level 2 of diagnostic certainty

1. Live born infant
AND
2. Gestational age/size of newborn assesses as one or more of:

    a. Gestational age ≥22 and <28 weeks (GA Level of Certainty = 1 OR 2) [28]
    b. Birth weight ≥500 g but <1000 g

AND
3. Death of infant in first 28 days of life
AND
4. Medically-confirmed death OR non-medically-confirmed death

Level 3 of diagnostic certainty

1. Live born infant
AND
2. Gestational age ≥5 months but <7 months according to neonate's parent (mother/father)/family member/delivery attendant (GA Level of Certainty = 2 OR 3) [28]
AND
3. Death of infant in first 28 days of life
AND
4. Medically-confirmed death OR non-medically-confirmed death

Neonatal death in a preterm live birth (gestational age ≥28 to <37 weeks)

Level 1 of diagnostic certainty

1. Live born infant
AND
2. Gestational age ≥28 and <37 weeks (Level of Certainty = 1) [28]
OR
3. Birth weight ≥1000 g but <2500 g

AND
4. Death of infant in first 28 days of life
AND
5. Medically-confirmed death

<u>Level 2 of diagnostic certainty</u>

1. Live born infant
AND

2. Gestational age/size of newborn assesses as one or more of:

a. Gestational age ≥28 and <37 weeks (GA Level of Certainty = 1 OR 2) [28]

b. Birth weight ≥1000 g but <2500 g

AND
3. Death of infant in first 28 days of life
AND
4. Medically-confirmed death OR non-medically-confirmed death

<u>Level 3 of diagnostic certainty</u>
(MAY apply to LMIC- or may be non-viable in LMIC)

1. Live born infant
AND
2. Gestational age ≥7 months but <9 months according to parent/family member/delivery attendant (GA Level of Certainty = 2 OR 3) [28]
AND
3. Death of infant in first 28 days of life
AND
4. Medically-confirmed death OR non-medically-confirmed death

Neonatal death in a term live birth

<u>Level 1 of diagnostic certainty</u>

1. Live born infant
AND
2. Gestational age ≥37 weeks (GA Level of Certainty = 1) [28]
AND
3. Birth weight >2500 g
OR 4. Documented intra-uterine growth retardation if ≤2500 g
AND

5. Death of infant in first 28 days of life AND

6. Medically-confirmed death

Level 2 of diagnostic certainty

1. Live born infant
AND
2. Gestational age/size of newborn assesses as one or more of:

a. Gestational age ≥37 weeks (GA Level of Certainty = 1 OR 2) [28]

b. Birth weight ≥2500 g
AND
3. Death of infant in first 28 days of life
AND
4. Medically-confirmed death OR non-medically-confirmed death which is confirmed by examination by (by at least) non-medically-trained attendant (e.g. undertaker, community member)

Level 3 of diagnostic certainty
(apply to LMIC)

1. Live born infant
AND
2. Gestational age ≥9 months according to parent/family member/delivery attendant (GA Level of Certainty = 2 OR 3) [28]
AND
3. Death of infant in first 28 days of life
AND
4. Medically-confirmed death OR non-medically-confirmed death

Neonatal infection

Neonatal invasive blood stream infections: bacterial/fungal/viral.

LEVEL 1	LEVEL 2	LEVEL 3 [22]
Recognised pathogen[a] identified using a validated method and from a normally sterile site[b]<br><br>If an organism normally considered non-pathogenic is isolated from blood cultures[a]: Level 1 requires its identification from at least 2 blood cultures taken from two different sites, or at 2 different times, PLUS 1 of the criteria as per level 2 of evidence	Not meeting Level 1 of evidence<br><br>**AND**<br><br>3 or more criteria:<br>• Temperature ≥37.5 °C or <35.5 °C[f]<br>• Tachycardia[d] or new or more frequent episodes of bradycardia[d]<br>• New or more frequent episodes of apnea[d] or increased oxygen requirement or increased requirement for ventilatory support<br>• Lethargy or moving only when stimulated or hypotonia or irritability<br>• Difficulty in feeding or abdominal distention<br>• Pallor or poor perfusion[d] or hypotension[d]<br>• Abnormal White Cell Count[d] or I/T ratio >0.2<br>• Abnormal platelet count [d]<br>• Increased[e] inflammatory markers (CRP, procalcitonin)<br>• Metabolic acidosis as defined by a base excess (BE)[d]	Not meeting Level 1 or 2 of evidence<br><br>**AND**<br><br>2 or more of the following criteria:<br>• Temperature ≥37.5 °C or <35.5 °C[f]<br>• Tachypnea[d] or severe chest indrawing or grunting or cyanosis<br>• Change in level of activity<br>• History of feeding difficulty<br>• History of convulsions

[a] See list of pathogens and non-pathogens in Appendix 1.

[b] Sterile site: blood, sterile urine (catheter urine or supra-pubic aspirate), pleural fluid, ascitic fluid, broncho-alveolar lavage, bone biopsy, synovial fluid.

[d] *Definitions*: Apnea: pause in breathing >20 s; CRP or calcitonin levels above the local normal standards; Tachypnea/fast breathing: respiratory rate >60 breaths per minute; Tachycardia: heart rate >180 beats per minute; Bradycardia: heart rate <100 beats per minute; c Poor perfusion: CRT >2. d 4000 or >20,000 × 10^9 cells/L; Low Platelets/Thrombocytopenia: <100,000 × 10^9/L; Metabolic acidosis: <−10 mmol/L (−10 mEq/L)

[e] Increased according to locally defined and validated reference ranges.

[f] Also refer to Brighton collaboration case definition for fever [23].

Respiratory bacterial/fungal/viral infection.

LEVEL 1	LEVEL 2	LEVEL 3 [24,25]
New or progressive or persistent infiltrate or shadowing or fluid in the intrapleural cavity or interlobar fissure on chest X-ray	New or progressive or persistent infiltrate or shadowing or fluid in the intrapleural cavity or interlobar fissure on chest X-ray	**2 or more criteria:**<br>Difficulty in breathing/Tachypnea[c]
AND	**AND 4 or more criteria:**	• Severe chest indrawing
Recognised virus[c] identified using a validated assay from an upper respiratory sample	• Temperature ≥37.5 °C or <35.5 °C[e]	• Nasal flaring
OR	• Tachypnea[c] or Nasal flaring or Chest indrawing or Grunting	• Grunting
Recognised pathogen[a] identified using a validated method and from a normally sterile site[b]		• Wheezing
**AND 3 or more criteria:**	• Desaturations or increased oxygen requirements or increased ventilator requirements or oxygen saturation <95%	• Stridor
• Temperature ≥37.5 °C or <35.5 °C[e]	• Apneas[c]	• Fever
• Tachypnea[c] or Nasal flaring or Chest indrawing or Grunting	• Increased respiratory secretions or Increased suctioning requirements	
• Desaturations or increased oxygen requirements or increased ventilator requirements or oxygen saturation <95%	• Cough or wheeze or crepitations	
• Apneas[c]	• Increased CRP or procalcitonin[d]	
• Increased respiratory secretions or Increased suctioning requirements		
• Cough or wheeze or crepitations		
• Increased CRP or procalcitonin[d]		

[a] See list of pathogens and non-pathogens in Appendix 1.
[b] Sterile site: blood, sterile urine (catheter urine or supra-pubic aspirate), pleural fluid, ascitic fluid, broncho-alveolar lavage, bone biopsy, synovial fluid.
[c] See list of definitions in Table 1.
[d] Increased according to locally defined and validated reference ranges.
[e] Also refer to Brighton collaboration case definition for fever [23].

Bacterial/fungal/viral meningitis.

LEVEL 1	LEVEL 2	LEVEL 3a	LEVEL 3b
Recognised pathogen[a] identified using a validated method from cerebrospinal fluid (CSF)<br><br>If an organism normally considered non-pathogenic is identified from the CSF, LEVEL 1 of evidence additionally requires all LEVEL 2 criteria: i.e. CSF pleocytosis AND temperature criteria AND 1 or more clinical criteria	CSF pleocytosis[d] OR positive IgM antibodies to a specific pathogen in the CSF<br><br>**AND**<br><br>Recognised pathogen[a] identified using a validated method from a normally sterile site[b] (other than CSF)<br><br>**AND**<br><br>Temperature ≥37.5 °C or <35.5 °C[c]<br><br>**AND**<br><br>1 or more criteria:<br>• History of convulsions<br>• Lethargy or irritability<br>• Coma<br>• Apnea[d]<br>• Bulging fontanel<br>• Neck stiffness	CSF pleocytosis[d]<br><br>**AND**<br><br>NO d pathogen[a] identified using a validated method from a normally sterile site[b]<br><br>**AND**<br><br>Temperature ≥37.5 °C or <35.5 °C[c]<br><br>**AND**<br><br>3 or more criteria:<br>• History of convulsions<br>• Lethargy or irritability<br>• Coma<br>• Apnea[d]<br>• Bulging fontanel<br>• Neck stiffness	No lumbar puncture done or no sample available<br><br>**AND**<br><br>Temperature ≥37.5 °C or <35.5 °C[c]<br><br>**AND**<br><br>4 or more criteria:<br>• History of convulsions<br>• Lethargy or irritability<br>• Coma<br>• Apnea[d]<br>• Bulging fontanel<br>• Neck stiffness

[a] See list of pathogens and non-pathogens in Appendix 1.
[b] Sterile site: blood, sterile urine (catheter urine or supra-pubic aspirate), pleural fluid, ascitic fluid, broncho-alveolar lavage, bone biopsy, synovial fluid.
[c] Also refer to Brighton collaboration case definition for fever [23].
[d] CSF pleocytosis: ≥20 cells/mm^3 for <28 day-olds and ≥10 cells/mm^3 for 29–89 day-olds. # i–89 day-olds.

Preterm birth

Level 1: (highest level of certainty)

1. Certain LMP* or intrauterine insemination (IUI) date or embryo transfer (ET) date with confirmatory 1st trimester scan (————————————13 6/7 weeks).
OR
2. 1st trimester scan (————————————13 6/7 weeks).

Level 2A

1. Certain LMP* with 2nd trimester scan (14 0/7 weeks to 27 6/7 weeks). If LMP and U/S do not correlate, default to U/S GA assessment.
OR
2. Certain LMP*with1st trimester physical examination.

Level 2B
Uncertain LMP with 2nd trimester scan (14 0/7 weeks to 27_6/7 weeks).

Level 3A

1. Certain LMPwith3rd trimester scan – 28 0/7 weeks +.
OR
2. Certain LMP with confirmatory 2nd trimester FH.
OR
3. Certain LMP with birth weight.
OR
4. Uncertain LMP with 1st trimester physical examination.

Level 3B

1. Uncertain LMP with FH.
OR
2. Uncertain LMP with newborn physical assessment.
OR
3. Uncertain LMP with Birth weight.

Small for gestational age[2]

SGA (small for gestational age) definition: weight below 10th percentile for gestational age as assessed against a validated global, regional or local standard.

Level 1 of diagnostic certainty

- Weight below 10th percentile for gestational age
  AND

[2] The case definition should be applied when there is no clear alternative diagnosis for the reported event to account for the combination of symptoms.

- The following used in assessment of weight:
  - o Newborn weighed within 24 h of birth
  - o Weight assessed using a calibrated electronic scale with 10 g resolution

  AND
- The following for assessment of gestational age:
  - o Certain LMP or IUI or embryo transfer date AND confirmatory ultrasound in first trimester

  OR
  - o First trimester ultrasound

<u>Level 2A of diagnostic certainty</u>

- Weight below 10th percentile for gestational age

  AND
- The following used in assessment of weight
  - o Newborn weighed within 24 h of birth on any scale with a <50 g resolution, tared to zero and calibrated

  AND
- The following for assessment of gestational age:
  - o Certain LMP with first or second trimester ultrasound

  OR
  - o Certain LMP with first trimester physical exam[3]

<u>Level 2B of diagnostic certainty</u>

- Weight below 10th percentile for gestational age

  AND
- The following used in assessment of weight
  - o Newborn weighed within 24 h of birth on any scale with a <50 g resolution, tared to zero and calibrated

  AND
- The following assessment of gestational age
  - o Uncertain LMP with second trimester ultrasound

<u>Level 3A of diagnostic certainty</u>

- Weight below 10th percentile for gestational age

  AND
- The following used in assessment of weight
  - o Infant weighed within the first 48 h of life
  - o Newborn weighed on any scale with a <50 g resolution, tared to zero and calibrated

  AND

[3] If Physical exam and ultrasound do not correlate, default to ultrasound.

- The following assessment of gestational age
  o Certain LMP with third trimester ultrasound
  OR
  o Certain LMP with confirmatory 2nd trimester fundal height
  OR
  o Certain LMP with birthweight
  OR
  o Uncertain LMP with first trimester physical exam

Level 3B of diagnostic certainty

- Weight below 10th percentile for gestational age
  AND
- The following used in assessment of weight
  o Infant weighed within the first 48 h of life
  o Newborn weight assessed by measuring the difference between an adult holding the infant and the adult being weighed alone on any scale
  AND
- The following assessment of gestational age
  o Uncertain LMP with fundal height
  OR
  o Uncertain LMP with newborn physical assessment
  OR
  o Uncertain LMP with birthweight

Level 4 of diagnostic certainty

- Baby noted to be small, but no actual weight
- Baby with GA assessed only by infant examination
- Diagnosis extracted from billing codes or chart, with no documentation of actual birth weight or GA

Level 5 of diagnostic certainty

- No evidence of SGA or a confirmed diagnosis other than SGA

Stillbirth

Antepartum stillbirth

Fetal death occurs prior to the evidence of labor.

Level 1

•Delivery of an infant with no of signs of life at birth (No spontaneous movements, no umbilical cord pulse, no heartbeat, no respirations, Apgar score of 0 at 1 and 5 min) determined by physical examination after delivery (with or without electronic monitoring of heart rate, respiratory

rate, and pulse oximetry).
AND
•Prenatal ultrasound examination documenting lack of fetal cardiac activity or movement before the onset of labor.
OR
•Auscultation for fetal heart tones (using electronic devices or non-electronic devices) documenting lack of fetal heartbeat.
AND
•Maternal report of lack of fetal movement for 24 h or more.
OR
•Maternal physical examination confirming lack of fetal movement.
OR
•Radiology findings consistent with intrauterine fetal death.
AND
•Attended delivery followed by fetal physical examination after birth consistent with antepartum death, by obstetrician, neonatologist, pediatrician, maternal-fetal medicine specialist, or pathologist. In the setting where access to a specialist is not feasible, diagnosis by a health care provider trained or experienced to make the diagnosis is acceptable (e.g. general practice physician, mid-wife, nurse practitioner, a physician's assistant or other qualified trained practitioner).
OR
•Fetal/placental pathology report consistent with antepartum death.
AND
•Gestational age within pre-defined range for selected stillbirth definition as assessed by maternal and/or fetal parameters (Level 1 or 2 in GA assessment algorithm).

<u>Level 2</u>

•Delivery of an infant with no of signs of life at birth (No spontaneous movements, no umbilical cord pulse, no heartbeat, no respirations, Apgar score of 0 at 1 and 5 min) determined physical examination after delivery.

AND
•Maternal report of lack of fetal movement for 24 h or more.
OR
•Maternal physical examination confirming lack of fetal movement.
OR
•Auscultation for fetal heart tones (using electronic or nonelectronic devices) documenting lack of fetal heartbeat.
AND
•Attended delivery followed by physical examination after birth consistent with antepartum death, by specialist or qualified trained practitioner

appropriate to the health care setting.
OR
•Fetal/placental pathology report consistent with antepartum death.
AND
•Gestational age within pre-defined range for selected stillbirth definition as assessed by maternal and/or fetal parameters (Level 1–2 in GA assessment algorithm).

Level 3

•Delivery of an infant reported to have no of signs of life at birth (No spontaneous movements, no umbilical cord pulse, no heartbeat, no cry or spontaneous respirations, no chest movement, and whole body cyanosis).
AND
•Maternal report of lack of fetal movement for 24 h or more prior to delivery.
OR
•Report of auscultation for fetal heart tones (using electronic or non-electronic devices) documenting lack of fetal heartbeat.
AND
•Non-attended delivery followed by physical examination of the fetus after birth consistent with antepartum death by a health care professional appropriate to the level of standard of care in the health care setting.
OR
•Verbal history by a trained health care provider, non-medical witness or the mother of a fetus born with no signs of life or unresponsive to resuscitation efforts immediately after birth and with physical features consistent with antepartum death.
AND
•Gestational age within pre-defined range for selected stillbirth definition as assessed by maternal and/or fetal parameters (Level 2–3 in GA assessment algorithm).

Level 4

•Report of stillbirth but fetus is not available for physical examination after birth (no objective assessment can be made).
•Maternal information insufficient to assess gestational age.

Intrapartum stillbirth

Fetal death occurs during labor and before delivery

Level 1

•Delivery of an infant with no of signs of life at birth, including: No spontaneous movements, no umbilical cord pulse, no heartbeat, no

respirations, and Apgar score of 0 at 1 and 5 min.
•Determination of the absence of signs of life is made by physical examination after delivery, with or without electronic monitoring of heart rate, respiratory rate, and pulse oximetry.
AND
•Evidence of live fetus prior to onset of labor (documentation of fetal movement and of fetal heart tones by ultrasound prior to onset of labor) (Note: in the absence of evidence of a live fetus prior to the onset of labor, the fetal death should be reported as a stillbirth or an antepartum stillbirth).
AND
•Attended delivery followed by physical examination after birth consistent with intrapartum death by obstetrician, neonatologist, pediatrician, maternal-fetal medicine specialist, pathologist. In the setting where access to a specialist is not feasible, diagnosis by a health care provider trained or experienced to make the diagnosis is acceptable (e.g. general practice physician, mid-wife, or other qualified trained practitioner).
AND
•Gestational age within pre-defined range for selected stillbirth definition as assessed by maternal and/or fetal-neonatal parameters (Level 1 in GA assessment algorithm)

Level 2

•Delivery of an infant with no of signs of life at birth, including: No spontaneous movements, no umbilical cord pulse, no heartbeat, no respirations, and Apgar score of 0 at 1 and 5 min.
•Determination of the absence of signs of life is made by physical examination after delivery, with or without electronic monitoring of heart rate, respiratory rate, and pulse oximetry OR documentation of lack of response to resuscitation efforts.
AND
•Evidence of live fetus prior to onset of labor (maternal report of fetal movement prior to onset of labor and documentation of fetal heart tones by auscultation or hand held Doppler) (Note: in the absence of evidence of a live fetus prior to the onset of labor, the fetal death should be reported as a stillbirth or an antepartum stillbirth).
AND
•Attended delivery followed by physical examination after birth consistent with intrapartum death by a health care professional appropriate to the level of standard of care in the health care setting.
AND
•Gestational age within pre-defined range for selected stillbirth definition as assessed by maternal and/or fetal parameters (Level 1–2 in GA assessment algorithm).

Level 3

•Delivery of an infant reported to have no of signs of life at birth, including: No spontaneous movements, no umbilical cord pulse, no heartbeat, no cry, no spontaneous respirations or chest movement, and whole body cyanosis.
AND
•Evidence of live fetus prior to onset of labor (maternal report of fetal movement prior to onset of labor OR auscultation of fetal heart tones) (Note: in the absence of evidence of a live fetus prior to the onset of labor, the fetal death should be reported as a stillbirth or an antepartum stillbirth).
AND
•Non-attended delivery followed by physical examination of the fetus after birth consistent with intrapartum death by a health care professional appropriate to the level of standard of care in the health care setting OR verbal history by a trained health care provider, non-medical witness or the mother of a fetus born with no signs of life or unresponsive to resuscitation efforts immediately after birth.
AND
•Gestational age within pre-defined range for selected stillbirth definition as assessed by maternal and/or fetal parameters (Level 2–3 in GA assessment algorithm).

Level 4

•Report of stillbirth but fetus is not available for physical examination after birth (no objective assessment can be made).
•Maternal information insufficient to assess gestational age.

Maternal immunization

Level 1 of diagnostic certainty

1. Woman confirmed as pregnant by positive pregnancy test or ultrasound confirmation

AND

2. Date/time of immunization of pregnant woman recorded in medical records by health care worker who administered/witnessed administration of vaccine

AND

3. Details of vaccine, including lot number, date of immunization

Level 2 of diagnostic certainty

1. Woman confirmed as pregnant by cessation of menstrual period and gravid uterus

AND

2. Date (at least month and year) of immunization of pregnant woman recorded in medical records by health care worker who administered/witnessed administration of vaccine

AND

3. Details of disease against which vaccinated

Level 3 of diagnostic certainty

1. Woman/medical attendant reports pregnancy AND

2. Woman reports receipt of vaccination during pregnancy, but no formal recording of immunization available.

Annex IV. ICD codes

Congenital microcephaly

ICD 10 codes

**Q00-Q00**	**Congenital malformations**
Q02	Microcephaly

ICD 9 codes

**740-759**	**Congenital anomalies**
742.1	Microcephalus

Low birth weight

ICD 10 codes

**P07**	**Disorders related to short gestation and low birth weight, not elsewhere classified**
P07.0	Extremely low birth weight
P07.1	Other low birth weight
P07.2	Extreme immaturity
P07.3	Other preterm infants

ICD 9 codes

**765**	**Disorders relating to short gestation and low birthweight**
765.0	Disorders relating to extreme immaturity of infant
765.00	Extreme immaturity, unspecified weight
765.01	Extreme immaturity, less than 500 grams
765.02	Extreme immaturity, 500 - 749 grams
765.03	Extreme immaturity, 750 - 999 grams
765.04	Extreme immaturity, 1,000 - 1,249 grams
765.05	Extreme immaturity, 1,250 - 1,499 grams
765.06	Extreme immaturity, 1,500 - 1,749 grams
765.07	Extreme immaturity, 1,750 - 1,999 grams
765.08	Extreme immaturity, 2,000 - 2,499 grams
765.09	Extreme immaturity, 2,500 grams and over
765.1	Disorders relating to other preterm infants
765.10	Other preterm infants, unspecified weight
765.11	Other preterm infants, less than 500 grams
765.12	Other preterm infants, 500 - 749 grams
765.13	Other preterm infants, 750 - 999 grams
765.14	Other preterm infants, 1,000 - 1,249 grams
765.15	Other preterm infants, 1,250 - 1,499 grams
765.16	Other preterm infants, 1,500 - 1,749 grams
765.17	Other preterm infants, 1,750 - 1,999 grams
765.18	Other preterm infants, 2,000 - 2,499 grams

765.19	Other preterm infants, 2,500 grams and over
765.20	Unspecified weeks of gestation
765.21	Less than 24 completed weeks of gestation
765.22	24 completed weeks of gestation
765.23	25 - 26 completed weeks of gestation
765.24	27 - 28 completed weeks of gestation
765.25	29 - 30 completed weeks of gestation
765.26	31 - 32 completed weeks of gestation
765.27	33 - 34 completed weeks of gestation
765.28	35 - 36 completed weeks of gestation

Neonatal infection

**ICD 10 code**	**Diagnosis**
A00-A09	Intestinal infectious diseases
A15-A19	Tuberculosis
A20-A28	Certain zoonotic bacterial diseases
A30-A49	Other bacterial diseases
A50	Congenital syphilis
A54	Gonococcal infection
A33	Tetanus neonatorum
P77	Necrotizing enterocolitis of fetus or newborn
A65-A69	Other spirochetal diseases
A70-A74	Other diseases caused by chlamydiae
A75-A79	Rickettsioses
A80-A89	Viral infections of the central nervous system
A90-A99	Arthropod-borne viral fevers and viral haemorrhagic fevers
B00-B09	Viral infections characterised by skin and mucous membrane lesions
B15-B19	Viral hepatitis
B20-B24	Human immunodeficiency virus (HIV) disease
B25-B34	Other viral diseases
B35-B49	Other viral diseases
B50-B64	Protozoal diseases
B65-B83	Helminthiases
B95-B98	Bacterial, viral and other infectious agents
B99	Other infectious diseases
**P23**	**Congenital pneumonia**
P23.0	Congenital pneumonia due to viral agent
P23.1	Congenital pneumonia due to Chlamydia
P23.2	Congenital pneumonia due to Staphylococcus
P23.3	Congenital pneumonia due to Streptococcus
P23.4	Congenital pneumonia due to Escherichia coli
P23.5	Congenital pneumonia due to Pseudomonas

P23.6	Congenital pneumonia due to other bacterial agents
P23.8	Congenital pneumonia due to other organisms
P23.9	Congenital pneumonia, unspecified
**P35**	**Congenital viral diseases**
P35.0	Congenital rubella syndrome
P35.1	Congenital cytomegalovirus infection
P35.2	Congenital herpesviral [herpes simplex] infection
P35.3	Congenital viral hepatitis
P35.8	Other congenital viral diseases
P35.9	Congenital viral disease, unspecified
**P36**	**Bacterial sepsis of newborn**
P36.0	Sepsis of newborn due to streptococcus, group B
P36.1	Sepsis of newborn due to other and unspecified streptococci
P36.2	Sepsis of newborn due to Staphylococcus aureus
P36.3	Sepsis of newborn due to other and unspecfied staphylococci
P36.4	Sepsis of newborn due to Escherichia coli
P36.5	Sepsis of newborn due to anaerobes
P36.8	Other bacterial sepsis of newborn
P36.9	Bacterial sepsis of newborn, unspecified
**P37**	**Other congenital infectious and parasitic diseases**
P37.0	Congenital tuberculosis
P37.1	Congenital toxoplasmosis
P37.2	Neonatal (disseminated) listeriosis
P37.3	Congenital falciparum malaria
P37.4	Other congenital malaria
P37.5	Neonatal candidiasis
P37.8	Other specified congenital infectious and parasitic diseases
P37.9	Congenital infectious and parasitic disease, unspecified
P38	Omphalitis of newborn with or without mild haemorrhage
**P39**	**Other infections specific to the perinatal period**
P39.0	Neonatal infective mastitis
P39.1	Neonatal conjunctivitis and dacryocystitis
P39.2	Intra-amniotic infection of fetus, not elsewhere classified
P39.3	Neonatal urinary tract infection
P39.4	Neonatal skin infection
P39.8	Other specified infections specific to the perinatal period
P39.9	Infection specific to the perinatal period, unspecified

ICD 9 Code	Diagnosis
001-009	Intestinal infectious diseases
010-018	Tuberculosis
020-027	Zoonotic bacterial diseases
030-041	Other bacterial diseases
042	Human Immunodeficiency virus

045-049	Poliomyelitis and other non-arthropod-borne viral diseases od the central nervous system
050-059	Viral diseases accompanied by exanthem
060-066	Arthropod-borne viral diseases
070-079	Other diseases de to viruses and Chlamydiae
080-088	Rickettsioses and other arthropod-borne diseases
090-099	Syphillis and other venereal diseases
100-104	Other spirochetal diseases
110-118	Mycoses
120-129	Helminthiases
130-136	Other infectious and parasitic diseases
770.0	Congenital pneumonia
**771**	**Infections specific to the perinatal period**
771.0	Congenital rubella
771.1	Congenital cytomegalovirus infection
771.2	Other congenital infections specific to the perinatal period
771.3	Tetanus neonatorum
771.4	Omphalitis of the newborn
771.5	Neonatal infective mastitis
771.6	Neonatal conjunctivitis and dacrycistitis
771.7	Neonatal Candida infection
771.8	Other infections specific to the perinatal period
771.81	Septicemia (sepsis) of newborn
771.82	Urinary tract infection of newborn
771.83	Bacteremia of newborn
771.89	Other infections specific to the perinatal period

Neonatal death

No specific codes for neonatal death other than those referring to stillbirth.

Preterm birth

**ICD 10 code**	**Diagnosis**
**O60**	**Preterm labour and delivery**
O60.0	Preterm labour without delivery
O60.1	Preterm spontaneous labour with preterm delivery
O60.2	Preterm spontaneous labour with term delivery
O60.3	Preterm delivery without spontaneous labour
**P05**	**Slow fetal growth and fetal malnutrition**
P05.0	Light for gestational age
P05.1	Small for gestational age
P05.2	Fetal malnutrition without mention of light or small for gestational age
P05.9	Slow fetal growth, unspecified
**P07**	**Disorders related to short gestation and low birth weight, not elsewhere classified**

P07.0	Extremely low birth weight
P07.1	Other low birth weight
P07.2	Extreme immaturity
P07.3	Other preterm infants

**ICD 9 code**	**Diagnosis**
**764**	**Slow fetal growth and fetal malnutrition**
764.0	Light-for-dates infant without mention of fetal malnutrition
764.00	Light-for-dates infant without mention of fetal malnutrition, unspecified wight
764.01	Light-for-dates infant without mention of fetal malnutrition, less than 500 grams
764.02	Light-for-dates infant without mention of fetal malnutrition, 500 - 749 grams
764.03	Light-for-dates infant without mention of fetal malnutrition, 750 - 999 grams
764.04	Light-for-dates infant without mention of fetal malnutrition, 1,000 - 1,249 grams
764.05	Light-for-dates infant without mention of fetal malnutrition, 1,250 - 1,499 grams
764.06	Light-for-dates infant without mention of fetal malnutrition, 1,500 - 1,749 grams
764.07	Light-for-dates infant without mention of fetal malnutrition, 1,750 - 1,999 grams
764.08	Light-for-dates infant without mention of fetal malnutrition, 2,000 - 2,499 grams
764.09	Light-for-dates infant without mention of fetal malnutrition, 2,500 grams and over
764.1	Light-for-dates infant with signs of fetal malnutrition
764.10	Light-for-dates infant with signs of fetal malnutrition, unspecified weight
764.11	Light-for-dates infant with signs of fetal malnutrition, less than 500 grams
764.12	Light-for-dates infant with signs of fetal malnutrition, 500 - 749 grams
764.13	Light-for-dates infant with signs of fetal malnutrition, 750 - 999 grams
764.14	Light-for-dates infant with signs of fetal malnutrition, 1,000 - 1,249 grams
764.15	Light-for-dates infant with signs of fetal malnutrition, 1,250 - 1,499 grams
764.16	Light-for-dates infant with signs of fetal malnutrition, 1,500 - 1,749 grams
764.17	Light-for-dates infant with signs of fetal malnutrition, 1,750 - 1,999 grams
764.18	Light-for-dates infant with signs of fetal malnutrition, 2,000 - 2,499 grams
764.19	Light-for-dates infant with signs of fetal malnutrition, 2,500 grams and over
764.2	Fetal malnutrition without mention of light-for-dates
764.20	Fetal malnutrition without mention of light-for-dates, unspecified weight
764.21	Fetal malnutrition without mention of light-for-dates, less than 500 grams
764.22	Fetal malnutrition without mention of light-for-dates, 500 - 749 grams
764.23	Fetal malnutrition without mention of light-for-dates, 750 - 999 grams
764.24	Fetal malnutrition without mention of light-for-dates, 1,000 - 1,249 grams
764.25	Fetal malnutrition without mention of light-for-dates, 1,250 - 1,499 grams
764.26	Fetal malnutrition without mention of light-for-dates, 1,500 - 1,749 grams
764.27	Fetal malnutrition without mention of light-for-dates, 1,750 - 1,999 grams
764.28	Fetal malnutrition without mention of light-for-dates, 2,000 - 2,499 grams
764.29	Fetal malnutrition without mention of light-for-dates, 2,500 grams and over
764.9	Fetal growth retardation unspecified
764.90	Fetal growth retardation unspecified, unspecified weight
764.91	Fetal growth retardation unspecified, less than 500 grams

764.92	Fetal growth retardation unspecified, 500 - 749 grams
764.93	Fetal growth retardation unspecified, 750 - 999 grams
764.94	Fetal growth retardation unspecified, 1,000 - 1,249 grams
764.95	Fetal growth retardation unspecified, 1,250 - 1,499 grams
764.96	Fetal growth retardation unspecified, 1,500 - 1,749 grams
764.97	Fetal growth retardation unspecified, 1,750 - 1,999 grams
764.98	Fetal growth retardation unspecified, 2,000 - 2,499 grams
764.99	Fetal growth retardation unspecified, 2,500 grams and over
**765**	**Disorders relating to short gestation and low birthweight**
765.0	Disorders relating to extreme immaturity of infant
765.00	Extreme immaturity, unspecified weight
765.01	Extreme immaturity, less than 500 grams
765.02	Extreme immaturity, 500 - 749 grams
765.03	Extreme immaturity, 750 - 999 grams
765.04	Extreme immaturity, 1,000 - 1,249 grams
765.05	Extreme immaturity, 1,250 - 1,499 grams
765.06	Extreme immaturity, 1,500 - 1,749 grams
765.07	Extreme immaturity, 1,750 - 1,999 grams
765.08	Extreme immaturity, 2,000 - 2,499 grams
765.09	Extreme immaturity, 2,500 grams and over
765.1	Disorders relating to other preterm infants
765.10	Other preterm infants, unspecified weight
765.11	Other preterm infants, less than 500 grams
765.12	Other preterm infants, 500 - 749 grams
765.13	Other preterm infants, 750 - 999 grams
765.14	Other preterm infants, 1,000 - 1,249 grams
765.15	Other preterm infants, 1,250 - 1,499 grams
765.16	Other preterm infants, 1,500 - 1,749 grams
765.17	Other preterm infants, 1,750 - 1,999 grams
765.18	Other preterm infants, 2,000 - 2,499 grams
765.19	Other preterm infants, 2,500 grams and over
765.20	Unspecified weeks of gestation
765.21	Less than 24 completed weeks of gestation
765.22	24 completed weeks of gestation
765.23	25 - 26 completed weeks of gestation
765.24	27 - 28 completed weeks of gestation
765.25	29 - 30 completed weeks of gestation
765.26	31 - 32 completed weeks of gestation
765.27	33 - 34 completed weeks of gestation
765.28	35 - 36 completed weeks of gestation

Stillbirth

ICD 10 code	Diagnosis
**Z37**	**Outcome of delivery**
Z37.1	Single stillbirth
Z37.3	Twins, one liveborn and one stillborn
Z37.4	Twins, both stillborn
Z37.6	Other multiple births, some liveborn
Z37.7	Other multiple births, all stillborn
Z37.9	Outcome of delivery, unspecified
P95	Fetal death of unspecified cause (incl deadborn fetus NOS, stillbirth NOS)

ICD 9 code	Diagnosis
**V27**	**Outcome of delivery**
V27.1	Outcome of delivery, single stillborn
V27.3	Outcome of delivery, twins, one liveborn and one stillborn
V27.4	Outcome of delivery, twins, both stillborn
V27.6	Outcome of delivery, other multiple birth, some liveborn
V27.7	Outcome of delivery, other multiple birth, all stillborn
V27.9	Outcome of delivery, unspecified outcome of delivery
779.9	Unspecified condition originating in the perinatal period (includes stillborn NEC)

Small for gestational age

ICD 10 codes	
**P05**	**Slow fetal growth and fetal malnutrition**
P05.0	Light for gestational age
P05.1	Small for gestational age
P05.2	Fetal malnutrition without mention of light or small for gestational age
P05.9	Slow fetal growth, unspecified

ICD 9 codes	
**764**	**Slow fetal growth and fetal malnutrition**
764.0	"Light-for-dates" infant without mention of fetal malnutrition
764.00	"Light-for-dates" infant without mention of fetal malnutrition, unspecified [weight]
764.01	"Light-for-dates" infant without mention of fetal malnutrition, less than 500 grams
764.02	"Light-for-dates" infant without mention of fetal malnutrition, 500 - 749 grams
764.03	"Light-for-dates" infant without mention of fetal malnutrition, 750 - 999 grams
764.04	"Light-for-dates" infant without mention of fetal malnutrition, 1,000 - 1,249 grams
764.05	"Light-for-dates" infant without mention of fetal malnutrition, 1,250 - 1,499 grams
764.06	"Light-for-dates" infant without mention of fetal malnutrition, 1,500 - 1,749 grams

764.07	"Light-for-dates" infant without mention of fetal malnutrition, 1,750 - 1,999 grams
764.08	"Light-for-dates" infant without mention of fetal malnutrition, 2,000 - 2,499 grams
764.09	"Light-for-dates" infant without mention of fetal malnutrition, 2,500 grams and over
764.1	"Light-for-dates" infant with signs of fetal malnutrition
764.10	"Light-for-dates" infant with signs of fetal malnutrition, unspecified [weight]
764.11	"Light-for-dates" infant with signs of fetal malnutrition, less than 500 grams
764.12	"Light-for-dates" infant with signs of fetal malnutrition, 500 - 749 grams
764.13	"Light-for-dates" infant with signs of fetal malnutrition, 750 - 999 grams
764.14	"Light-for-dates" infant with signs of fetal malnutrition, 1,000 - 1,249 grams
764.15	"Light-for-dates" infant with signs of fetal malnutrition, 1,250 - 1,499 grams
764.16	"Light-for-dates" infant with signs of fetal malnutrition, 1,500 - 1,749 grams
764.17	"Light-for-dates" infant with signs of fetal malnutrition, 1,750 - 1,999 grams
764.18	"Light-for-dates" infant with signs of fetal malnutrition, 2,000 - 2,499 grams
764.19	"Light-for-dates" infant with signs of fetal malnutrition, 2,500 grams and over
764.2	Fetal malnutrition without mention of "light-for-dates"
764.20	Fetal malnutrition without mention of "light-for-dates", unspecified [weight]
764.21	Fetal malnutrition without mention of "light-for-dates", less than 500 grams
764.22	Fetal malnutrition without mention of "light-for-dates", 500 - 749 grams
764.23	Fetal malnutrition without mention of "light-for-dates", 750 - 999 grams
764.24	Fetal malnutrition without mention of "light-for-dates", 1,000 - 1,249 grams
764.25	Fetal malnutrition without mention of "light-for-dates", 1,250 - 1,499 grams
764.26	Fetal malnutrition without mention of "light-for-dates", 1,500 - 1,749 grams
764.27	Fetal malnutrition without mention of "light-for-dates", 1,750 - 1,999 grams
764.28	Fetal malnutrition without mention of "light-for-dates", 2,000 - 2,499 grams
764.29	Fetal malnutrition without mention of "light-for-dates", 2,500 grams and over
764.9	Fetal growth retardation, unspecified
764.90	Fetal growth retardation, unspecified, unspecified [weight]
764.91	Fetal growth retardation, unspecified, less than 500 grams
764.92	Fetal growth retardation, unspecified, 500 - 749 grams
764.93	Fetal growth retardation, unspecified, 750 - 999 grams
764.94	Fetal growth retardation, unspecified, 1,000 - 1,249 grams
764.95	Fetal growth retardation, unspecified, 1,250 - 1,499 grams
764.96	Fetal growth retardation, unspecified, 1,500 - 1,749 grams
764.97	Fetal growth retardation, unspecified, 1,750 - 1,999 grams
764.98	Fetal growth retardation, unspecified, 2,000 - 2,499 grams
764.99	Fetal growth retardation, unspecified, 2,500 grams and over

Annex V. CRFs

General information

	DEMOGRAPHICS	
1	Date of birth	
**A**	**Status of live/stillborn**	
1	Sex	Male<br>Female<br>Indeterminate<br>Unknown
2	Gestational age	.... Weeks .... Day
3 a	Birth weight units	Grams<br>Kilograms<br>Pounds<br>Unknown
3a	Birth weight	*Please enter a number or enter 0 if unknown*
**B**	**Information on the delivery**	
1	Single or multiple deliveries?	Singleton<br>Twins<br>Triplets<br>Higher-order multiple births (specify)
2a	Were there any delivery-related complications?<br>*If no or unknown, skip to question 3.*	Yes<br>No<br>Unknown
2b	Delivery-related complications (select all that apply)	Antepartum/post-partum haemorrhage<br>Retained placenta<br>Obstructed labour<br>Pre-term labour<br>Vaginal bleeding<br>Death<br>Any other (specify)
3	Mode of delivery	Vaginal delivery<br>Assisted delivery<br>Caesarean delivery
4	Outcome of delivery for mother	Alive<br>Death
**C**	**Details of the Pregnancy**	
1	Age of the mother at time of delivery (in completed years)	.... Years
2a	Were there any pregnancy-related illnesses or	Yes<br>No

	complications for this pregnancy? *If no or unknown, skip the next question.*	Unknown
2b	Select all pregnancy-related illnesses or complications diagnosed	Diabetes<br>Hypertensive disorders<br>Anaemia in pregnancy (criteria for Hb to be updated)<br>Febrile illnesses (if yes, specify)<br>Substance use/abuse<br>Psychiatric conditions<br>Other, please specify<br>Unknown
	Past Obstetric History	*Please enter a number of unknown*<br>Gravida:<br>Para:<br>Abortions:

Preterm birth

PRETERM BIRTH

Preterm birth is defined as any birth before 37 weeks gestational age.

**PART 1 - GESTATIONAL AGE AT BIRTH**	
	**Please see Reference CRF GA**

Neonatal death

NEONATAL DEATH

Neonatal death is defined as death of a liveborn infant within the first 28 days of life.

	**Newborn case number**<br>**QUESTION**	**ANSWERS**
**PART 1 – NEONATAL DEATH**		
**A**	**Birth**	
1	Date of birth of the newborn	*Please enter DDMMYYYY or unknown.*
**B**	**Death**	
2a	Date of death of the newborn	*Please enter DDMMYYYY or unknown.*

2b	Was the death medically-confirmed, i.e. confirmed by suitable qualified medical or allied medical professional (including medical doctors, nurses, paramedics)	Yes<br>No<br>Unknown
2c	Was the death non-medically-confirmed, i.e. confirmed by non-medically qualified person, including undertaker, community member, parent, family member, care-giver.	Yes, please specify<br>No<br>Unknown
**C**	**Birth weight of the newborn**	
3	**Please see Reference CRF BIRTH WEIGHT**	
4	If birthweight was ≤2500g, was there documented intra-uterine growth retardation?	Yes<br>No<br>Unknown
[redacted]		
**PART 2 - GESTATIONAL AGE AT BIRTH**		
5	**Please see Reference CRF GA**	
6	Was the gestational age reported by a parent/family member/delivery attendant? *If* ***'no'*** *or* ***'unknown'*** *end of questionnaire*	Yes<br>No<br>Unknown
7	What was the gestational age reported by a parent/family member/delivery attendant?	*Please enter weeks and/or days (e.g. 20 weeks and 2 days)*

Neonatal invasive bloodstream infection

	**NEONATAL INVASIVE BLOODSTREAM INFECTION**<br><br>Neonatal invasive bloodstream infection occurs within the first 28 days of life.	
	**Newborn case number**	
[redacted]		
	**QUESTION**	**ANSWERS**
**A**	**Birth**	
1	Date of birth of the newborn	*Please enter DDMMYYYY or unknown.*
**B**	**Date of onset and diagnosis**	
2	What was the date of the first sign/symptom of the neonatal bloodstream infection?	*Please enter DDMMYYYY or unknown.*
3	What was the date of diagnosis?	*Please enter DDMMYYYY or unknown.*
**C**	**Sample**	
4a	Were any samples from a normally sterile site tested for the presence of bacteria? *Sterile sites are: blood, sterile urine (catheter urine or supra-pubic aspirate), pleural fluid, ascitic*	Yes<br>No<br>Unknown

	fluid, broncho-alveolar lavage, bone biopsy, synovial fluid.<br>(If **'no'** or **'unknown',** skip to section D)	
4b	Was a pathogen found to be present in at least one of the samples? (If **'no'** or **'unknown',** skip to section D)	Yes<br>No<br>Unknown
	Please fill in the question below for the first sample in which a pathogen was found.	
5a	What was the sample?	Ascitic fluid<br>Blood<br>Bone biopsy<br>Broncho-alveolar lavage<br>Pleural fluid<br>Sterile urine (catheter urine or supra-pubic aspirate)<br>Synovial fluid<br>Unknown
5b	Date sample was taken	
5c	What method of identification was used?	Culture<br>Microscopy<br>Molecular methods<br>Serology<br>Other, please specify<br>Unknown
	Is the pathogen present in the sample a bacterium, fungus, protozoa or a virus?	Bacteria<br>Fungi<br>Protozoa<br>Virus
5d	Please specify the name of the pathogen that was present in the sample	*List of bacteria, fungi, protozoa and viruses provided*
	**OPTION TO 'ADD PATHOGEN'**	
	**Immediate automated classification of the organism as pathogenic or non-pathogenic**	
	**OPTION TO 'ADD POSITIVE SAMPLE' AND REPEAT THE QUESTIONS ABOVE**	
**D**	**Criteria**	
	Which of the following signs and symptoms were present at time of hospital admission or diagnosis?	
6a	Temperature ≥37.5◦C or <35.5◦C	Yes<br>No<br>Unknown or not measured
6b	Tachycardia *(heart rate >180 beats per minute)*	Yes<br>No<br>Unknown or not measured

6c	New or more frequent episodes of bradycardia *(heart rate <100 beats per minute)*	Yes<br>No<br>Unknown or not measured
6d	New or more frequent episodes of apnea *(pause in breathing >20 seconds)*	Yes<br>No<br>Unknown or not measured
6e	Increased oxygen requirement	Yes<br>No<br>Unknown
6f	Increased requirement for ventilatory support	Yes<br>No<br>Unknown
6g	Lethargy	Yes<br>No<br>Unknown
6h	Moving only when stimulated	Yes<br>No<br>Unknown
6i	Hypotonia	Yes<br>No<br>Unknown
6j	Irritability	Yes<br>No<br>Unknown
6k	Difficulty in feeding	Yes<br>No<br>Unknown
6l	Abdominal distention	Yes<br>No<br>Unknown
6m	Pallor	Yes<br>No<br>Unknown
6n	Poor perfusion	Yes<br>No<br>Unknown
6o	Hypotension	Yes<br>No<br>Unknown or not measured
6p	Abnormal White Cell Count or I/T ratio >0.2	Yes<br>No<br>Unknown or not measured
6q	Abnormal platelet count *(Low Platelets/Thrombocytopenia: <100,000 × 10^9/L)*	Yes<br>No<br>Unknown or not measured
6r	Increased inflammatory markers (CRP, procalcitonin) *(CRP or calcitonin levels above the local normal standards)*	Yes<br>No

		Unknown or not measured
6s	Metabolic acidosis as defined by a base excess (<−10 mmol/L (−10 mEq/L))	Yes<br>No<br>Unknown or not measured
6t	Tachypnea *(respiratory rate >60 breaths per minute)*	Yes<br>No<br>Unknown or not measured
6u	Severe chest indrawing	Yes<br>No<br>Unknown
6v	Grunting	Yes<br>No<br>Unknown
6w	Cyanosis	Yes<br>No<br>Unknown
6x	Change in level of activity	Yes<br>No<br>Unknown
6y	History of convulsions	Yes<br>No<br>Unknown

Neonatal respiratory infection

NEONATAL RESPIRATORY INFECTION

Neonatal respiratory infection occurs within the first 28 days of life.

	**Newborn case number**<br>**QUESTION**	**ANSWERS**
**A**	**Birth**	
1	Date of birth of the newborn	*Please enter DDMMYYYY or unknown.*
**B**	**Date of onset and diagnosis**	
2	What was the date of the first sign/symptom of the neonatal respiratory infection?	*Please enter DDMMYYYY or unknown.*
3	What was the date of diagnosis?	*Please enter DDMMYYYY or unknown.*
**C**	**Chest X-ray**	
4	Was a chest X-ray performed? *If 'no' or 'unknown' skip to section D.*	Yes<br>No<br>Unknown
5	Which of the following was seen on the chest X-ray?	New or progressive or persistent infiltrate

		Shadowing<br>Fluid in the intrapleural cavity or interlobal fissure<br>None of the above<br>Unknown
**D**	**Upper respiratory sample**	
6	Was an upper respiratory sample tested for the presence of viruses?	Yes<br>No<br>Unknown
7a	Was a virus found to be present in at least one of the samples?	Yes<br>No<br>Unknown
	Please fill in the question below for the first sample in which a virus was found.	
7b	Date sample was taken	*Please enter DDMMYYYY or unknown.*
7c	What method of identification was used?	Culture<br>Microscopy<br>Molecular methods<br>Serology<br>Other, please specify<br>Unknown
7d	Please specify the name of the virus that was present in the sample (more than one answer possible)	*List of viruses provided*
**D**	**Sample from normally sterile site**	
8	Were samples from a normally sterile site tested for the presence of pathogens? *Sterile sites are: blood, sterile urine (catheter urine or supra-pubic aspirate), pleural fluid, ascitic fluid, broncho-alveolar lavage, bone biopsy, synovial fluid. (If* ***'no'*** *or* ***'unknown',*** *skip to section E)*	Yes<br>No<br>Unknown
9a	Was a pathogen found to be present in at least one of the samples? (If **'no'** or **'unknown',** skip to section E)	Yes<br>No<br>Unknown
9b	*Please fill in the question below for the first sample in which a pathogen was found.*	
9c	What was the sample?	Ascitic fluid<br>Blood<br>Bone biopsy<br>Broncho-alveolar lavage<br>Pleural fluid<br>Sterile urine (catheter urine or supra-pubic aspirate)<br>Synovial fluid<br>Unknown
9d	Date sample was taken	*Please enter DDMMYYYY or unknown.*

9e	What method of identification was used?	Culture<br>Microscopy<br>Molecular methods<br>Serology<br>Other, please specify<br>Unknown
9f	Is the pathogen present in the sample a bacterium, fungus, protozoa or a virus?	Bacteria<br>Fungi<br>Protozoa<br>Virus
9g	Please specify the name of the pathogen that was present in the sample (more than one answer possible)	*List of bacteria, fungi, protozoa and viruses provided*
**E**	**Criteria**	
10a	Which of the following signs and symptoms were present at time of hospital admission or diagnosis?	
10b	Temperature ≥37.5◦C or <35.5◦C	Yes<br>No<br>Unknown or not measured
10c	Tachypnea *(respiratory rate >60 breaths per minute)*	Yes<br>No<br>Unknown or not measured
10d	Difficulty in breathing	Yes<br>No<br>Unknown
10e	Nasal flaring	Yes<br>No<br>Unknown
10f	Chest indrawing	Yes<br>No<br>Unknown
10g	Severe chest indrawing	Yes<br>No<br>Unknown
10h	Grunting	Yes<br>No<br>Unknown
10i	Desaturations or increased oxygen requirements or increased ventilator requirements or oxygen saturation <95%	Yes<br>No<br>Unknown
10j	Apneas *(pause in breathing >20 seconds)*	Yes<br>No<br>Unknown
10k	Increased respiratory secretions or Increased suctioning requirements	Yes<br>No<br>Unknown
10l	Cough	Yes

		No<br>Unknown
10m	Wheezing	Yes<br>No<br>Unknown
10n	Crepitations	Yes<br>No<br>Unknown
10o	Increased CRP or procalcitonin *(CRP or calcitonin levels above the local normal standards)*	Yes<br>No<br>Unknown or not measured
10p	Stridor	Yes<br>No<br>Unknown
10q	Fever	Yes<br>No<br>Unknown or not measured

Neonatal meningitis

NEONATAL MENINGITIS

Neonatal meningitis occurs within the first 28 days of life.

	Newborn case number<br>QUESTION	ANSWERS
**A**	**Birth**	
1	Date of birth of the newborn	*Please enter DDMMYYYY or unknown.*
**B**	**Date of onset and diagnosis**	
2	What was the date of the first sign/symptom of neonatal meningitis?	*Please enter DDMMYYYY or unknown.*
3	What was the date of diagnosis?	*Please enter DDMMYYYY or unknown.*
**C**	**Cerebrospinal fluid sample**	
4	Was an cerebrospinal fluid (CSF) sample tested for the presence of pathogens?	Yes<br>No<br>Unknown
5a	Was a pathogen found to be present in at least one of the samples?	Yes<br>No<br>Unknown
	Please fill in the question below for the first sample in which a pathogen was found.	

5b	Date sample was taken	*Please enter DDMMYYYY or unknown.*
5c	What method of identification was used?	Culture<br>Microscopy<br>Molecular methods<br>Serology<br>Other, please specify<br>Unknown
5d	Is the pathogen present in the sample a bacterium, fungus, protozoa or a virus?	Bacteria<br>Fungi<br>Protozoa<br>Virus
5e	Please specify the name of the pathogen that was present in the sample (more than one answer possible)	*List of bacteria, fungi, protozoa and viruses provided*
**D**	**Sample from normally sterile site**	
6	Was a samples from a normally sterile sites (other than CSF) tested for the presence of pathogens? *Sterile sites are: blood, sterile urine (catheter urine or supra-pubic aspirate), pleural fluid, ascitic fluid, broncho-alveolar lavage, bone biopsy, synovial fluid.*<br>*(If **'no'** or **'unknown',** skip to section E)*	Yes<br>No<br>Unknown
7a	Was a pathogen found to be present in at least one of the samples?<br>*(If **'no'** or **'unknown',** skip to section E)*	Yes<br>No<br>Unknown
	Please fill in the question below for the <u>first sample</u> in which a pathogen was found.	
7b	What was the sample?	Ascitic fluid<br>Blood<br>Bone biopsy<br>Broncho-alveolar lavage<br>Pleural fluid<br>Sterile urine (catheter urine or supra-pubic aspirate)<br>Synovial fluid<br>Unknown
7c	Date sample was taken	*Please enter DDMMYYYY or unknown.*
7d	What method of identification was used?	Culture<br>Microscopy<br>Molecular methods<br>Serology<br>Other, please specify<br>Unknown

7e	Is the pathogen present in the sample a bacterium, fungus, protozoa or a virus?	Bacteria<br>Fungi<br>Protozoa<br>Virus
7f	Please specify the name of the pathogen that was present in the sample (more than one answer possible)	*List of bacteria, fungi, protozoa and viruses provided*
**E**	**Signs and symptoms**	
8a	Which of the following signs and symptoms were present at time of hospital admission or diagnosis?	
8b	CSF pleocytosis *(CSF pleocytosis: ≥20 cells/mm^3 for <28 day-olds and ≥10 cells/mm^3 for 29–89 day-olds. # i–89 day-olds)*	Yes<br>No<br>Unknown or not measured
8c	Positive IgM antibodies to a specific pathogen in CSF	Yes<br>No<br>Unknown or not measured
8d	Temperature ≥37.5◦C or <35.5◦C	Yes<br>No<br>Unknown or not measured
8e	History of convulsions	Yes<br>No<br>Unknown
8f	Lethargy or irritability	Yes<br>No<br>Unknown
8g	Coma	Yes<br>No<br>Unknown
8h	Apnea (pause in breathing >20 seconds)	Yes<br>No<br>Unknown
8i	Bulging fontanel	Yes<br>No<br>Unknown
8j	Neck stiffness	Yes<br>No<br>Unknown

Stillbirth

**STILLBIRTH**

Stillbirth is defined as a fetal death occuring before birth after a selected, predefined duration of gestation. The death of the fetus could have occurred before the onset of labor (antepartum) or at the time delivery (intrapartum).

	**Newborn case number**<br>**QUESTION**	**ANSWERS**
**PART 1 - STILLBIRTH**		
**A**	**Birth**	
1	What was the date of delivery?<br>*(If **'no'** or **'unknown'** skip to section C)*	*Please enter DDMMYYYY or unknown.*
2	Was the delivery attended?<br>*(If 'no' or 'unknown' skip to section C)*	Yes<br>No<br>Unknown
3	Who attended the delivery?	Midwife<br>Obstetrician<br>Matern-fetal medicine specialist<br>Other, please specify<br>Unknown
**C**	**Absence of signs of life at birth**	
4a	Was a physical examination of the fetus/infant performed after delivery?<br>*(If **'no'** or **'unknown'** skip to section D)*	Yes<br>No<br>Unknown
4b	By whom was the physical examination performed?	General practice physician<br>Matern-fetal medicine specialist<br>Midwife<br>Neonatalogist<br>Nurse practitioner<br>Obstetrician<br>Pathologist<br>Pediatrician<br>Physician's assistant<br>Other qualified trained practitioner, please specify<br>Other, please specify<br>Unknown
5	Indicate among the following signs, which were present at birth	
5a	Sign of life (unspecified)	Yes<br>No<br>Unknown
5b	Spontaneous movement	Yes<br>No<br>Unknown

5c	Umbilical cord pulse	Yes<br>No<br>Unknown
5d	Heartbeat	Yes<br>No<br>Unknown
5e	Respiration/spontaneous respiration	Yes<br>No<br>Unknown
5f	Crying	Yes<br>No<br>Unknown
6	Was the Apgar score 0 at 1 and 5 minutes?	Yes<br>No<br>Unknown
7	Was there whole body cyanosis?	Yes<br>No<br>Unknown
8	Was there response to resuscitation?	Yes<br>No<br>Unknown
9a	Was the physical examination after birth consistent with antepartum death? (e.g. maceration, meconium staining, tissue injury, oedema)	Yes<br>No<br>Unknown
9b	Was the physical examination after birth consistent with intrapartum death?	Yes<br>No<br>Unknown
**E**	**Pathology report**	
10	Was a fetal/placental pathology report available?<br>*(If* ***'no'*** *or* ***'unknown'*** *skip to section F)*	Yes<br>No<br>Unknown
11	Was this consistent with antepartum death?	Yes<br>No<br>Unknown
**F**	**Presence of fetal heart tones and fetal movement prior to the onset of labor**	
12a	Was there a prenatal ultrasound examination ?<br>*(If* ***'no'*** *or* ***'unknown'*** *skip to question 13a)*	Yes<br>No<br>Unknown
12b	Did the prenatal ultrasound examination document fetal heart tones before the onset of labor?	Yes<br>No<br>Unknown
12c	Did the prenatal ultrasound examination document fetal movement before the onset of labor?	Yes<br>No<br>Unknown
13a	Was auscultation for fetal heart tones performed prior to the onset of labor?	Yes<br>No

	(If ***'no'*** *or* ***'unknown'*** *skip to question 14a)*	Unknown
13b	Were fetal heart tones documented by auscultation prior to the onset of labor?	Yes<br>No<br>Unknown
14a	Was a hand held Doppler used for fetal heart tones performed prior to the onset of labor?<br>*(If* ***'no'*** *or* ***'unknown'*** *skip to question 15a)*	Yes<br>No<br>Unknown
14b	Were fetal heart tones document by a hand held Doppler prior to the onset of labor?	Yes<br>No<br>Unknown
15a	Does the medical record state the mother reported fetal movement prior to onset of labor?	Yes<br>No<br>Unknown
15b	Does the medical record state the mother reported a lack of fetal movement for 24 hours of more prior to the onset of labor?	Yes<br>No<br>Unknown
15c	Did a physical examination of the mother confirm lack of fetal movement?	Yes<br>No<br>Unknown
16	Are radiology findings consistent with intrauterine fetal death?	Yes<br>No<br>Unknown<br>Not applicable
**PART 2 - GESTATIONAL AGE AT STILLBIRTH**		
**17**	**Please see Reference CRF GA**	

Low birth weight

	**LOW BIRTH WEIGHT**
	Low birth weight is defined as <2500 grams
**PART 1 - GESTATIONAL AGE AT BIRTH**	
1	**Please see Reference CRF BIRTH WEIGHT**

Small for gestational age

	**SMALL FOR GESTATIONAL AGE**

	Small for gestational age is defined as a birth weight for gestational age below the 10th percentile based on a sex-specific reference population (WHO, 1995)	
	**Newborn case number**<br>**QUESTION**	**ANSWERS**
**PART 1 - GA**		
1	**Please see Reference CRF GA**	
**PART 2 - BIRTH WEIGHT**<br>**H**	 **Birth weight of the newborn**	
2	**Please see Reference CRF birth weight**	
**PART 3 - SGA**		
3	Was GA assessed only by infant examination?	Yes<br>No<br>Unknown
4	Was the baby noted to be small, but no actual weight recorded?	Yes<br>No<br>Unknown
5	Was the diagnosis of small for gestational age extracted from billing codes or chart, with no documentation of actual birth weight or GA?	Yes<br>No<br>Unknown

Congenital microcephaly (post-natally diagnosed)

	**POST NATALLY DIAGNOSED CONGENITAL MICROCEPHALLY**	
	Small for gestational age is defined as a birth weight for gestational age below the 10th percentile based on a sex-specific reference population (WHO, 1995)	
	**Newborn case number**<br>**QUESTION**	**INSTRUCTIONS**
**PART 1 - GA and sex**		
1	Did a live birth, stillbirth, or spontaneous/therapeutic abortion take place?	Live birth<br>Stillbirth<br>Spontaneous abortion<br>Therapeutic abortion<br>Unknown
2	What is the sex of the baby?	Male

		Female<br>Unknown
3	**Please see Reference CRF GA**	
**PART 2 - Congenital microcephaly**		
1	Was the head circumference measured and recorded?<br>*If 'no' or 'unknown' skip question 5.*	Yes<br>No<br>Unknown
2a	What was the head circumference?	*Please enter a number.*
2b	What are the units of the head circumference measurement?	Centimeters<br>Inches<br>Other, please specify<br>Unknown
4	When was the head circumference measured?	Within the first 24h (take into account the variability in this period based on molding of the head)<br>24-36h after birth or end of pregnancy<br>>36h and up to 6 weeks after birth or end of pregnancy with no apparent post-natal insult resulting in microcephaly
5a	Was there any diagnosis of congenital microcephaly using using codes ICD-9-CM code 742.1 or ICD-10-CM code Q02?<br>*If 'no' or 'unknown' skip to last question.*	Yes<br>No<br>Unknown
5b	Was there an inpatient diagnosis of congenital microcephally using ICD-9-CM code 742.1 or ICD-10-CM code Q02?	Yes<br>No<br>Unknown
5c	Were there at least 2 outpatient diagnosis of congenital microcephally using ICD-9-CM code 742.1 or ICD-10-CM code Q02?	Yes<br>No<br>Unknown
5d	Was there an outpatient diagnosis of congenital microcephally AND death in the first year using ICD-9-CM code 742.1 or ICD-10-CM code Q02?	Yes<br>No<br>Unknown
6	Was congenital microcephaly based on physical inspection without head circumference measurement?	Yes<br>No<br>Unknown

Reference CRF: gestational age

	QUESTION	ANSWERS
**GESTATIONAL AGE**		

**A**	**Birth**	
1	Date of birth of the newborn	*Please enter DDMMYYYY or unknown.*
**B**	**Last menstrual period (LMP) of the pregnant woman**	
3a	What is the date of LMP ?<br>*(if **'unknown'**, skip to section C)*	*Please enter DDMMYYYY or unknown.*
**C**	**Assisted reproduction technology**	
4	Was the conception natural (i.e. no assisted reproduction technology was used)?<br>*(if **'yes'** or **'unknown'**, skip to section D)*	Yes<br>No<br>Unknown
	<u>Intrauterine insemination</u><br>*Intrauterine insemination is a procedure in which a fine catheter is inserted through the cervix into the uterus to deposit a sperm sample directly into the uterus, to achieve fertilisation and pregnancy*	
5a	Did intrauterine insemination take place for this pregnancy?<br>(if **'no'** or **'unknown',** skip to section D)	Yes<br>No<br>Unknown
5b	What was the date of intrauterine insemination?	*Please enter DDMMYYYY or unknown.*
	<u>Embryo transfer</u><br>*Embryo transfer is the procedure in which one or more embryos are placed in the uterus or fallopian tube.*	
6a	Did embryo transfer take place for this pregnancy?<br>(if **'no'** or **'unknown',** skip to section D)	Yes<br>No<br>Unknown
6b	What was the date of embryo transfer?	*Please enter DDMMYYYY or unknown.*
**D**	**Ultrasound during pregnancy**	
7	Was an ultrasound done during the pregnancy?<br>*(if **'no'** or **'unknown'**, skip to section E)*	Yes<br>No<br>Unknown
	<u>Ultrasound scan Trimester 1</u>	
8a	Was an ultrasound done during the first trimester? *First trimester is defined as "≤13 weeks + 6/7 days".*<br>*(if **'no'** or **'unknown'**, skip to question 9a)*	Yes<br>No<br>Unknown
8b	What was the date of the ultrasound scan? *In case >1 ultrasound was done during the first trimester, please use the one closest to 9 week of gestation.*	*Please enter DDMMYYYY or unknown.*
8c	What was the estimated gestational age based on the ultrasound scan?	*Please enter number of weeks and/or days (e.g. 25 weeks and 2 days)*

8d	What was the expected delivery date based on the ultrasound scan?<br>*(if questions **8b** AND **8c** are known, or question **8d** is known, **end of questionnaire.** Else continue with question **9a**)*	*Please enter DDMMYYYY or unknown.*
	Ultrasound scan Trimester 2	
9a	Was an ultrasound done during the second trimester?<br>*Second trimester is defined as "14 weeks + 0 days to 27 week + 6 days"*<br>*(if 'no' or 'unknown', skip to question 10a)*	Yes<br>No<br>Unknown
9b	What was the date of the ultrasound scan	*Please enter DDMMYYYY or unknown.*
9c	What was the estimated gestational age based on the ultrasound scan?	*Please enter number of weeks and/or days (e.g. 25 weeks and 2 days)*
9d	What was the expected delivery date based on the ultrasound scan?<br>*(if questions **9b** AND **9c** are known, or question **9d** is known, **end of questionnaire**. Else continue with question **10a**)*	*Please enter DDMMYYYY or unknown.*
	Ultrasound scan Trimester 3	
10a	Was an ultrasound done during the third trimester? *Third trimester is defined as "≥28 weeks"*	Yes<br>No<br>Unknown
10b	What was the date of the ultrasound scan	*Please enter DDMMYYYY or unknown.*
10c	What was the estimated gestational age based on the ultrasound scan?	*Please enter number of weeks and/or days (e.g. 25 weeks and 2 days)*
10d	What was the expected delivery date based on the ultrasound scan?	*Please enter DDMMYYYY or unknown.*
**E**	**Physical examination of pregnant woman in first trimester** *First trimester is defined as "≤13 weeks + 6/7 days"*	
11a	Was a physical examination of the mother done in the first trimester?<br>(if '**no**' or '**unknown**', skip to section F)	Yes<br>No<br>Unknown
11b	What was the date of the examination?	*Please enter DDMMYYYY or unknown.*
11c	If yes, did a pelvic bimanual examination confirm an enlarged uterus?<br>(if '**no**' or '**unknown**', skip to section F)	Yes<br>No<br>Unknown
11d	What was the estimated gestational age on the physical examination?	*Please enter number of weeks and/or days (e.g. 25 weeks and 2 days)*
11e	What was the expected delivery date based on the physical examination?	*Please enter DDMMYYYY or unknown.*

**F**	**Fundal height of pregnant woman in second trimester**<br>*Second trimester is defined as "14 weeks + 0 days to 27 week + 6 days"*	
12a	Was the fundal height measured in the 2nd trimester? *In case >1 fundal height measurement was done during the 2nd trimester, please report the first one.*<br>(if '**no**' or '**unknown**', skip to section G)	Yes<br>No<br>Unknown
12b	What was the fundal height?	*Please enter a number.*
12c	What is the measurement unit of the fundal height?	Centimeters<br>Inches<br>Other, please specify<br>Unknown
12d	What is the date of fundal height measurement?	*Please enter DDMMYYYY or unknown.*
**G**	**Birth weight of the newborn**	
13a	Was the newborn weighed at birth?<br>(if '**no**' or '**unknown**', skip to section H)	Yes<br>No<br>Unknown
13b	What are the units of the birth weight measurement?	Grams<br>Kilograms<br>Pounds<br>Other, please specify<br>Unknown
13c	What was the birth weight?	*Please enter a number.*
13d	What was the date of birthweight measurement?	*Please enter DDMMYYYY or unknown.*
H	**New Ballard Score of the newborn**	
14a	Was a New Ballard Score calculated for the newborn?<br>(if '**no**' or '**unknown**',end of questionnaire)	Yes<br>No<br>Unknown
14b	What was the neuromuscular maturity score?	*Please enter a number*
14c	What was the physical maturity score?	*Please enter a number*
14d	What is the total score?	*Please enter a number*
14e	What is the date of scoring?	*Please enter DDMMYYYY or unknown.*

Reference CRF: birth weight

	QUESTION	ANSWERS
**PART 1 – BIRTH WEIGHT**		
**A**	**Birth**	
1	Date of birth of the newborn	*Please enter DDMMYYYY or unknown.*
**B**	**Weight**	

1	Was the newborn weighed?<br>*If ' no' or ' unknown' skip to B4..*	Yes<br>No<br>Unknown
2a	What was the weight of the newborn infant?<br>*If 'unknown' skip to question 3.*	*Please enter a number.*
2b	What are the units of the weight measurement?	Grams<br>Kilograms<br>Pounds<br>Other, please specify<br>Unknown
3	Was the weight category assessed as <2500g?	Yes<br>No<br>Unknown
4	Was a proxy measure of birth weight used?	Yes, please specify<br>No<br>Unknown
5	When was the newborn infant weighed?	Within 24 hours of birth<br>On day 1 or 2 of life (first 48 hours)<br>On day 3 or after<br>Unknown
	**Scale**	
1	What type of scale was used?	Electronic scale<br>Spring scale<br>Dial<br>Color-coded<br>Unknown
2	How is the scale graduated?	To 10 grams<br>To at least 50 grams<br>Other<br>Unknown
3	How often is the scale calibrated?	At least once a year<br>At least once a year, or more often if moved<br>Other<br>Unknown
4	What is the scale tared to?	0 grams<br>0.00 kg<br>Other<br>Unknown
5	(For electronic scales): Is the scale placed on a level, hard surface?	Yes<br>No<br>Unknown

6	Is the infant weighed by measuring the difference between an adult holding the infant and the adult being weighed alone?	Yes<br>No<br>Unknown
	**Standard operating procedures (to be completed only once per site)**	
1	*Does the health center's standard operating procedure fulfill the following criteria?*	
	1. Newborn infant weighed within 24 hours of birth AND<br>2. Use electronic scale which is graduated to 10 grams AND<br>3. Scale is calibrated at least once a year AND<br>4. Scale placed on level, hard surface AND<br>5. Scale tared to zero grams	Yes<br>No<br>Unknown
	1. Newborn infant weighed within 24 hours of birth AND<br>2. Scale (electronic/ spring) is graduated to at least 50 grams AND<br>3. Scale is calibrated at least once a year, or more often if moved AND<br>4. Scale tared to zero grams or 0.00kg	Yes<br>No<br>Unknown

Maternal immunization

QUESTION		ANSWERS
1	What is the date of LMP ?<br>(if **'unknown'**, skip all questions on 30 days before LMP)	*Please enter DDMMYYYY or unknown.*
2a	Did the mother receive any vaccination during pregnancy? *Please only answer 'no' if there is documented evidence of no immunization; else please answer 'unknown'.*	Yes<br>No<br>Unknown
2b	Did the mother receive any vaccination in the 30 days before LMP? *Please only answer 'no' if there is documented evidence of no immunization; else please answer 'unknown'.*	Yes<br>No<br>Unknown
	(If the answer to both question above is 'no' or 'unknown', end of questionnaire.)	
3	How many vaccine doses were administered during pregnancy in total?	1<br>2<br>3<br>4<br>>4<br>Unknown
	Fill in the questions below for each dose administered during pregnancy	

	**Dose during pregnancy**	
4	Against which disease was the mother immunized?	Tetanus<br>Influenza<br>Pertussis<br>Other, please specify
5	Was vaccination status obtained from medical records or reported by the mother?	Medical records (e.g. ANC card, vaccination card, vaccine registry, etc.)<br>Reported by mother but no formal recording<br>Unknown
6	What is the date of immunization of the pregnant woman?	*Please enter DDMMYYYY or unknown.*
7	What is the time of immunization of the pregnant woman?	*Please enter the time or unknown.*
8	Was the date recorded in medical records by health care worker who administered/witnessed administration of vaccine?	Yes<br>No<br>Unknown
9	What is the name of the vaccine?	Adacel<br>Boostrix<br>Daptacel<br>Infanrix<br>Kinrix<br>Pediatrix<br>Pentacel<br>Quadracel<br>Tenivac<br>Other, please specify<br>Unknown
10	What is the manufacturer?	GlaxoSmithKline<br>Sanofi Pasteur<br>Other, please specify<br>Unknown
11	What is the lot number?	*Please enter your answer in free text.*
	**OPTION TO 'ADD DOSE DURING PREGNANCY' AND REPEAT QUESTIONS**	
12	How many vaccine doses were administered in the 30 days before LMP?	*Please select your answer from the list.*<br>Fill in the questions below for each dose administered in the 30 days before LMP
	**Dose 30 days before last LMP**	
	Same questions as above	

OPTION TO 'ADD DOSE 30 days before last LMP' AND REPEAT QUESTIONS

Annex VI. Patient information sheet

You have registered in the antenatal clinic and are going to deliver your baby in this health facility; or

You have delivered your baby in this health facility or

You have been referred to this health facility for delivery.

This health facility is going to take part in a research that will study the information gathered from mothers during pregnancy, delivery and the status of their new born babies in this health facility. This research is going to be conducted in …. health facilities in …. Countries, and the health facilities will be collecting information for one year. In this research, information about your pregnancy and the health status of your new born baby collected in the health facility as part of routine patient care may be used. There will be no new vaccine or drug administered or any change in the health facility practice. So, there will be no direct benefit to you or your child due to this research. But information gathered from several pregnant mothers and their new born babies from the multiple health facilities that take part in this study will be transferred in a safe and confidential way to a database that will be studied by the WHO. This will then support future research and detection and reporting of side effects when new vaccines or new medical intervention are introduced in pregnant women. This will also help in planning steps to improve the health facility data collection and recording practices; in turn benefit future pregnant mothers and babies

All information used for research will not bear your name, address or any other personal information about you or your child that will trace your identity. So, your individual identity will be protected. Health facility will assign a responsible person to use and store the research data in a safe place.

The key-coded data of your child obtained from this study will be stored in a secured database located in India. The study data will be sent to the WHO and any partners working with the WHO for review or scientific analysis. It may also be added to research databases and used in the future by the WHO and other companies and people working for or with the WHO to improve future research and to improve the detection and reporting of side effects when new vaccines or new medical interventions are given in pregnant women. The personal data of your child will always be handled in accordance with all applicable data protection and privacy laws. All information about your child as an individual is confidentially protected and will only be communicated to authorized persons, including the WHO and their representatives and agents. Any information collected from other physicians will be handled in the same confidential manner as those collected by the study doctor.

If you are willing to allow the information collected in the health facility as part of routine patient care about you and your new born baby to be used for this research, please sign and date this form. You are free to contact …….. to understand how your information has been made use of. If at any time you do not wish to share your information, you are free to contact …. and withdraw from this study.

You also have the choice to say no and opt out of this research. By doing so, be rest assured your or your child's care in the health facility will not be affected in any way.

I have read the foregoing information, or it has been read to me. I have had the opportunity to ask questions about it and all questions have been answered to my satisfaction. I consent voluntarily to be a participant in this study

Print Name of Participant____

Signature of Participant

Date ______________________

Day/month/year

<u>If participant is illiterate</u> 4

I have witnessed the accurate reading of the consent form to the potential participant, and the individual has had the opportunity to ask questions. I confirm that the individual has given consent freely.

Print name of witness__________

Signature of witness __________

Date ____________________

Day/month/year

Thumb print of participant

<u>If participant is a minor</u> 5

I am the legally authorised representative (LAR) of the potential participant. I have read the foregoing information, or it has been read to me. I have had the

[4] A literate witness must sign (if possible, this person should be selected by the participant and should have no connection with the research team). Participants who are illiterate should include their thumb print as well.

[5] A minor participant should be supported in the decision making by a Legally authorized representative, as applicable in the country. If the LAR is illiterate then a literate witness should participate in the informed consent process. Participants should sign for themselves

opportunity to ask questions and all questions have been answered to my satisfaction. I hereby give consent on behalf of my ……..

Print name of LAR__________________ **Thumb print of LAR if applicable**

Signature of LAR ____________________

Date ______________________

Day/month/year

Statement by the researcher/person taking consent

I have accurately read out the information sheet to the potential participant. I confirm that the participant was given an opportunity to ask questions about the study, and all the questions asked by the participant have been answered correctly and to the best of my ability. I confirm that the individual has not been coerced into giving consent, and the consent has been given freely and voluntarily.

A copy of this ICF has been provided to the participant.

Print Name of Researcher/person taking the consent______________________

Signature of Researcher /person taking the consent________________________

Date _________________________

Day/month/year
