# Supplementary material for: WHO global vaccine safety multi-country collaboration project on safety in pregnancy: Assessing the level of diagnostic certainty using standardized case definitions for perinatal and neonatal outcomes and maternal immunization
Source: Vaccine X. 2021 Nov 3;9:100123. doi: 10.1016/j.jvacx.2021.100123 (PMC8605263; doi:10.1016/j.jvacx.2021.100123)
Supplement: Supplementary data S3 — Using GAIA definitions [file mmc4.docx]

**S3 – Using GAIA definitions**

**S3a – Summary of GAIA definitions**

Table S3a-1. Summary of the GAIA definitions for the perinatal and neonatal outcomes and maternal immunization. Full definitions are referenced.

| **Perinatal and Neonatal Outcomes**  *Requirements for all levels* | **Criterion** | **Levels of diagnostic certainty** | | |
| --- | --- | --- | --- | --- |
|  |  | **Level 1** | **Level 2** | **Level 3** |
| **GA**^1^ |  | 1st trimester US scan | a) Certain LMP + (2nd trimester scan OR  1^st^ trimester physical exam)  b) Uncertain LMP with 2^nd^ trimester US scan | a) Certain LMP + (3^rd^ trimester US scan OR confirmatory 2^nd^ trimester fundal height OR BW); OR uncertain LMP + 1^st^ trimester physical examination  b) Uncertain LMP + (fundal height OR newborn physical exam OR BW) |
| **LBW**^2^  *<2500g* | Weighted | Within 24h | Wthin 24h | Day 1 or 2 |
|  | Scale (resolution) | Calibrated, electronic (10g) | Calibrated, electronic/spring (50g) | Dial/spring/color-coded |
| **Preterm Birth**^1^  *GA<37 weeks* | GA | Level 1 | Level 2 | Level 3 |
| **SGA**^3^  *Weight <10^th^ for GA* | Weighted | Within 24h | Within 24h | Within 48h |
|  | Calibrated scale (resolution) | Electronic (10g) | Electronic (50g) | Electronic (50g) |
|  | GA | Level 1 | a) Level 2a, b) Level 2b | a) Level 3a, b) Level 3b |
| **Antepartum Stillbirth**^4^  *No signs of life at birth, post birth physical exam* | Fetal death before labor as evidenced through | US OR (auscultation AND maternal report/physician exam) OR radiology OR pathology | Auscultation OR maternal report OR physical exam OR pathology | Maternal report OR auscultation |
|  | Birth attendance | Attended | Attended | Non-attended |
|  | GA | Level 1-2 | Level 1-2 | Level 2-3 |
| **Intrapartum**  **Stillbirth**^4^  *No signs of life at birth, post birth physical exam* | Live fetus before labor as evidenced through | US | Maternal report AND (auscultation OR Doppler) | Maternal report OR auscultation |
|  | Birth attendance | Attended | Attended | Non-attended |
|  | GA | Level 1 | Level 1-2 | Level 2-3 |
| **Neonatal death**^5^  *Live born+ death in first 28 days* | GA/BW | GA Level 1 OR BW available | GA Level 1-2 OR BW available | GA OR GA Level 2-3/reported* OR BW available |
|  | Medical confirmation required | Yes | - | - |
| **Neonatal BSI**^6^  *Infection in first 28 days* | Pathogen | From normally sterile site | - | - |
|  | Clinical/lab criteria | - | 3 clinical/lab criteria | 2 clinical criteria |
| **Neonatal Respiratory** **infection**^6^  *Infection in first 28 days* | Relevant findings on chest X-ray | Yes | Yes |  |
|  | Pathogen | From normally sterile site OR upper respiratory tract | - | - |
|  | Clinical/lab criteria | No | 4 clinical/lab criteria | 2 clinical criteria |
| **Neonatal meningitis**^6^  *Infection in first 28 days* | Pathogen | From CSF | From normally sterile site (other than CSF) | No |
|  | CSF findings required other than pathogen | None | Pleocytosis or positive IgM | 1. Pleocytosis |
|  | Clinical criteria | - | Fever + 1 clinical criterion | 1. Fever + 3 clinical criteria 2. Fever + 4 clinical criteria |
| **Congenital Microcephaly^7^** | GA ≥24 weeks | yes | yes | a) yes  b) - |
|  | GA | Modified Level 1 | a) Modified level 1 or 2,  b) Level 2b | a) Modified level 3  b) - |
|  | HC 2SD below mean <3 percentile according to GA and gender | Yes | a-b) Yes | a) Yes, b) - |
|  | HC measurement | Between 24 and 36h of birth/end of pregnancy | a-b) Measured within 24h or between 36h and 6 weeks of birth/end of pregnancy | a) Up to 6 weeks of birth/end of pregnancy |
|  | ICD | - | - | b) validated algorithm using ICD codes |
| **Maternal immunization**^5^ | Date/time | Date and time | Month and year | - |
|  | Source | Primary | Primary | Non-primary |
|  | Details required | Details of vaccine, incl. lot number | Details of disease against which vaccinated | Report of receipt of vaccination during pregnancy |

*BW: birth weight; CSF: cerebrospinal fluid; GA:Gestational Age; HC; head circumference; LBW: Low BirthWeight; SGA: Small for Gestational Age; US: Ultrasound; LMP: Last Menstrual Period; ICD: International Classification of Diseases.*

^1^ Quinn J-A, Munoz FM, Gonik B, Frau L, Cutland C, Mallett-Moore T, et al. Preterm birth: Case definition & guidelines for data collection, analysis, and presentation of immunisation safety data. Vaccine. 2016;34:6047-56. DOI: [10.1016/j.vaccine.2016.03.045](https://doi.org/10.1016/j.vaccine.2016.03.045)

^2^ Cutland C, Lackritz E, Mallett-Moore T, Bardaji A, Chandrasekaran R, Lahariya C, et al. Low birth weight: Case definition & guidelines for data collection, analysis, and presentation of maternal immunization safety data. Vaccine. 2017;35:6492-500. DOI: [10.1016/j.vaccine.2017.01.049](https://doi.org/10.1016/j.vaccine.2017.01.049)

^3^ Schlaudecker EP, Munoz FM, Bardají A, Boghossian NS, Khalil A, Mousa H, et al. Small for gestational age: Case definition & guidelines for data collection, analysis, and presentation of maternal immunisation safety data. Vaccine. 2017;35:6518-28. [10.1016/j.vaccine.2017.01.040](https://doi.org/10.1016/j.vaccine.2017.01.040)

^4^ Da Silva FT, Gonik B, McMillan M, Keech C, Dellicour S, Bhange S, et al. Stillbirth: Case definition and guidelines for data collection, analysis, and presentation of maternal immunization safety data. Vaccine. 2016;34:6057-68. [10.1016/j.vaccine.2016.03.044](https://doi.org/10.1016/j.vaccine.2016.03.044)

^5^ Pathirana J, Muñoz FM, Abbing-Karahagopian V, Bhat N, Harris T, Kapoor A, et al. Neonatal death: Case definition & guidelines for data collection, analysis, and presentation of immunization safety data. Vaccine. 2016;34:6027-37. [10.1016/j.vaccine.2016.03.040](https://doi.org/10.1016/j.vaccine.2016.03.040)

^6^ Vergnano S, Buttery J, Cailes B, Chandrasekaran R, Chiappini E, Clark E, et al. Neonatal infections: Case definition and guidelines for data collection, analysis, and presentation of immunisation safety data. Vaccine. 2016;34:6038-46. [10.1016/j.vaccine.2016.03.046](https://doi.org/10.1016/j.vaccine.2016.03.046)

^7^ Bonhoeffer J, DeSilva M, Muñoz F, Sell E, Marshall H, Tse Kawai A, et al. Congenital microcephaly: Case definition & guidelines for data collection, analysis, and presentation of safety data after maternal immunisation. Vaccine. 2017;35:6472-82. [10.1016/j.vaccine.2017.01.044](https://doi.org/10.1016/j.vaccine.2017.01.044)

**S3b: Higher levels of evidence used at lower levels of certainty**

Low birth weight

Level 3 (scale type)

In addition to the spring being of the types mentioned in level 3 (i.e. dial/spring/color-coded), also electronic scales (sufficient for levels 1 and 2) were considered appropriate for a level 3 classification.

Small for gestational age

Level 3B (method of weight measurement)

In addition to an infant being weighted within the first 48h of life with the newborn weight assessed by measuring the difference between an adult holding the infant and the adult being weighed alone on any scale. Also weight measured within 48h of birth using any scale with a <50 g resolution, tared to zero and calibrated (level 3A requirement) was allowed.

Stillbirth

Level 3 (gestational age classification)

The original level 3 definition requires a gestational age with a level of confidence of 2 or 3. In addition to this also a gestational age with a level of confidence of 1 was permitted (this was sufficient for the level 1 and 2 classifications for stillbirth).

Level 3 (Antepartum: attendance of the delivery)

The level 3 definition requires either of

- Non-attended delivery followed by physical examination of the fetus after birth consistent with antepartum death by a healthcare professional appropriate to the level of standard of care in the health care setting.
- Verbal history by a trained health care provider, non-medical witness or the mother of a fetus born with no signs of life or unresponsive to resuscitation efforts immediately after birth and with physical features consistent with antepartum death.

In the implementation, this was changed to allow information sufficient for a level 1 or 2 classification and one of the following was required for a level 3 classification

- Non-attended delivery followed by physical examination of the fetus after birth consistent with antepartum death by a healthcare professional appropriate to the level of standard of care in the health care setting.
- Verbal history by a trained health care provider, non-medical witness or the mother of a fetus born with no signs of life or unresponsive to resuscitation efforts immediately after birth and with physical features consistent with antepartum death.
- Attended delivery followed by physical examination after birth consistent with antepartum death, by specialist or qualified trained practitioner appropriate to the health care setting.
- Fetal/placental pathology report consistent with antepartum death.

Level 3 (Intrapartum: attendance of the delivery)

The level 3 definition requires either of

- Non-attended delivery followed by physical examination of the fetus after birth consistent with intrapartum death by a healthcare professional appropriate to the level of standard of care in the health care setting
- verbal history by a trained health care provider, non-medical witness or the mother of a fetus born with no signs of life or unresponsive to resuscitation efforts immediately after birth.

In the implementation, this was changed to allow information sufficient for a level 1 or 2 classification and one of the following was required for a level 3 classification

- intrapartum death by a healthcare professional appropriate to the level of standard of care in the health care setting
- verbal history by a trained health care provider, non-medical witness or the mother of a fetus born with no signs of life or unresponsive to resuscitation efforts immediately after birth.
- Attended delivery followed by physical examination after birth consistent with intrapartum death by a health care professional appropriate to the level of standard of care in the health care setting.

Level 2 and 3 (Antepartum: report of lack of fetal heartbeat)

One of the sufficient conditions used for level 2 and 3 is a report of auscultation for fetal heart tones (using electronic or non-electronic devices) documenting lack of fetal heartbeat. In level one a prenatal ultrasound examination documenting lack of fetal cardiac activity or movement before the onset of labor is also allowed. In the implementation of level 2 and 3 definitions when at least one of

- a report of auscultation for fetal heart tones (using electronic or non-electronic devices) documenting lack of fetal heartbeat
- prenatal ultrasound examination documenting lack of fetal cardiac activity or movement before the onset of labor

was considered sufficient to document a lack of fetal heartbeat

Level 2 and 3 (Intrapartum report of fetal heartbeat)

Similar to the antepartum definition also here US information on the presence of a fetal heartbeat was considered to provide valid information on the presence of a fetal heartbeat.

Neonatal meningitis

Level 3b

For level 3b, the availability of a sample in which no pathogen was identified was allowed.

Maternal immunization

Level 3

The level 3 classification requires that the woman reports receipt of vaccination during pregnancy, but no formal recording of immunization available. In the implementation also other sources of information on the vaccination (recorded in medical records by health care worker who administered / witnessed administration of vaccine) were allowed for a level 3 classification.

**S3c: Lessons from field-testing the GAIA definitions (continued)**

SGA definition: SGA assessment with GA based on BW

One of the options to determine GA in SGA level 3b is “uncertain LMP with birthweight”, essentially requiring only birth weight. This leads to the situation where both criteria of SGA assessment (birth weight and GA) are based on the same parameter, which cannot lead to a diagnosis of SGA. Therefore, we suggest removal of the possibility to assess GA based on birth weight in the SGA case definition.

Neonatal death definition: Viability/maturity in neonatal deaths

In the neonatal death case definition, neonates are categorized by degree of viability and maturity (e.g. non-viable or extremely preterm live birth). The categorization is based on predefined ranges of GA or BW. In this study, categories of viability and maturity were not studied, however, we assessed whether the information required for the categorization was present. Both GA and BW were available for most subjects, and in several instances, the maturity category in which the subject would be placed differed based on whether the GA or the BW was used.

Stillbirth definition

It is noted that to reach the level 1 intrapartum stillbirth case definition, GA level 1 is required, whereas the intrapartum stillbirth case definition additionally allows for GA level 2.

Following prior communications with the authors, in the present study a record of the absence of at least one specific sign of life was required.

Finally, the case definition would be easier to implement if the combination of criteria required (AND/OR) would be more clearly indicated.

Congenital microcephaly case definition (postnatally diagnosed) definition

The congenital microcephaly case definition recommends the use of the WHO growth reference charts if GA ≥37 weeks and the Intergrowth-21^st^ reference charts for GA 24-36 weeks. However, the WHO growth reference chart is based solely on weeks since birth and not on GA, and the Intergrowth-21^st^ reference charts cover all the GA. Thus, we used the Intergrowth-21st reference charts for all the GA.

The threshold to diagnose congenital microcephaly is defined as “head circumference 2 standard deviations below the mean or <3 percentile [..]”. The reference charts present both z-scores (standard deviation) and percentiles. The number of cases identified varied slightly between the two thresholds. In this study, the 3rd percentile was used as it is most common in clinical practice.

Site investigators have expressed concerns using a single chart globally, stressing that differences in underlying population characteristics including size of newborns are known to vary across the world, and that the case definition does not account for neonates with a small head circumference due to (symmetric) intra-uterine growth restriction and without abnormal head.
