# Supplementary material for: WHO global vaccine safety multi-country collaboration project on safety in pregnancy: Assessing the level of diagnostic certainty using standardized case definitions for perinatal and neonatal outcomes and maternal immunization
Source: Vaccine X. 2021 Nov 3;9:100123. doi: 10.1016/j.jvacx.2021.100123 (PMC8605263; doi:10.1016/j.jvacx.2021.100123)
Supplement: Supplementary data S4 — Site-Specific Results (tables and figures) [file mmc5.docx]

**S4 – Additional results**

*Table S4-1.The median and inter-quartile range across sites of the percentage of subjects meeting the case definition, and, among those, the percentage classified to level 1 (most specific), 2, and 3 (least specific)*

|  | % of recruited cases that meet the GAIA definition | | % of cases classified to each level, among cases meeting the GAIA definition | | | | | |  |
| --- | --- | --- | --- | --- | --- | --- | --- | --- | --- |
|  |  |  | **Level 1** | | **Level 2** | | **Level 3** | |  |
|  | **Median** | **IQR** | **Median** | **IQR** | **Median** | **IQR** | **Median** | **IQR** | |
| Low Birthweight | 100 | 100 - 100 | 0 | 0 - 100 | 19 | 0 – 100 | 0 | 0 – 100 | |
| Preterm birth | 100 | 100 - 100 | 26 | 12 - 38 | 24 | 12 - 38 | 42 | 30 - 74 | |
| Small for gestational age | 63 | 0 - 79 | 0 | 14 - 73 | 53 | 14 - 73 | 38 | 15 – 72 | |
| Antepartum stillbirth | 67 | 45 - 89 | 59 | 0 – 0 | 0 | 0 - 0 | 39 | 26 – 85 | |
| Intrapartum stillbirth | 37 | 25 - 58 | 0 | 0 - 23 | 4 | 0 – 23 | 92 | 58 - 100 | |
| Neonatal death | 100 | 100 - 100 | 100 | 0 - 0 | 0 | 0 – 0 | 0 | 0 – 0 | |
| Neonatal BSI | 93 | 57 - 98 | 5 | 63 – 87 | 77 | 63 - 87 | 2 | 0 – 16 | |
| Neonatal meningitis | 0 | 0 - 33 | 25 | 0 - 33 | 14 | 0 – 33 | 36 | 0 - 80 | |
| Neonatal respiratory infection | 100 | 83 - 100 | 0 | 0 – 40 | 4 | 0 - 40 | 60 | 50 – 100 | |
| Congenital microcephaly | 67 | 0 - 93 | 0 | 0 – 91 | 80 | 0 - 91 | 20 | 9 - 50 | |
| Maternal immunization | 100 | 99 - 100 | 0% | 13 - 68 | 35 | 13 - 68 | 8 | 2 - 48 | |

BSI: bloodstream infection; IQR: inter-quartile range

Table S4-2. Low Birth Weight: Cases that were recruited, classified according to the GAIA case definition at any level, and classified by levels.

| Country | Site |  | Any level | | Level 1 (most specific) | | | Level 2 | | | Level 3 (least specific) | | |
| --- | --- | --- | --- | --- | --- | --- | --- | --- | --- | --- | --- | --- | --- |
|  |  | N recruited cases | N classified cases | % classified cases (95% CI) | n | % among recruited (95%CI) | % among classified cases (95% CI) | n | % among recruited (95%CI) | % among classified cases (95% CI) | n | % among recruited (95%CI) | % among classified cases (95% CI) |
| AFRO |  |  |  |  |  |  |  |  |  |  |  |  |  |
| Ghana | St Joseph's H | 100 | 100 | 100 (96.4-100) | 0 | 0 (0-3.6) | 0 (0-3.6) | 0 | 0 (0-3.6) | 0 (0-3.6) | 100 | 100 (96.4-100) | 100 (96.4-100) |
| Ghana | Ejisu H | 59 | 59 | 100 (93.9-100) | 0 | 0 (0-6.1) | 0 (0-6.1) | 59 | 100 (93.9-100) | 100 (93.9-100) | 0 | 0 (0-6.1) | 0 (0-6.1) |
| Ghana | Tema GH | 101 | 101 | 100 (96.4-100) | 0 | 0 (0-3.6) | 0 (0-3.6) | 0 | 0 (0-3.6) | 0 (0-3.6) | 101 | 100 (96.4-100) | 100 (96.4-100) |
| Ghana | Eastern RH | 101 | 101 | 100 (96.4-100) | 0 | 0 (0-3.6) | 0 (0-3.6) | 101 | 100 (96.4-100) | 100 (96.4-100) | 0 | 0 (0-3.6) | 0 (0-3.6) |
| Tanzania | Mbeya ZRH | 109 | 109 | 100 (96.7-100) | 0 | 0 (0-3.3) | 0 (0-3.3) | 50 | 45.9 (36.3-55.7) | 45.9 (36.3-55.7) | 59 | 54.1 (44.3-63.7) | 54.1 (44.3-63.7) |
| Tanzania | St Francis RH | 100 | 100 | 100 (96.4-100) | 0 | 0 (0-3.6) | 0 (0-3.6) | 0 | 0 (0-3.6) | 0 (0-3.6) | 100 | 100 (96.4-100) | 100 (96.4-100) |
| Tanzania | Mbeya RRH | 100 | 100 | 100 (96.4-100) | 0 | 0 (0-3.6) | 0 (0-3.6) | 100 | 100 (96.4-100) | 100 (96.4-100) | 0 | 0 (0-3.6) | 0 (0-3.6) |
| Zimbabwe | Mbare PC | 106 | 106 | 100 (96.6-100) | 0 | 0 (0-3.4) | 0 (0-3.4) | 0 | 0 (0-3.4) | 0 (0-3.4) | 106 | 100 (96.6-100) | 100 (96.6-100) |
| Zimbabwe | Mutare PH | 101 | 100 | 99 (94.6-100) | 0 | 0 (0-3.6) | 0 (0-3.6) | 0 | 0 (0-3.6) | 0 (0-3.6) | 100 | 99 (94.6-100) | 100 (96.4-100) |
| EMRO |  |  |  |  |  |  |  |  |  |  |  |  |  |
| Iran | Mahdieh H | 100 | 100 | 100 (96.4-100) | 0 | 0 (0-3.6) | 0 (0-3.6) | 100 | 100 (96.4-100) | 100 (96.4-100) | 0 | 0 (0-3.6) | 0 (0-3.6) |
| Iran | Shohada TH | 73 | 72 | 98.6 (92.6-100) | 58 | 79.5 (68.4-88) | 80.6 (69.5-88.9) | 14 | 19.2 (10.9-30.1) | 19.4 (11.1-30.5) | 0 | 0 (0-4.9) | 0 (0-5) |
| EURO |  |  |  |  |  |  |  |  |  |  |  |  |  |
| Spain | Castellon GUH | 104 | 104 | 100 (96.5-100) | 0 | 0 (0-3.5) | 0 (0-3.5) | 0 | 0 (0-3.5) | 0 (0-3.5) | 104 | 100 (96.5-100) | 100 (96.5-100) |
| Spain | Dr Peset UH | 47 | 47 | 100 (92.5-100) | 0 | 0 (0-7.5) | 0 (0-7.5) | 0 | 0 (0-7.5) | 0 (0-7.5) | 47 | 100 (92.5-100) | 100 (92.5-100) |
| SEARO |  |  |  |  |  |  |  |  |  |  |  |  |  |
| India | JSS H | 100 | 100 | 100 (96.4-100) | 0 | 0 (0-3.6) | 0 (0-3.6) | 100 | 100 (96.4-100) | 100 (96.4-100) | 0 | 0 (0-3.6) | 0 (0-3.6) |
| India | Grant GMC | 98 | 98 | 100 (96.3-100) | 0 | 0 (0-3.7) | 0 (0-3.7) | 40 | 40.8 (31-51.2) | 40.8 (31-51.2) | 58 | 59.2 (48.8-69) | 59.2 (48.8-69) |
| India | IMS SUM H | 100 | 100 | 100 (96.4-100) | 0 | 0 (0-3.6) | 0 (0-3.6) | 100 | 100 (96.4-100) | 100 (96.4-100) | 0 | 0 (0-3.6) | 0 (0-3.6) |
| India | Kasturba MC | 101 | 101 | 100 (96.4-100) | 101 | 100 (96.4-100) | 100 (96.4-100) | 0 | 0 (0-3.6) | 0 (0-3.6) | 0 | 0 (0-3.6) | 0 (0-3.6) |
| India | MP Shah MC | 118 | 118 | 100 (96.9-100) | 0 | 0 (0-3.1) | 0 (0-3.1) | 118 | 100 (96.9-100) | 100 (96.9-100) | 0 | 0 (0-3.1) | 0 (0-3.1) |
| India | SKIMS | 100 | 100 | 100 (96.4-100) | 100 | 100 (96.4-100) | 100 (96.4-100) | 0 | 0 (0-3.6) | 0 (0-3.6) | 0 | 0 (0-3.6) | 0 (0-3.6) |
| Nepal | Patan H | 101 | 101 | 100 (96.4-100) | 0 | 0 (0-3.6) | 0 (0-3.6) | 0 | 0 (0-3.6) | 0 (0-3.6) | 101 | 100 (96.4-100) | 100 (96.4-100) |
| Nepal | BP Koirala | 131 | 131 | 100 (97.2-100) | 0 | 0 (0-2.8) | 0 (0-2.8) | 131 | 100 (97.2-100) | 100 (97.2-100) | 0 | 0 (0-2.8) | 0 (0-2.8) |

Table S4-3. Preterm birth (extended): Cases that were recruited, classified according to the GAIA case definition at any level, and classified by level and also sublevels

| **Country** | **Site** |  | **Any level** | | **Level 1 (most specific)** | | | **Level 2A** | | | **Level 2B** | | |
| --- | --- | --- | --- | --- | --- | --- | --- | --- | --- | --- | --- | --- | --- |
|  |  | **N recruited cases** | **N classified cases** | **% classified among recruited (95%CI)** | **n** | **% among recruited (95%CI)** | **% among classified (95%CI)** | **n** | **% among recruited (95%CI)** | **% among classified (95%CI)** | **n** | **% among recruited (95%CI)** | **% among classified (95%CI)** |
| **AFRO** |  |  |  |  |  |  |  |  |  |  |  |  |  |
| Ghana | St Joseph's H | 88 | 88 | 100 (95.9-100) | 30 | 34.1 (24.3-45) | 34.1 (24.3-45) | 8 | 9.1 (4-17.1) | 9.1 (4-17.1) | 34 | 38.6 (28.4-49.6) | 38.6 (28.4-49.6) |
| Ghana | Ejisu H | 27 | 27 | 100 (87.2-100) | 7 | 25.9 (11.1-46.3) | 25.9 (11.1-46.3) | 8 | 29.6 (13.8-50.2) | 29.6 (13.8-50.2) | 4 | 14.8 (4.2-33.7) | 14.8 (4.2-33.7) |
| Ghana | Tema GH | 100 | 100 | 100 (96.4-100) | 3 | 3 (0.6-8.5) | 3 (0.6-8.5) | 1 | 1 (0-5.4) | 1 (0-5.4) | 0 | 0 (0-3.6) | 0 (0-3.6) |
| Ghana | Eastern RH | 100 | 100 | 100 (96.4-100) | 41 | 41 (31.3-51.3) | 41 (31.3-51.3) | 6 | 6 (2.2-12.6) | 6 (2.2-12.6) | 40 | 40 (30.3-50.3) | 40 (30.3-50.3) |
| Tanzania | Mbeya ZRH | 95 | 95 | 100 (96.2-100) | 2 | 2.1 (0.3-7.4) | 2.1 (0.3-7.4) | 22 | 23.2 (15.1-32.9) | 23.2 (15.1-32.9) | 1 | 1.1 (0-5.7) | 1.1 (0-5.7) |
| Tanzania | St Francis RH | 98 | 98 | 100 (96.3-100) | 0 | 0 (0-3.7) | 0 (0-3.7) | 3 | 3.1 (0.6-8.7) | 3.1 (0.6-8.7) | 0 | 0 (0-3.7) | 0 (0-3.7) |
| Tanzania | Mbeya RRH | 97 | 97 | 100 (96.3-100) | 0 | 0 (0-3.7) | 0 (0-3.7) | 12 | 12.4 (6.6-20.6) | 12.4 (6.6-20.6) | 0 | 0 (0-3.7) | 0 (0-3.7) |
| Zimbabwe | Mbare PC | 76 | 76 | 100 (95.3-100) | 0 | 0 (0-4.7) | 0 (0-4.7) | 3 | 3.9 (0.8-11.1) | 3.9 (0.8-11.1) | 2 | 2.6 (0.3-9.2) | 2.6 (0.3-9.2) |
| Zimbabwe | Mutare PH | 99 | 99 | 100 (96.3-100) | 3 | 3 (0.6-8.6) | 3 (0.6-8.6) | 22 | 22.2 (14.5-31.7) | 22.2 (14.5-31.7) | 3 | 3 (0.6-8.6) | 3 (0.6-8.6) |
| **EMRO** |  |  |  |  |  |  |  |  |  |  |  |  |  |
| Iran | Mahdieh H | 100 | 100 | 100 (96.4-100) | 0 | 0 (0-3.6) | 0 (0-3.6) | 1 | 1 (0-5.4) | 1 (0-5.4) | 0 | 0 (0-3.6) | 0 (0-3.6) |
| Iran | Shohada TH | 92 | 92 | 100 (96.1-100) | 36 | 39.1 (29.1-49.9) | 39.1 (29.1-49.9) | 1 | 1.1 (0-5.9) | 1.1 (0-5.9) | 1 | 1.1 (0-5.9) | 1.1 (0-5.9) |
| **EURO** |  |  |  |  |  |  |  |  |  |  |  |  |  |
| Spain | Castellon GUH | 100 | 100 | 100 (96.4-100) | 42 | 42 (32.2-52.3) | 42 (32.2-52.3) | 12 | 12 (6.4-20) | 12 (6.4-20) | 0 | 0 (0-3.6) | 0 (0-3.6) |
| Spain | Dr Peset UH | 45 | 45 | 100 (92.1-100) | 24 | 53.3 (37.9-68.3) | 53.3 (37.9-68.3) | 8 | 17.8 (8-32.1) | 17.8 (8-32.1) | 0 | 0 (0-7.9) | 0 (0-7.9) |
| **SEARO** |  |  |  |  |  |  |  |  |  |  |  |  |  |
| India | JSS H | 99 | 99 | 100 (96.3-100) | 56 | 56.6 (46.2-66.5) | 56.6 (46.2-66.5) | 11 | 11.1 (5.7-19) | 11.1 (5.7-19) | 1 | 1 (0-5.5) | 1 (0-5.5) |
| India | Grant GMC | 96 | 96 | 100 (96.2-100) | 15 | 15.6 (9-24.5) | 15.6 (9-24.5) | 38 | 39.6 (29.7-50.1) | 39.6 (29.7-50.1) | 6 | 6.2 (2.3-13.1) | 6.2 (2.3-13.1) |
| India | IMS SUM H | 99 | 99 | 100 (96.3-100) | 28 | 28.3 (19.7-38.2) | 28.3 (19.7-38.2) | 29 | 29.3 (20.6-39.3) | 29.3 (20.6-39.3) | 0 | 0 (0-3.7) | 0 (0-3.7) |
| India | Kasturba MC | 100 | 100 | 100 (96.4-100) | 42 | 42 (32.2-52.3) | 42 (32.2-52.3) | 18 | 18 (11-26.9) | 18 (11-26.9) | 0 | 0 (0-3.6) | 0 (0-3.6) |
| India | MP Shah MC | 97 | 97 | 100 (96.3-100) | 5 | 5.2 (1.7-11.6) | 5.2 (1.7-11.6) | 33 | 34 (24.7-44.3) | 34 (24.7-44.3) | 0 | 0 (0-3.7) | 0 (0-3.7) |
| India | SKIMS | 101 | 101 | 100 (96.4-100) | 24 | 23.8 (15.9-33.3) | 23.8 (15.9-33.3) | 51 | 50.5 (40.4-60.6) | 50.5 (40.4-60.6) | 1 | 1 (0-5.4) | 1 (0-5.4) |
| Nepal | Patan H | 101 | 101 | 100 (96.4-100) | 64 | 63.4 (53.2-72.7) | 63.4 (53.2-72.7) | 26 | 25.7 (17.6-35.4) | 25.7 (17.6-35.4) | 2 | 2 (0.2-7) | 2 (0.2-7) |
| Nepal | BP Koirala | 104 | 104 | 100 (96.5-100) | 31 | 29.8 (21.2-39.6) | 29.8 (21.2-39.6) | 39 | 37.5 (28.2-47.5) | 37.5 (28.2-47.5) | 0 | 0 (0-3.5) | 0 (0-3.5) |

*Table S4-3. Pre-term birth, extended (contd) : Cases that were recruited, classified according to the GAIA case definition at any level, and classified by level and also sublevels*

| **Country** | **Site** |  | **Any level** | | **Level 3A** | | | **Level 3B** | | |
| --- | --- | --- | --- | --- | --- | --- | --- | --- | --- | --- |
|  |  | **N recruited cases** | **N classified cases** | **% classified among recruited (95%CI)** | **n** | **% among recruited (95%CI)** | **% among classified (95%CI)** | **n** | **% among recruited (95%CI)** | **% among classified (95%CI)** |
| **AFRO** |  |  |  |  |  |  |  |  |  |  |
| Ghana | St Joseph's H | 88 | 88 | 100 (95.9-100) | 0 | 0 (0-4.1) | 0 (0-4.1) | 16 | 18.2 (10.8-27.8) | 18.2 (10.8-27.8) |
| Ghana | Ejisu H | 27 | 27 | 100 (87.2-100) | 4 | 14.8 (4.2-33.7) | 14.8 (4.2-33.7) | 4 | 14.8 (4.2-33.7) | 14.8 (4.2-33.7) |
| Ghana | Tema GH | 100 | 100 | 100 (96.4-100) | 0 | 0 (0-3.6) | 0 (0-3.6) | 96 | 96 (90.1-98.9) | 96 (90.1-98.9) |
| Ghana | Eastern RH | 100 | 100 | 100 (96.4-100) | 3 | 3 (0.6-8.5) | 3 (0.6-8.5) | 10 | 10 (4.9-17.6) | 10 (4.9-17.6) |
| Tanzania | Mbeya ZRH | 95 | 95 | 100 (96.2-100) | 53 | 55.8 (45.2-66) | 55.8 (45.2-66) | 17 | 17.9 (10.8-27.1) | 17.9 (10.8-27.1) |
| Tanzania | St Francis RH | 98 | 98 | 100 (96.3-100) | 88 | 89.8 (82-95) | 89.8 (82-95) | 7 | 7.1 (2.9-14.2) | 7.1 (2.9-14.2) |
| Tanzania | Mbeya RRH | 97 | 97 | 100 (96.3-100) | 83 | 85.6 (77-91.9) | 85.6 (77-91.9) | 2 | 2.1 (0.3-7.3) | 2.1 (0.3-7.3) |
| Zimbabwe | Mbare PC | 76 | 76 | 100 (95.3-100) | 69 | 90.8 (81.9-96.2) | 90.8 (81.9-96.2) | 2 | 2.6 (0.3-9.2) | 2.6 (0.3-9.2) |
| Zimbabwe | Mutare PH | 99 | 99 | 100 (96.3-100) | 63 | 63.6 (53.4-73.1) | 63.6 (53.4-73.1) | 8 | 8.1 (3.6-15.3) | 8.1 (3.6-15.3) |
| **EMRO** |  |  |  |  |  |  |  |  |  |  |
| Iran | Mahdieh H | 100 | 100 | 100 (96.4-100) | 68 | 68 (57.9-77) | 68 (57.9-77) | 31 | 31 (22.1-41) | 31 (22.1-41) |
| Iran | Shohada TH | 92 | 92 | 100 (96.1-100) | 1 | 1.1 (0-5.9) | 1.1 (0-5.9) | 53 | 57.6 (46.9-67.9) | 57.6 (46.9-67.9) |
| **EURO** |  |  |  |  |  |  |  |  |  |  |
| Spain | Castellon GUH | 100 | 100 | 100 (96.4-100) | 46 | 46 (36-56.3) | 46 (36-56.3) | 0 | 0 (0-3.6) | 0 (0-3.6) |
| Spain | Dr Peset UH | 45 | 45 | 100 (92.1-100) | 10 | 22.2 (11.2-37.1) | 22.2 (11.2-37.1) | 3 | 6.7 (1.4-18.3) | 6.7 (1.4-18.3) |
| **SEARO** |  |  |  |  |  |  |  |  |  |  |
| India | JSS H | 99 | 99 | 100 (96.3-100) | 29 | 29.3 (20.6-39.3) | 29.3 (20.6-39.3) | 2 | 2 (0.2-7.1) | 2 (0.2-7.1) |
| India | Grant GMC | 96 | 96 | 100 (96.2-100) | 27 | 28.1 (19.4-38.2) | 28.1 (19.4-38.2) | 10 | 10.4 (5.1-18.3) | 10.4 (5.1-18.3) |
| India | IMS SUM H | 99 | 99 | 100 (96.3-100) | 42 | 42.4 (32.5-52.8) | 42.4 (32.5-52.8) | 0 | 0 (0-3.7) | 0 (0-3.7) |
| India | Kasturba MC | 100 | 100 | 100 (96.4-100) | 40 | 40 (30.3-50.3) | 40 (30.3-50.3) | 0 | 0 (0-3.6) | 0 (0-3.6) |
| India | MP Shah MC | 97 | 97 | 100 (96.3-100) | 42 | 43.3 (33.3-53.7) | 43.3 (33.3-53.7) | 17 | 17.5 (10.6-26.6) | 17.5 (10.6-26.6) |
| India | SKIMS | 101 | 101 | 100 (96.4-100) | 25 | 24.8 (16.7-34.3) | 24.8 (16.7-34.3) | 0 | 0 (0-3.6) | 0 (0-3.6) |
| Nepal | Patan H | 101 | 101 | 100 (96.4-100) | 9 | 8.9 (4.2-16.2) | 8.9 (4.2-16.2) | 0 | 0 (0-3.6) | 0 (0-3.6) |
| Nepal | BP Koirala | 104 | 104 | 100 (96.5-100) | 30 | 28.8 (20.4-38.6) | 28.8 (20.4-38.6) | 4 | 3.8 (1.1-9.6) | 3.8 (1.1-9.6) |

Table S4-4. Small for Gestational Age (extended): Cases that were recruited, classified according to the GAIA case definition at any level, and classified by level and sublevels

| **Country** | | **Site** |  | **Any level** | | **Level 1 (most specific)** | | | **Level 2A** | | | **Level 2B** | | |
| --- | --- | --- | --- | --- | --- | --- | --- | --- | --- | --- | --- | --- | --- | --- |
|  | |  | **N recruited cases** | **N classified cases** | **% classified among recruited (95%CI)** | **n** | **% among recruited (95%CI)** | **% among classified (95%CI)** | **n** | **% among recruited (95%CI)** | **% among classified (95%CI)** | **n** | **% among recruited (95%CI)** | **% among classified (95%CI)** |
| **AFRO** |  | |  |  |  |  |  |  |  |  |  |  |  |  |
| Ghana | St Joseph's H | | 82 | 0 | 0 (0-4.4) | 0 | 0 (0-4.4) |  | 0 | 0 (0-4.4) |  | 0 | 0 (0-4.4) |  |
| Ghana | Ejisu H | | 18 | 16 | 88.9 (65.3-98.6) | 0 | 0 (0-18.5) | 0 (0-20.6) | 16 | 88.9 (65.3-98.6) | 100 (79.4-100) | 0 | 0 (0-18.5) | 0 (0-20.6) |
| Ghana | Tema GH | | 99 | 0 | 0 (0-3.7) | 0 | 0 (0-3.7) |  | 0 | 0 (0-3.7) |  | 0 | 0 (0-3.7) |  |
| Ghana | Eastern RH | | 100 | 73 | 73 (63.2-81.4) | 0 | 0 (0-3.6) | 0 (0-4.9) | 33 | 33 (23.9-43.1) | 45.2 (33.5-57.3) | 39 | 39 (29.4-49.3) | 53.4 (41.4-65.2) |
| Tanzania | Mbeya ZRH | | 83 | 26 | 31.3 (21.6-42.4) | 0 | 0 (0-4.3) | 0 (0-13.2) | 2 | 2.4 (0.3-8.4) | 7.7 (0.9-25.1) | 1 | 1.2 (0-6.5) | 3.8 (0.1-19.6) |
| Tanzania | St Francis RH | |  |  |  |  |  |  |  |  |  |  |  |  |
| Tanzania | Mbeya RRH | | 99 | 75 | 75.8 (66.1-83.8) | 0 | 0 (0-3.7) | 0 (0-4.8) | 13 | 13.1 (7.2-21.4) | 17.3 (9.6-27.8) | 1 | 1 (0-5.5) | 1.3 (0-7.2) |
| Zimbabwe | Mbare PC | | 74 | 65 | 87.8 (78.2-94.3) | 0 | 0 (0-4.9) | 0 (0-5.5) | 5 | 6.8 (2.2-15.1) | 7.7 (2.5-17) | 0 | 0 (0-4.9) | 0 (0-5.5) |
| Zimbabwe | Mutare PH | | 86 | 0 | 0 (0-4.2) | 0 | 0 (0-4.2) |  | 0 | 0 (0-4.2) |  | 0 | 0 (0-4.2) |  |
| **EMRO** |  | |  |  |  |  |  |  |  |  |  |  |  |  |
| Iran | Mahdieh H | | 102 | 70 | 68.6 (58.7-77.5) | 0 | 0 (0-3.6) | 0 (0-5.1) | 3 | 2.9 (0.6-8.4) | 4.3 (0.9-12) | 0 | 0 (0-3.6) | 0 (0-5.1) |
| Iran | Shohada TH | | 16 | 6 | 37.5 (15.2-64.6) | 5 | 31.2 (11-58.7) | 83.3 (35.9-99.6) | 1 | 6.2 (0.2-30.2) | 16.7 (0.4-64.1) | 0 | 0 (0-20.6) | 0 (0-45.9) |
| **EURO** |  | |  |  |  |  |  |  |  |  |  |  |  |  |
| Spain | Castellon GUH | | 57 | 0 | 0 (0-6.3) | 0 | 0 (0-6.3) |  | 0 | 0 (0-6.3) |  | 0 | 0 (0-6.3) |  |
| Spain | Dr Peset UH | | 48 | 0 | 0 (0-7.4) | 0 | 0 (0-7.4) |  | 0 | 0 (0-7.4) |  | 0 | 0 (0-7.4) |  |
| **SEARO** |  | |  |  |  |  |  |  |  |  |  |  |  |  |
| India | JSS H | | 85 | 76 | 89.4 (80.8-95) | 0 | 0 (0-4.2) | 0 (0-4.7) | 58 | 68.2 (57.2-77.9) | 76.3 (65.2-85.3) | 0 | 0 (0-4.2) | 0 (0-4.7) |
| India | Grant GMC | | 24 | 14 | 58.3 (36.6-77.9) | 0 | 0 (0-14.2) | 0 (0-23.2) | 8 | 33.3 (15.6-55.3) | 57.1 (28.9-82.3) | 1 | 4.2 (0.1-21.1) | 7.1 (0.2-33.9) |
| India | IMS SUM H | | 98 | 74 | 75.5 (65.8-83.6) | 0 | 0 (0-3.7) | 0 (0-4.9) | 41 | 41.8 (31.9-52.2) | 55.4 (43.4-67) | 0 | 0 (0-3.7) | 0 (0-4.9) |
| India | Kasturba MC | | 100 | 99 | 99 (94.6-100) | 47 | 47 (36.9-57.2) | 47.5 (37.3-57.8) | 13 | 13 (7.1-21.2) | 13.1 (7.2-21.4) | 0 | 0 (0-3.6) | 0 (0-3.7) |
| India | MP Shah MC | | 91 | 44 | 48.4 (37.7-59.1) | 0 | 0 (0-4) | 0 (0-8) | 23 | 25.3 (16.7-35.5) | 52.3 (36.7-67.5) | 1 | 1.1 (0-6) | 2.3 (0.1-12) |
| India | SKIMS | | 34 | 29 | 85.3 (68.9-95) | 10 | 29.4 (15.1-47.5) | 34.5 (17.9-54.3) | 14 | 41.2 (24.6-59.3) | 48.3 (29.4-67.5) | 1 | 2.9 (0.1-15.3) | 3.4 (0.1-17.8) |
| Nepal | Patan H | | 66 | 0 | 0 (0-5.4) | 0 | 0 (0-5.4) |  | 0 | 0 (0-5.4) |  | 0 | 0 (0-5.4) |  |
| Nepal | BP Koirala | | 105 | 81 | 77.1 (67.9-84.8) | 0 | 0 (0-3.5) | 0 (0-4.5) | 65 | 61.9 (51.9-71.2) | 80.2 (69.9-88.3) | 0 | 0 (0-3.5) | 0 (0-4.5) |

*Table S4-4. Small for gestational age, extended (contd). Cases that were recruited, classified according to the GAIA case definition at any level, and classified by level and also sublevels*

| **Country** | **Site** |  | **Any level** | | **Level 3A** | | | **Level 3B** | | |
| --- | --- | --- | --- | --- | --- | --- | --- | --- | --- | --- |
|  |  | **N recruited cases** | **N classified cases** | **% classified among recruited (95%CI)** | **n** | **% among recruited (95%CI)** | **% among classified (95%CI)** | **n** | **% among recruited (95%CI)** | **% among classified (95%CI)** |
| AFRO |  |  |  |  |  |  |  |  |  |  |
| Ghana | St Joseph's H | 82 | 0 | 0 (0-4.4) | 0 | 0 (0-4.4) |  | 0 | 0 (0-4.4) |  |
| Ghana | Ejisu H | 18 | 16 | 88.9 (65.3-98.6) | 0 | 0 (0-18.5) | 0 (0-20.6) | 0 | 0 (0-18.5) | 0 (0-20.6) |
| Ghana | Tema GH | 99 | 0 | 0 (0-3.7) | 0 | 0 (0-3.7) |  | 0 | 0 (0-3.7) |  |
| Ghana | Eastern RH | 100 | 73 | 73 (63.2-81.4) | 1 | 1 (0-5.4) | 1.4 (0-7.4) | 0 | 0 (0-3.6) | 0 (0-4.9) |
| Tanzania | Mbeya ZRH | 83 | 26 | 31.3 (21.6-42.4) | 23 | 27.7 (18.4-38.6) | 88.5 (69.8-97.6) | 0 | 0 (0-4.3) | 0 (0-13.2) |
| Tanzania | St Francis RH |  |  |  |  |  |  |  |  |  |
| Tanzania | Mbeya RRH | 99 | 75 | 75.8 (66.1-83.8) | 61 | 61.6 (51.3-71.2) | 81.3 (70.7-89.4) | 0 | 0 (0-3.7) | 0 (0-4.8) |
| Zimbabwe | Mbare PC | 74 | 65 | 87.8 (78.2-94.3) | 60 | 81.1 (70.3-89.3) | 92.3 (83-97.5) | 0 | 0 (0-4.9) | 0 (0-5.5) |
| Zimbabwe | Mutare PH | 86 | 0 | 0 (0-4.2) | 0 | 0 (0-4.2) |  | 0 | 0 (0-4.2) |  |
| EMRO |  |  |  |  |  |  |  |  |  |  |
| Iran | Mahdieh H | 102 | 70 | 68.6 (58.7-77.5) | 67 | 65.7 (55.6-74.8) | 95.7 (88-99.1) | 0 | 0 (0-3.6) | 0 (0-5.1) |
| Iran | Shohada TH | 16 | 6 | 37.5 (15.2-64.6) | 0 | 0 (0-20.6) | 0 (0-45.9) | 0 | 0 (0-20.6) | 0 (0-45.9) |
| EURO |  |  |  |  |  |  |  |  |  |  |
| Spain | Castellon GUH | 57 | 0 | 0 (0-6.3) | 0 | 0 (0-6.3) |  | 0 | 0 (0-6.3) |  |
| Spain | Dr Peset UH | 48 | 0 | 0 (0-7.4) | 0 | 0 (0-7.4) |  | 0 | 0 (0-7.4) |  |
| SEARO |  |  |  |  |  |  |  |  |  |  |
| India | JSS H | 85 | 76 | 89.4 (80.8-95) | 18 | 21.2 (13.1-31.4) | 23.7 (14.7-34.8) | 0 | 0 (0-4.2) | 0 (0-4.7) |
| India | Grant GMC | 24 | 14 | 58.3 (36.6-77.9) | 5 | 20.8 (7.1-42.2) | 35.7 (12.8-64.9) | 0 | 0 (0-14.2) | 0 (0-23.2) |
| India | IMS SUM H | 98 | 74 | 75.5 (65.8-83.6) | 33 | 33.7 (24.4-43.9) | 44.6 (33-56.6) | 0 | 0 (0-3.7) | 0 (0-4.9) |
| India | Kasturba MC | 100 | 99 | 99 (94.6-100) | 39 | 39 (29.4-49.3) | 39.4 (29.7-49.7) | 0 | 0 (0-3.6) | 0 (0-3.7) |
| India | MP Shah MC | 91 | 44 | 48.4 (37.7-59.1) | 20 | 22 (14-31.9) | 45.5 (30.4-61.2) | 0 | 0 (0-4) | 0 (0-8) |
| India | SKIMS | 34 | 29 | 85.3 (68.9-95) | 4 | 11.8 (3.3-27.5) | 13.8 (3.9-31.7) | 0 | 0 (0-10.3) | 0 (0-11.9) |
| Nepal | Patan H | 66 | 0 | 0 (0-5.4) | 0 | 0 (0-5.4) |  | 0 | 0 (0-5.4) |  |
| Nepal | BP Koirala | 105 | 81 | 77.1 (67.9-84.8) | 16 | 15.2 (9-23.6) | 19.8 (11.7-30.1) | 0 | 0 (0-3.5) | 0 (0-4.5) |

Table S4-5: Antepartum stillbirth: Cases that were recruited, classified according to the GAIA case definition at any level, and classified by level

| Country | Site |  | Any level | | Level 1 (most specific) | | | Level 2 | | | Level 3 (least specific) | | |
| --- | --- | --- | --- | --- | --- | --- | --- | --- | --- | --- | --- | --- | --- |
|  |  | N recruited cases | N classified cases | % classified among recruited (95%CI) | n | % among recruited (95%CI) | % among classified (95%CI) | n | % among recruited (95%CI) | % among classified (95%CI) | n | % among recruited (95%CI) | % among classified (95%CI) |
| AFRO |  |  |  |  |  |  |  |  |  |  |  |  |  |
| Ghana | St Joseph's H | 16 | 12 | 75 (47.6-92.7) | 7 | 43.8 (19.8-70.1) | 58.3 (27.7-84.8) | 1 | 6.2 (0.2-30.2) | 8.3 (0.2-38.5) | 4 | 25 (7.3-52.4) | 33.3 (9.9-65.1) |
| Ghana | Ejisu H | 5 | 5 | 100 (47.8-100) | 3 | 60 (14.7-94.7) | 60 (14.7-94.7) | 0 | 0 (0-52.2) | 0 (0-52.2) | 2 | 40 (5.3-85.3) | 40 (5.3-85.3) |
| Ghana | Tema GH | 6 | 2 | 33.3 (4.3-77.7) | 1 | 16.7 (0.4-64.1) | 50 (1.3-98.7) | 0 | 0 (0-45.9) | 0 (0-84.2) | 1 | 16.7 (0.4-64.1) | 50 (1.3-98.7) |
| Ghana | Eastern RH | 93 | 89 | 95.7 (89.4-98.8) | 66 | 71 (60.6-79.9) | 74.2 (63.8-82.9) | 0 | 0 (0-3.9) | 0 (0-4.1) | 23 | 24.7 (16.4-34.8) | 25.8 (17.1-36.2) |
| Tanzania | Mbeya ZRH | 81 | 72 | 88.9 (80-94.8) | 9 | 11.1 (5.2-20) | 12.5 (5.9-22.4) | 0 | 0 (0-4.5) | 0 (0-5) | 63 | 77.8 (67.2-86.3) | 87.5 (77.6-94.1) |
| Tanzania | St Francis RH | 51 | 28 | 54.9 (40.3-68.9) | 0 | 0 (0-7) | 0 (0-12.3) | 0 | 0 (0-7) | 0 (0-12.3) | 28 | 54.9 (40.3-68.9) | 100 (87.7-100) |
| Tanzania | Mbeya RRH | 57 | 12 | 21.1 (11.4-33.9) | 1 | 1.8 (0-9.4) | 8.3 (0.2-38.5) | 0 | 0 (0-6.3) | 0 (0-26.5) | 11 | 19.3 (10-31.9) | 91.7 (61.5-99.8) |
| Zimbabwe | Mbare PC | 3 | 2 | 66.7 (9.4-99.2) | 0 | 0 (0-70.8) | 0 (0-84.2) | 0 | 0 (0-70.8) | 0 (0-84.2) | 2 | 66.7 (9.4-99.2) | 100 (15.8-100) |
| Zimbabwe | Mutare PH | 40 | 25 | 62.5 (45.8-77.3) | 6 | 15 (5.7-29.8) | 24 (9.4-45.1) | 0 | 0 (0-8.8) | 0 (0-13.7) | 19 | 47.5 (31.5-63.9) | 76 (54.9-90.6) |
| EMRO |  |  |  |  |  |  |  |  |  |  |  |  |  |
| Iran | Mahdieh H | 77 | 33 | 42.9 (31.6-54.6) | 1 | 1.3 (0-7) | 3 (0.1-15.8) | 0 | 0 (0-4.7) | 0 (0-10.6) | 32 | 41.6 (30.4-53.4) | 97 (84.2-99.9) |
| Iran | Shohada TH | 19 | 10 | 52.6 (28.9-75.6) | 6 | 31.6 (12.6-56.6) | 60 (26.2-87.8) | 0 | 0 (0-17.6) | 0 (0-30.8) | 4 | 21.1 (6.1-45.6) | 40 (12.2-73.8) |
| EURO |  |  |  |  |  |  |  |  |  |  |  |  |  |
| Spain | Castellon GUH | NA | NA | NA | NA | NA | NA | NA | NA | NA | NA | NA | NA |
| Spain | Dr Peset UH | NA | NA | NA | NA | NA | NA | NA | NA | NA | NA | NA | NA |
| SEARO |  |  |  |  |  |  |  |  |  |  |  |  |  |
| India | JSS H | 10 | 2 | 20 (2.5-55.6) | 2 | 20 (2.5-55.6) | 100 (15.8-100) | 0 | 0 (0-30.8) | 0 (0-84.2) | 0 | 0 (0-30.8) | 0 (0-84.2) |
| India | Grant GMC | 34 | 0 | 0 (0-10.3) | 0 | 0 (0-10.3) | NA | 0 | 0 (0-10.3) | NA | 0 | 0 (0-10.3) | NA |
| India | IMS SUM H | 58 | 56 | 96.6 (88.1-99.6) | 35 | 60.3 (46.6-73) | 62.5 (48.5-75.1) | 0 | 0 (0-6.2) | 0 (0-6.4) | 21 | 36.2 (24-49.9) | 37.5 (24.9-51.5) |
| India | Kasturba MC | 29 | 24 | 82.8 (64.2-94.2) | 12 | 41.4 (23.5-61.1) | 50 (29.1-70.9) | 3 | 10.3 (2.2-27.4) | 12.5 (2.7-32.4) | 9 | 31 (15.3-50.8) | 37.5 (18.8-59.4) |
| India | MP Shah MC | 70 | 33 | 47.1 (35.1-59.4) | 22 | 31.4 (20.9-43.6) | 66.7 (48.2-82) | 5 | 7.1 (2.4-15.9) | 15.2 (5.1-31.9) | 6 | 8.6 (3.2-17.7) | 18.2 (7-35.5) |
| India | SKIMS | 35 | 32 | 91.4 (76.9-98.2) | 25 | 71.4 (53.7-85.4) | 78.1 (60-90.7) | 0 | 0 (0-10) | 0 (0-10.9) | 7 | 20 (8.4-36.9) | 21.9 (9.3-40) |
| Nepal | Patan H | 35 | 31 | 88.6 (73.3-96.8) | 28 | 80 (63.1-91.6) | 90.3 (74.2-98) | 0 | 0 (0-10) | 0 (0-11.2) | 3 | 8.6 (1.8-23.1) | 9.7 (2-25.8) |
| Nepal | BP Koirala | 88 | 76 | 86.4 (77.4-92.8) | 55 | 62.5 (51.5-72.6) | 72.4 (60.9-82) | 0 | 0 (0-4.1) | 0 (0-4.7) | 21 | 23.9 (15.4-34.1) | 27.6 (18-39.1) |

*Table S4-6. Intrapartum stillbirth: Cases that were recruited, classified according to the GAIA case definition at any level, and classified by level*

| Country | | Site |  | Any level | | Level 1 (most specific) | | | Level 2 | | | Level 3 (least specific) | | |
| --- | --- | --- | --- | --- | --- | --- | --- | --- | --- | --- | --- | --- | --- | --- |
|  | |  | N recruited cases | N classified cases | % classified among recruited (95%CI) | n | % among recruited (95%CI) | % among classified (95%CI) | n | % among recruited (95%CI) | % among classified (95%CI) | n | % among recruited (95%CI) | % among classified (95%CI) |
| AFRO |  | |  |  |  |  |  |  |  |  |  |  |  |  |
| Ghana | St Joseph's H | | 13 | 12 | 92.3 (64-99.8) | 3 | 23.1 (5-53.8) | 25 (5.5-57.2) | 3 | 23.1 (5-53.8) | 25 (5.5-57.2) | 6 | 46.2 (19.2-74.9) | 50 (21.1-78.9) |
| Ghana | Ejisu H | | 7 | 5 | 71.4 (29-96.3) | 1 | 14.3 (0.4-57.9) | 20 (0.5-71.6) | 1 | 14.3 (0.4-57.9) | 20 (0.5-71.6) | 3 | 42.9 (9.9-81.6) | 60 (14.7-94.7) |
| Ghana | Tema GH | | 93 | 23 | 24.7 (16.4-34.8) | 0 | 0 (0-3.9) | 0 (0-14.8) | 2 | 2.2 (0.3-7.6) | 8.7 (1.1-28) | 21 | 22.6 (14.6-32.4) | 91.3 (72-98.9) |
| Ghana | Eastern RH | | 7 | 7 | 100 (59-100) | 2 | 28.6 (3.7-71) | 28.6 (3.7-71) | 2 | 28.6 (3.7-71) | 28.6 (3.7-71) | 3 | 42.9 (9.9-81.6) | 42.9 (9.9-81.6) |
| Tanzania | Mbeya ZRH | | 21 | 9 | 42.9 (21.8-66) | 0 | 0 (0-16.1) | 0 (0-33.6) | 2 | 9.5 (1.2-30.4) | 22.2 (2.8-60) | 7 | 33.3 (14.6-57) | 77.8 (40-97.2) |
| Tanzania | St Francis RH | | 46 | 15 | 32.6 (19.5-48) | 0 | 0 (0-7.7) | 0 (0-21.8) | 0 | 0 (0-7.7) | 0 (0-21.8) | 15 | 32.6 (19.5-48) | 100 (78.2-100) |
| Tanzania | Mbeya RRH | | 29 | 10 | 34.5 (17.9-54.3) | 0 | 0 (0-11.9) | 0 (0-30.8) | 1 | 3.4 (0.1-17.8) | 10 (0.3-44.5) | 9 | 31 (15.3-50.8) | 90 (55.5-99.7) |
| Zimbabwe | Mbare PC | | 8 | 2 | 25 (3.2-65.1) | 0 | 0 (0-36.9) | 0 (0-84.2) | 0 | 0 (0-36.9) | 0 (0-84.2) | 2 | 25 (3.2-65.1) | 100 (15.8-100) |
| Zimbabwe | Mutare PH | | 10 | 5 | 50 (18.7-81.3) | 0 | 0 (0-30.8) | 0 (0-52.2) | 0 | 0 (0-30.8) | 0 (0-52.2) | 5 | 50 (18.7-81.3) | 100 (47.8-100) |
| EMRO | |  |  |  |  |  |  |  |  |  |  |  |  |  |
| Iran | | Mahdieh H | 9 | 2 | 22.2 (2.8-60) | 0 | 0 (0-33.6) | 0 (0-84.2) | 0 | 0 (0-33.6) | 0 (0-84.2) | 2 | 22.2 (2.8-60) | 100 (15.8-100) |
| Iran | | Shohada TH | 4 | 1 | 25 (0.6-80.6) | 0 | 0 (0-60.2) | 0 (0-97.5) | 0 | 0 (0-60.2) | 0 (0-97.5) | 1 | 25 (0.6-80.6) | 100 (2.5-100) |
| EURO | |  |  |  |  |  |  |  |  |  |  |  |  |  |
| Spain | | Castellon GUH | NA | NA | NA | NA | NA | NA | NA | NA | NA | NA | NA | NA |
| Spain | | Dr Peset UH | NA | NA | NA | NA | NA | NA | NA | NA | NA | NA | NA | NA |
| SEARO | |  |  |  |  |  |  |  |  |  |  |  |  |  |
| India | | JSS H | 11 | 1 | 9.1 (0.2-41.3) | 0 | 0 (0-28.5) | 0 (0-97.5) | 0 | 0 (0-28.5) | 0 (0-97.5) | 1 | 9.1 (0.2-41.3) | 100 (2.5-100) |
| India | | Grant GMC | 16 | 0 | 0 (0-20.6) | 0 | 0 (0-20.6) | NA | 0 | 0 (0-20.6) | NA | 0 | 0 (0-20.6) | NA |
| India | | IMS SUM H | NA | NA | NA | NA | NA | NA | NA | NA | NA | NA | NA | NA |
| India | | Kasturba MC | 4 | 2 | 50 (6.8-93.2) | 0 | 0 (0-60.2) | 0 (0-84.2) | 0 | 0 (0-60.2) | 0 (0-84.2) | 2 | 50 (6.8-93.2) | 100 (15.8-100) |
| India | | MP Shah MC | 23 | 14 | 60.9 (38.5-80.3) | 1 | 4.3 (0.1-21.9) | 7.1 (0.2-33.9) | 0 | 0 (0-14.8) | 0 (0-23.2) | 13 | 56.5 (34.5-76.8) | 92.9 (66.1-99.8) |
| India | | SKIMS | 8 | 0 | 0 (0-36.9) | 0 | 0 (0-36.9) | NA | 0 | 0 (0-36.9) | NA | 0 | 0 (0-36.9) | NA |
| Nepal | | Patan H | 5 | 2 | 40 (5.3-85.3) | 1 | 20 (0.5-71.6) | 50 (1.3-98.7) | 1 | 20 (0.5-71.6) | 50 (1.3-98.7) | 0 | 0 (0-52.2) | 0 (0-84.2) |
| Nepal | | BP Koirala | 12 | 9 | 75 (42.8-94.5) | 2 | 16.7 (2.1-48.4) | 22.2 (2.8-60) | 5 | 41.7 (15.2-72.3) | 55.6 (21.2-86.3) | 2 | 16.7 (2.1-48.4) | 22.2 (2.8-60) |

Table S4-7. Neonatal Death: Case classification according to GAIA definition

| Country | Site |  | Any level | | Level 1 (most specific) | | | Level 2 | | | Level 3 (least specific) | | |
| --- | --- | --- | --- | --- | --- | --- | --- | --- | --- | --- | --- | --- | --- |
|  |  | N recruited cases | N classified cases | % classified among recruited (95%CI) | n | % among recruited (95%CI) | % among classified (95%CI) | n | % among recruited (95%CI) | % among classified (95%CI) | n | % among recruited (95%CI) | % among classified (95%CI) |
| AFRO |  |  |  |  |  |  |  |  |  |  |  |  |  |
| Ghana | St Joseph's H | 25 | 24 | 96 (79.6-99.9) | 24 | 96 (79.6-99.9) | 100 (85.8-100) | 0 | 0 (0-13.7) | 0 (0-14.2) | 0 | 0 (0-13.7) | 0 (0-14.2) |
| Ghana | Ejisu H | NA | NA | NA | NA | NA | NA | NA | NA | NA | NA | NA | NA |
| Ghana | Tema GH | 63 | 63 | 100 (94.3-100) | 63 | 100 (94.3-100) | 100 (94.3-100) | 0 | 0 (0-5.7) | 0 (0-5.7) | 0 | 0 (0-5.7) | 0 (0-5.7) |
| Ghana | Eastern RH | 95 | 95 | 100 (96.2-100) | 94 | 98.9 (94.3-100) | 98.9 (94.3-100) | 0 | 0 (0-3.8) | 0 (0-3.8) | 1 | 1.1 (0-5.7) | 1.1 (0-5.7) |
| Tanzania | Mbeya ZRH | 83 | 83 | 100 (95.7-100) | 83 | 100 (95.7-100) | 100 (95.7-100) | 0 | 0 (0-4.3) | 0 (0-4.3) | 0 | 0 (0-4.3) | 0 (0-4.3) |
| Tanzania | St Francis RH | 63 | 63 | 100 (94.3-100) | 63 | 100 (94.3-100) | 100 (94.3-100) | 0 | 0 (0-5.7) | 0 (0-5.7) | 0 | 0 (0-5.7) | 0 (0-5.7) |
| Tanzania | Mbeya RRH | 59 | 59 | 100 (93.9-100) | 59 | 100 (93.9-100) | 100 (93.9-100) | 0 | 0 (0-6.1) | 0 (0-6.1) | 0 | 0 (0-6.1) | 0 (0-6.1) |
| Zimbabwe | Mbare PC | 8 | 8 | 100 (63.1-100) | 8 | 100 (63.1-100) | 100 (63.1-100) | 0 | 0 (0-36.9) | 0 (0-36.9) | 0 | 0 (0-36.9) | 0 (0-36.9) |
| Zimbabwe | Mutare PH | 66 | 66 | 100 (94.6-100) | 66 | 100 (94.6-100) | 100 (94.6-100) | 0 | 0 (0-5.4) | 0 (0-5.4) | 0 | 0 (0-5.4) | 0 (0-5.4) |
| EMRO |  |  |  |  |  |  |  |  |  |  |  |  |  |
| Iran | Mahdieh H | 69 | 68 | 98.6 (92.2-100) | 68 | 98.6 (92.2-100) | 100 (94.7-100) | 0 | 0 (0-5.2) | 0 (0-5.3) | 0 | 0 (0-5.2) | 0 (0-5.3) |
| Iran | Shohada TH | 14 | 14 | 100 (76.8-100) | 14 | 100 (76.8-100) | 100 (76.8-100) | 0 | 0 (0-23.2) | 0 (0-23.2) | 0 | 0 (0-23.2) | 0 (0-23.2) |
| EURO |  |  |  |  |  |  |  |  |  |  |  |  |  |
| Spain | Castellon GUH | 3 | 3 | 100 (29.2-100) | 3 | 100 (29.2-100) | 100 (29.2-100) | 0 | 0 (0-70.8) | 0 (0-70.8) | 0 | 0 (0-70.8) | 0 (0-70.8) |
| Spain | Dr Peset UH | NA | NA | NA | NA | NA | NA | NA | NA | NA | NA | NA | NA |
| SEARO |  |  |  |  |  |  |  |  |  |  |  |  |  |
| India | JSS H | 14 | 14 | 100 (76.8-100) | 14 | 100 (76.8-100) | 100 (76.8-100) | 0 | 0 (0-23.2) | 0 (0-23.2) | 0 | 0 (0-23.2) | 0 (0-23.2) |
| India | Grant GMC | 28 | 28 | 100 (87.7-100) | 28 | 100 (87.7-100) | 100 (87.7-100) | 0 | 0 (0-12.3) | 0 (0-12.3) | 0 | 0 (0-12.3) | 0 (0-12.3) |
| India | IMS SUM H | 7 | 7 | 100 (59-100) | 7 | 100 (59-100) | 100 (59-100) | 0 | 0 (0-41) | 0 (0-41) | 0 | 0 (0-41) | 0 (0-41) |
| India | Kasturba MC | 28 | 28 | 100 (87.7-100) | 28 | 100 (87.7-100) | 100 (87.7-100) | 0 | 0 (0-12.3) | 0 (0-12.3) | 0 | 0 (0-12.3) | 0 (0-12.3) |
| India | MP Shah MC | 52 | 52 | 100 (93.2-100) | 52 | 100 (93.2-100) | 100 (93.2-100) | 0 | 0 (0-6.8) | 0 (0-6.8) | 0 | 0 (0-6.8) | 0 (0-6.8) |
| India | SKIMS | 32 | 32 | 100 (89.1-100) | 32 | 100 (89.1-100) | 100 (89.1-100) | 0 | 0 (0-10.9) | 0 (0-10.9) | 0 | 0 (0-10.9) | 0 (0-10.9) |
| Nepal | Patan H | 15 | 15 | 100 (78.2-100) | 15 | 100 (78.2-100) | 100 (78.2-100) | 0 | 0 (0-21.8) | 0 (0-21.8) | 0 | 0 (0-21.8) | 0 (0-21.8) |
| Nepal | BP Koirala | 27 | 27 | 100 (87.2-100) | 27 | 100 (87.2-100) | 100 (87.2-100) | 0 | 0 (0-12.8) | 0 (0-12.8) | 0 | 0 (0-12.8) | 0 (0-12.8) |

Table S4-8. Neonatal bloodstream infection: Cases that were recruited, classified according to the GAIA case definition at any level, and classified by level

| Country | Site |  | Any level | | Level 1 (most specific) | | | Level 2 | | | Level 3 (least specific) | | |
| --- | --- | --- | --- | --- | --- | --- | --- | --- | --- | --- | --- | --- | --- |
|  |  | N recruited cases | n classified cases | % Classified among recruited (95%CI) | n classified | % among recruited (95%CI) | % among classified (95%CI) | n classified | % among recruited (95%CI) | % among classified (95%CI) | n classified | % among recruited (95%CI) | % among classified (95%CI) |
| AFRO |  |  |  |  |  |  |  |  |  |  |  |  |  |
| Ghana | St Joseph's H | 63 | 25 | 39.7 (27.6-52.8) | 0 | 0 (0-5.7) | 0 (0-13.7) | 19 | 30.2 (19.2-43) | 76 (54.9-90.6) | 6 | 9.5 (3.6-19.6) | 24 (9.4-45.1) |
| Ghana | Ejisu H | 15 | 14 | 93.3 (68.1-99.8) | 0 | 0 (0-21.8) | 0 (0-23.2) | 13 | 86.7 (59.5-98.3) | 92.9 (66.1-99.8) | 1 | 6.7 (0.2-31.9) | 7.1 (0.2-33.9) |
| Ghana | Tema GH | 61 | 57 | 93.4 (84.1-98.2) | 2 | 3.3 (0.4-11.3) | 3.5 (0.4-12.1) | 47 | 77 (64.5-86.8) | 82.5 (70.1-91.3) | 8 | 13.1 (5.8-24.2) | 14 (6.3-25.8) |
| Ghana | Eastern RH | 49 | 28 | 57.1 (42.2-71.2) | 3 | 6.1 (1.3-16.9) | 10.7 (2.3-28.2) | 22 | 44.9 (30.7-59.8) | 78.6 (59-91.7) | 3 | 6.1 (1.3-16.9) | 10.7 (2.3-28.2) |
| Tanzania | Mbeya ZRH | 23 | 14 | 60.9 (38.5-80.3) | 0 | 0 (0-14.8) | 0 (0-23.2) | 9 | 39.1 (19.7-61.5) | 64.3 (35.1-87.2) | 5 | 21.7 (7.5-43.7) | 35.7 (12.8-64.9) |
| Tanzania | St Francis RH | 65 | 39 | 60 (47.1-72) | 0 | 0 (0-5.5) | 0 (0-9) | 29 | 44.6 (32.3-57.5) | 74.4 (57.9-87) | 10 | 15.4 (7.6-26.5) | 25.6 (13-42.1) |
| Tanzania | Mbeya RRH | 79 | 23 | 29.1 (19.4-40.4) | 0 | 0 (0-4.6) | 0 (0-14.8) | 8 | 10.1 (4.5-19) | 34.8 (16.4-57.3) | 15 | 19 (11-29.4) | 65.2 (42.7-83.6) |
| Zimbabwe | Mbare PC | 8 | 8 | 100 (63.1-100) | 0 | 0 (0-36.9) | 0 (0-36.9) | 8 | 100 (63.1-100) | 100 (63.1-100) | 0 | 0 (0-36.9) | 0 (0-36.9) |
| Zimbabwe | Mutare PH | 6 | 6 | 100 (54.1-100) | 2 | 33.3 (4.3-77.7) | 33.3 (4.3-77.7) | 4 | 66.7 (22.3-95.7) | 66.7 (22.3-95.7) | 0 | 0 (0-45.9) | 0 (0-45.9) |
| EMRO |  |  |  |  |  |  |  |  |  |  |  |  |  |
| Iran | Mahdieh H | 50 | 49 | 98 (89.4-99.9) | 25 | 50 (35.5-64.5) | 51 (36.3-65.6) | 24 | 48 (33.7-62.6) | 49 (34.4-63.7) | 0 | 0 (0-7.1) | 0 (0-7.3) |
| Iran | Shohada TH | 46 | 24 | 52.2 (36.9-67.1) | 0 | 0 (0-7.7) | 0 (0-14.2) | 19 | 41.3 (27-56.8) | 79.2 (57.8-92.9) | 5 | 10.9 (3.6-23.6) | 20.8 (7.1-42.2) |
| EURO |  |  |  |  |  |  |  |  |  |  |  |  |  |
| Spain | Castellon GUH | 7 | 7 | 100 (59-100) | 2 | 28.6 (3.7-71) | 28.6 (3.7-71) | 5 | 71.4 (29-96.3) | 71.4 (29-96.3) | 0 | 0 (0-41) | 0 (0-41) |
| Spain | Dr Peset UH | 7 | 3 | 42.9 (9.9-81.6) | 0 | 0 (0-41) | 0 (0-70.8) | 3 | 42.9 (9.9-81.6) | 100 (29.2-100) | 0 | 0 (0-41) | 0 (0-70.8) |
| SEARO |  |  |  |  |  |  |  |  |  |  |  |  |  |
| India | JSS H | 53 | 34 | 64.2 (49.8-76.9) | 5 | 9.4 (3.1-20.7) | 14.7 (5-31.1) | 29 | 54.7 (40.4-68.4) | 85.3 (68.9-95) | 0 | 0 (0-6.7) | 0 (0-10.3) |
| India | Grant GMC | 4 | 0 | 0 (0-60.2) | 0 | 0 (0-60.2) |  | 0 | 0 (0-60.2) |  | 0 | 0 (0-60.2) |  |
| India | IMS SUM H | 24 | 23 | 95.8 (78.9-99.9) | 9 | 37.5 (18.8-59.4) | 39.1 (19.7-61.5) | 14 | 58.3 (36.6-77.9) | 60.9 (38.5-80.3) | 0 | 0 (0-14.2) | 0 (0-14.8) |
| India | Kasturba MC | 59 | 58 | 98.3 (90.9-100) | 45 | 76.3 (63.4-86.4) | 77.6 (64.7-87.5) | 13 | 22 (12.3-34.7) | 22.4 (12.5-35.3) | 0 | 0 (0-6.1) | 0 (0-6.2) |
| India | MP Shah MC | 61 | 53 | 86.9 (75.8-94.2) | 0 | 0 (0-5.9) | 0 (0-6.7) | 51 | 83.6 (71.9-91.8) | 96.2 (87-99.5) | 2 | 3.3 (0.4-11.3) | 3.8 (0.5-13) |
| India | SKIMS | 34 | 34 | 100 (89.7-100) | 2 | 5.9 (0.7-19.7) | 5.9 (0.7-19.7) | 32 | 94.1 (80.3-99.3) | 94.1 (80.3-99.3) | 0 | 0 (0-10.3) | 0 (0-10.3) |
| Nepal | Patan H | 15 | 15 | 100 (78.2-100) | 6 | 40 (16.3-67.7) | 40 (16.3-67.7) | 8 | 53.3 (26.6-78.7) | 53.3 (26.6-78.7) | 1 | 6.7 (0.2-31.9) | 6.7 (0.2-31.9) |
| Nepal | BP Koirala | 54 | 53 | 98.1 (90.1-100) | 8 | 14.8 (6.6-27.1) | 15.1 (6.7-27.6) | 45 | 83.3 (70.7-92.1) | 84.9 (72.4-93.3) | 0 | 0 (0-6.6) | 0 (0-6.7) |

Table S4-9. Neonatal meningitis: Cases that were recruited, classified according to the GAIA case definition at any level, and classified by level

| Country | Site |  | Any level | | Level 1 (most specific) | | | Level 2 | | | Level 3 (least specific) | | |
| --- | --- | --- | --- | --- | --- | --- | --- | --- | --- | --- | --- | --- | --- |
|  |  | N recruited cases | n classified cases | % Classified among recruited (95%CI) | n classified | % among recruited (95%CI) | % among classified (95%CI) | n classified | % among recruited (95%CI) | % among classified (95%CI) | n classified | % among recruited (95%CI) | % among classified (95%CI) |
| AFRO |  |  |  |  |  |  |  |  |  |  |  |  |  |
| Ghana | Eastern RH | 3 | 1 | 33.3 (0.8-90.6) | 0 | 0 (0-70.8) | 0 (0-97.5) | 0 | 0 (0-70.8) | 0 (0-97.5) | 1 | 33.3 (0.8-90.6) | 100 (2.5-100) |
| Tanzania | Mbeya ZRH | 2 | 0 | 0 (0-84.2) | 0 | 0 (0-84.2) | - | 0 | 0 (0-84.2) | - | 0 | 0 (0-84.2) | - |
| Tanzania | St Francis RH | 4 | 0 | 0 (0-60.2) | 0 | 0 (0-60.2) | - | 0 | 0 (0-60.2) | - | 0 | 0 (0-60.2) | - |
| Tanzania | Mbeya RRH | 2 | 0 | 0 (0-84.2) | 0 | 0 (0-84.2) | - | 0 | 0 (0-84.2) | - | 0 | 0 (0-84.2) | - |
| EMRO |  |  |  |  |  |  |  |  |  |  |  |  |  |
| Iran | Shohada TH | 1 | 0 | 0 (0-97.5) | 0 | 0 (0-97.5) | - | 0 | 0 (0-97.5) | - | 0 | 0 (0-97.5) | - |
| EURO |  |  |  |  |  |  |  |  |  |  |  |  |  |
| Spain | Castellon GUH | 1 | 1 | 100 (2.5-100) | 1 | 100 (2.5-100) | 100 (2.5-100) | 0 | 0 (0-97.5) | 0 (0-97.5) | 0 | 0 (0-97.5) | 0 (0-97.5) |
| SEARO |  |  |  |  |  |  |  |  |  |  |  |  |  |
| India | JSS H | 2 | 0 | 0 (0-84.2) | 0 | 0 (0-84.2) | - | 0 | 0 (0-84.2) | - | 0 | 0 (0-84.2) |  |
| India | Grant GMC | 2 | 0 | 0 (0-84.2) | 0 | 0 (0-84.2) | - | 0 | 0 (0-84.2) | - | 0 | 0 (0-84.2) |  |
| India | IMS SUM H | 3 | 0 | 0 (0-70.8) | 0 | 0 (0-70.8) | - | 0 | 0 (0-70.8) | - | 0 | 0 (0-70.8) |  |
| India | Kasturba MC | 3 | 2 | 66.7 (9.4-99.2) | 1 | 33.3 (0.8-90.6) | 50 (1.3-98.7) | 1 | 33.3 (0.8-90.6) | 50 (1.3-98.7) | 0 | 0 (0-70.8) | 0 (0-84.2) |
| India | MP Shah MC | 1 | 0 | 0 (0-97.5) | 0 | 0 (0-97.5) | - | 0 | 0 (0-97.5) | - | 0 | 0 (0-97.5) |  |
| Nepal | Patan H | 22 | 0 | 0 (0-15.4) | 0 | 0 (0-15.4) | - | 0 | 0 (0-15.4) | - | 0 | 0 (0-15.4) | - |
| Nepal | BP Koirala | 18 | 11 | 61.1 (35.7-82.7) | 0 | 0 (0-18.5) | 0 (0-28.5) | 3 | 16.7 (3.6-41.4) | 27.3 (6-61) | 8 | 44.4 (21.5-69.2) | 72.7 (39-94) |

Table S4-10. Neonatal respiratory infection: Cases that were recruited, classified according to the GAIA case definition at any level, and classified by level

| Country | Site |  | Any level | | Level 1 (most specific) | | | Level 2 | | | Level 3 (least specific) | | |  |
| --- | --- | --- | --- | --- | --- | --- | --- | --- | --- | --- | --- | --- | --- | --- |
|  |  | N recruited cases | n classified cases | % Classified among recruited (95%CI) | n classified | % among recruited (95%CI) | % among classified (95%CI) | n classified | % among recruited (95%CI) | % among classified (95%CI) | n classified | % among recruited (95%CI) | % among classified (95%CI) | |
| AFRO |  |  |  |  |  |  |  |  |  |  |  |  |  | |
| Ghana | St Joseph's H | 12 | 8 | 66.7 (34.9-90.1) | 0 | 0 (0-26.5) | 0 (0-36.9) | 0 | 0 (0-26.5) | 0 (0-36.9) | 8 | 66.7 (34.9-90.1) | 100 (63.1-100) | |
| Ghana | Ejisu H | 0 | - | - | - | - | - | - | - | - | - | - | - | |
| Ghana | Tema GH | 6 | 5 | 83.3 (35.9-99.6) | 0 | 0 (0-45.9) | 0 (0-52.2) | 0 | 0 (0-45.9) | 0 (0-52.2) | 5 | 83.3 (35.9-99.6) | 100 (47.8-100) | |
| Ghana | Eastern RH | 0 | - | - | - | - | - | - | - | - | - | - | - | |
| Tanzania | Mbeya ZRH | 2 | 2 | 100 (15.8-100) | 0 | 0 (0-84.2) | 0 (0-84.2) | 1 | 50 (1.3-98.7) | 50 (1.3-98.7) | 1 | 50 (1.3-98.7) | 50 (1.3-98.7) | |
| Tanzania | St Francis RH | 4 | 3 | 75 (19.4-99.4) | 0 | 0 (0-60.2) | 0 (0-70.8) | 0 | 0 (0-60.2) | 0 (0-70.8) | 3 | 75 (19.4-99.4) | 100 (29.2-100) | |
| Tanzania | Mbeya RRH | 2 | 2 | 100 (15.8-100) | 0 | 0 (0-84.2) | 0 (0-84.2) | 0 | 0 (0-84.2) | 0 (0-84.2) | 2 | 100 (15.8-100) | 100 (15.8-100) | |
| Zimbabwe | Mbare PC | 0 | - | - | - | - | - | - | - | - | - | - | - | |
| Zimbabwe | Mutare PH | 1 | 1 | 100 (2.5-100) | 0 | 0 (0-97.5) | 0 (0-97.5) | 0 | 0 (0-97.5) | 0 (0-97.5) | 1 | 100 (2.5-100) | 100 (2.5-100) | |
| EMRO |  |  |  |  |  |  |  |  |  |  |  |  |  | |
| Iran | Mahdieh H | 11 | 11 | 100 (71.5-100) | 7 | 63.6 (30.8-89.1) | 63.6 (30.8-89.1) | 4 | 36.4 (10.9-69.2) | 36.4 (10.9-69.2) | 0 | 0 (0-28.5) | 0 (0-28.5) | |
| Iran | Shohada TH | 5 | 5 | 100 (47.8-100) | 0 | 0 (0-52.2) | 0 (0-52.2) | 2 | 40 (5.3-85.3) | 40 (5.3-85.3) | 3 | 60 (14.7-94.7) | 60 (14.7-94.7) | |
| EURO |  |  |  |  |  |  |  |  |  |  |  |  |  | |
| Spain | Castellon GUH | NA | NA | NA | NA | NA | NA | NA | NA | NA | NA | NA | NA | |
| Spain | Dr Peset UH | NA | NA | NA | NA | NA | NA | NA | NA | NA | NA | NA | NA | |
| SEARO |  |  |  |  |  |  |  |  |  |  |  |  |  | |
| India | JSS H | 5 | 5 | 100 (47.8-100) | 0 | 0 (0-52.2) | 0 (0-52.2) | 2 | 40 (5.3-85.3) | 40 (5.3-85.3) | 3 | 60 (14.7-94.7) | 60 (14.7-94.7) | |
| India | Grant GMC | 0 | - | - | - | - | - | - | - | - | - | - | - | |
| India | IMS SUM H | 0 | - | - | - | - | - | - | - | - | - | - | - | |
| India | Kasturba MC | 2 | 2 | 100 (15.8-100) | 1 | 50 (1.3-98.7) | 50 (1.3-98.7) | 0 | 0 (0-84.2) | 0 (0-84.2) | 1 | 50 (1.3-98.7) | 50 (1.3-98.7) | |
| India | MP Shah MC | 11 | 11 | 100 (71.5-100) | 0 | 0 (0-28.5) | 0 (0-28.5) | 10 | 90.9 (58.7-99.8) | 90.9 (58.7-99.8) | 1 | 9.1 (0.2-41.3) | 9.1 (0.2-41.3) | |
| India | SKIMS | NA | NA | NA | NA | NA | NA | NA | NA | NA | NA | NA | NA | |
| Nepal | Patan H | 65 | 53 | 81.5 (70-90.1) | 0 | 0 (0-5.5) | 0 (0-6.7) | 2 | 3.1 (0.4-10.7) | 3.8 (0.5-13) | 51 | 78.5 (66.5-87.7) | 96.2 (87-99.5) | |
| Nepal | BP Koirala | 29 | 29 | 100 (88.1-100) | 2 | 6.9 (0.8-22.8) | 6.9 (0.8-22.8) | 26 | 89.7 (72.6-97.8) | 89.7 (72.6-97.8) | 1 | 3.4 (0.1-17.8) | 3.4 (0.1-17.8) | |

Table S4-11. Congenital Microcephaly (extended): Cases that were recruited, classified according to the GAIA case definition at any level, and classified by level and also sublevels

| **Country** | **Site** |  | **Any level** | | **Level 1 (most specific)** | | | **Level 2A** | | | **Level 2B** | | | |
| --- | --- | --- | --- | --- | --- | --- | --- | --- | --- | --- | --- | --- | --- | --- |
|  |  | **N recruited cases** | **N classified cases** | **% classified among recruited (95%CI)** | **n** | **% among recruited (95%CI)** | **% among classified (95%CI)** | **n** | **% among recruited (95%CI)** | **% among classified (95%CI)** | | **n** | **% among recruited (95%CI)** | **% among classified (95%CI)** |
| **AFRO** |  |  |  |  |  |  |  |  |  |  |  | |  |  |
| Ghana | St Joseph's H | 2 | 2 | 100 (15.8-100) | 0 | 0 (0-84.2) | 0 (0-84.2) | 0 | 0 (0-84.2) | 0 (0-84.2) | 2 | | 100 (15.8-100) | 100 (15.8-100) |
| Ghana | Ejisu H | 1 | 1 | 100 (2.5-100) | 0 | 0 (0-97.5) | 0 (0-97.5) | 1 | 100 (2.5-100) | 100 (2.5-100) | 0 | | 0 (0-97.5) | 0 (0-97.5) |
| Ghana | Tema GH | 3 | 0 | 0 (0-70.8) | 0 | 0 (0-70.8) |  | 0 | 0 (0-70.8) |  | 0 | | 0 (0-70.8) |  |
| Ghana | Eastern RH | 2 | 0 | 0 (0-84.2) | 0 | 0 (0-84.2) |  | 0 | 0 (0-84.2) |  | 0 | | 0 (0-84.2) |  |
| Tanzania | Mbeya ZRH | 1 | 0 | 0 (0-97.5) | 0 | 0 (0-97.5) |  | 0 | 0 (0-97.5) |  | 0 | | 0 (0-97.5) |  |
| Tanzania | St Francis RH |  |  |  |  |  |  |  |  |  |  | |  |  |
| Tanzania | Mbeya RRH | 3 | 2 | 66.7 (9.4-99.2) | 1 | 33.3 (0.8-90.6) | 50 (1.3-98.7) | 0 | 0 (0-70.8) | 0 (0-84.2) | 0 | | 0 (0-70.8) | 0 (0-84.2) |
| Zimbabwe | Mbare PC | - | - | - | - | - | - | - | - | - | - | | - | - |
| Zimbabwe | Mutare PH | - | - | - | - | - | - | - | - | - | - | | - | - |
| **EMRO** |  |  |  |  |  |  |  |  |  |  |  | |  |  |
| Iran | Mahdieh H | 28 | 20 | 71.4 (51.3-86.8) | 0 | 0 (0-12.3) | 0 (0-16.8) | 17 | 60.7 (40.6-78.5) | 85 (62.1-96.8) | 0 | | 0 (0-12.3) | 0 (0-16.8) |
| Iran | Shohada TH | 7 | 0 | 0 (0-41) | 0 | 0 (0-41) |  | 0 | 0 (0-41) |  | 0 | | 0 (0-41) |  |
|  |  |  |  |  |  |  |  |  |  |  |  | |  |  |
| **EURO** |  |  |  |  |  |  |  |  |  |  |  | |  |  |
| Spain | Castellon GUH | 11 | 5 | 45.5 (16.7-76.6) | 0 | 0 (0-28.5) | 0 (0-52.2) | 4 | 36.4 (10.9-69.2) | 80 (28.4-99.5) | 0 | | 0 (0-28.5) | 0 (0-52.2) |
| Spain | Dr Peset UH | 14 | 3 | 21.4 (4.7-50.8) | 0 | 0 (0-23.2) | 0 (0-70.8) | 1 | 7.1 (0.2-33.9) | 33.3 (0.8-90.6) | 0 | | 0 (0-23.2) | 0 (0-70.8) |
| **SEARO** |  |  |  |  |  |  |  |  |  |  |  | |  |  |
| India | JSS H | 9 | 8 | 88.9 (51.8-99.7) | 0 | 0 (0-33.6) | 0 (0-36.9) | 7 | 77.8 (40-97.2) | 87.5 (47.3-99.7) | 0 | | 0 (0-33.6) | 0 (0-36.9) |
| India | Grant GMC |  |  |  |  |  |  |  |  |  |  | |  |  |
| India | IMS SUM H | 6 | 6 | 100 (54.1-100) | 4 | 66.7 (22.3-95.7) | 66.7 (22.3-95.7) | 0 | 0 (0-45.9) | 0 (0-45.9) | 0 | | 0 (0-45.9) | 0 (0-45.9) |
| India | Kasturba MC | 11 | 10 | 90.9 (58.7-99.8) | 0 | 0 (0-28.5) | 0 (0-30.8) | 8 | 72.7 (39-94) | 80 (44.4-97.5) | 0 | | 0 (0-28.5) | 0 (0-30.8) |
| India | MP Shah MC | 3 | 0 | 0 (0-70.8) | 0 | 0 (0-70.8) |  | 0 | 0 (0-70.8) |  | 0 | | 0 (0-70.8) |  |
| India | SKIMS | 1 | 1 | 100 (2.5-100) | 0 | 0 (0-97.5) | 0 (0-97.5) | 0 | 0 (0-97.5) | 0 (0-97.5) | 0 | | 0 (0-97.5) | 0 (0-97.5) |
| Nepal | Patan H | 3 | 1 | 33.3 (0.8-90.6) | 0 | 0 (0-70.8) | 0 (0-97.5) | 1 | 33.3 (0.8-90.6) | 100 (2.5-100) | 0 | | 0 (0-70.8) | 0 (0-97.5) |
| Nepal | BP Koirala | 15 | 14 | 93.3 (68.1-99.8) | 7 | 46.7 (21.3-73.4) | 50 (23-77) | 0 | 0 (0-21.8) | 0 (0-23.2) | 0 | | 0 (0-21.8) | 0 (0-23.2) |

*Table S4-11. Congenital Microcephaly, extended (contd) : Cases that were recruited, classified according to the GAIA case definition at any level, and classified by level and also sublevels*

| **Country** | **Site** |  | **Any level** | | **Level 3A** | | | **Level 3B** | | |
| --- | --- | --- | --- | --- | --- | --- | --- | --- | --- | --- |
|  |  | **N recruited cases** | **N classified cases** | **% classified among recruited (95%CI)** | **n** | **% among recruited (95%CI)** | **% among classified (95%CI)** | **n** | **% among recruited (95%CI)** | **% among classified (95%CI)** |
| **AFRO** |  |  |  |  |  |  |  |  |  |  |
| Ghana | St Joseph's H | 2 | 2 | 100 (15.8-100) | 0 | 0 (0-84.2) | 0 (0-84.2) | 0 | 0 (0-84.2) | 0 (0-84.2) |
| Ghana | Ejisu H | 1 | 1 | 100 (2.5-100) | 0 | 0 (0-97.5) | 0 (0-97.5) | 0 | 0 (0-97.5) | 0 (0-97.5) |
| Ghana | Tema GH | 3 | 0 | 0 (0-70.8) | 0 | 0 (0-70.8) |  | 0 | 0 (0-70.8) |  |
| Ghana | Eastern RH | 2 | 0 | 0 (0-84.2) | 0 | 0 (0-84.2) |  | 0 | 0 (0-84.2) |  |
| Tanzania | Mbeya ZRH | 1 | 0 | 0 (0-97.5) | 0 | 0 (0-97.5) |  | 0 | 0 (0-97.5) |  |
| Tanzania | St Francis RH | - | - | - | - | - | - | - | - |  |
| Tanzania | Mbeya RRH | 3 | 2 | 66.7 (9.4-99.2) | 1 | 33.3 (0.8-90.6) | 50 (1.3-98.7) | 0 | 0 (0-70.8) | 0 (0-84.2) |
| Zimbabwe | Mbare PC | - | - | - | - | - | - | - | - | - |
| Zimbabwe | Mutare PH | - | - | - | - | - | - | - | - | - |
| **EMRO** |  |  |  |  |  |  |  |  |  |  |
| Iran | Mahdieh H | 28 | 20 | 71.4 (51.3-86.8) | 3 | 10.7 (2.3-28.2) | 15 (3.2-37.9) | 0 | 0 (0-12.3) | 0 (0-16.8) |
| Iran | Shohada TH | 7 | 0 | 0 (0-41) | 0 | 0 (0-41) |  | 0 | 0 (0-41) |  |
| **EURO** |  |  |  |  |  |  |  |  |  |  |
| Spain | Castellon GUH | 11 | 5 | 45.5 (16.7-76.6) | 1 | 9.1 (0.2-41.3) | 20 (0.5-71.6) | 0 | 0 (0-28.5) | 0 (0-52.2) |
| Spain | Dr Peset UH | 14 | 3 | 21.4 (4.7-50.8) | 2 | 14.3 (1.8-42.8) | 66.7 (9.4-99.2) | 0 | 0 (0-23.2) | 0 (0-70.8) |
| **SEARO** |  |  |  |  |  |  |  |  |  |  |
| India | JSS H | 9 | 8 | 88.9 (51.8-99.7) | 1 | 11.1 (0.3-48.2) | 12.5 (0.3-52.7) | 0 | 0 (0-33.6) | 0 (0-36.9) |
| India | Grant GMC |  |  |  |  |  |  |  |  |  |
| India | IMS SUM H | 6 | 6 | 100 (54.1-100) | 2 | 33.3 (4.3-77.7) | 33.3 (4.3-77.7) | 0 | 0 (0-45.9) | 0 (0-45.9) |
| India | Kasturba MC | 11 | 10 | 90.9 (58.7-99.8) | 2 | 18.2 (2.3-51.8) | 20 (2.5-55.6) | 0 | 0 (0-28.5) | 0 (0-30.8) |
| India | MP Shah MC | 3 | 0 | 0 (0-70.8) | 0 | 0 (0-70.8) |  | 0 | 0 (0-70.8) |  |
| India | SKIMS | 1 | 1 | 100 (2.5-100) | 1 | 100 (2.5-100) | 100 (2.5-100) | 0 | 0 (0-97.5) | 0 (0-97.5) |
| Nepal | Patan H | 3 | 1 | 33.3 (0.8-90.6) | 0 | 0 (0-70.8) | 0 (0-97.5) | 0 | 0 (0-70.8) | 0 (0-97.5) |
| Nepal | BP Koirala | 15 | 14 | 93.3 (68.1-99.8) | 7 | 46.7 (21.3-73.4) | 50 (23-77) | 0 | 0 (0-21.8) | 0 (0-23.2) |

*Table S4-12. Vaccination status among mothers of recruited cases during pregnancy, and classification according to GAIA definition*

| Country | Site | N mothers assessed | Unknown vaccination status | Unvaccinated | Vaccinated | | |
| --- | --- | --- | --- | --- | --- | --- | --- |
|  |  |  | **n (% among assessed mothers)** | **n (% among assessed mothers)** | **N (% among assessed mothers)** | **Classified,**  **n** | **% Classified among vaccinated cases (95%CI)** |
| AFRO |  |  |  |  |  |  |  |
| Ghana | St Joseph's H | 284 | 7 (2.5) | 27 (9.5) | 250 (88) | 249 | 99.6 (97.8-100) |
| Ghana | Ejisu H | 94 | 21 (22.3) | 13 (13.8) | 60 (63.8) | 60 | 100 (94-100) |
| Ghana | Tema GH | 425 | 335 (78.8) | 12 (2.8) | 78 (18.4) | 71 | 91 (82.4-96.3) |
| Ghana | Eastern RH | 428 | 57 (13.3) | 65 (15.2) | 306 (71.5) | 306 | 100 (98.8-100) |
| Tanzania | Mbeya ZRH | 375 | 302 (80.5) | 6 (1.6) | 67 (17.9) | 64 | 95.5 (87.5-99.1) |
| Tanzania | St Francis RH | 343 | 77 (22.4) | 56 (16.3) | 210 (61.2) | 210 | 100 (98.3-100) |
| Tanzania | Mbeya RRH | 386 | 27 (7) | 30 (7.8) | 329 (85.2) | 329 | 100 (98.9-100) |
| Zimbabwe | Mbare PC | 183 | 58 (31.7) | 19 (10.4) | 106 (57.9) | 106 | 100 (96.6-100) |
| Zimbabwe | Mutare PH | 253 | 16 (6.3) | 26 (10.3) | 211 (83.4) | 211 | 100 (98.3-100) |
| EMRO |  |  |  |  |  |  |  |
| Iran | Mahdieh H | 402 | 340 (84.6) | 45 (11.2) | 17 (4.2) | 17 | 100 (80.5-100) |
| Iran | Shohada TH | 188 | 60 (31.9) | 128 (68.1) | 0 (0) | 0 |  |
| EURO |  |  |  |  |  |  |  |
| Spain | Castellon GUH | 161 | 9 (5.6) | 14 (8.7) | 138 (85.7) | 137 | 99.3 (96-100) |
| Spain | Dr Peset UH | 86 | 6 (7) | 4 (4.7) | 76 (88.4) | 75 | 98.7 (92.9-100) |
| SEARO |  |  |  |  |  |  |  |
| India | JSS H | 255 | 55 (21.6) | 4 (1.6) | 196 (76.9) | 193 | 98.5 (95.6-99.7) |
| India | Grant GMC | 204 | 33 (16.2) | 3 (1.5) | 168 (82.4) | 161 | 95.8 (91.6-98.3) |
| India | IMS SUM H | 270 | 0 (0) | 2 (0.7) | 268 (99.3) | 268 | 100 (98.6-100) |
| India | Kasturba MC | 285 | 50 (17.5) | 0 (0) | 235 (82.5) | 235 | 100 (98.4-100) |
| India | MP Shah MC | 436 | 14 (3.2) | 10 (2.3) | 412 (94.5) | 411 | 99.8 (98.7-100) |
| India | SKIMS | 271 | 1 (0.4) | 7 (2.6) | 263 (97) | 263 | 100 (98.6-100) |
| Nepal | Patan H | 298 | 105 (35.2) | 0 (0) | 193 (64.8) | 193 | 100 (98.1-100) |
| Nepal | BP Koirala | 373 | 1 (0.3) | 2 (0.5) | 370 (99.2) | 370 | 100 (99-100) |

*Table S4-13. Maternal immunization: Exposure classification according to GAIA definition*

| Country | Site | N Recruited cases* | Any level | Level 1 (most specific) | | | Level 2 | | | Level 3 (least specific) | | |
| --- | --- | --- | --- | --- | --- | --- | --- | --- | --- | --- | --- | --- |
|  |  |  | N Classified cases | n classified | % among mothers (95%CI) | % among classified (95%CI) | n classified | % among mothers (95%CI) | % among classified (95%CI) | n classified | % among mothers (95%CI) | % among classified (95%CI) |
| AFRO |  |  |  |  |  |  |  |  |  |  |  |  |
| Ghana | St Joseph's H | 284 | 249 | 0 | 0 (0-1.3) | 0 (0-1.5) | 247 | 87 (82.5-90.7) | 99.2 (97.1-99.9) | 2 | 0.7 (0.1-2.5) | 0.8 (0.1-2.9) |
| Ghana | Ejisu H | 94 | 60 | 54 | 57.4 (46.8-67.6) | 90 (79.5-96.2) | 5 | 5.3 (1.7-12) | 8.3 (2.8-18.4) | 1 | 1.1 (0-5.8) | 1.7 (0-8.9) |
| Ghana | Tema GH | 425 | 71 | 0 | 0 (0-0.9) | 0 (0-5.1) | 64 | 15.1 (11.8-18.8) | 90.1 (80.7-95.9) | 7 | 1.6 (0.7-3.4) | 9.9 (4.1-19.3) |
| Ghana | Eastern RH | 428 | 306 | 0 | 0 (0-0.9) | 0 (0-1.2) | 298 | 69.6 (65-74) | 97.4 (94.9-98.9) | 8 | 1.9 (0.8-3.6) | 2.6 (1.1-5.1) |
| Tanzania | Mbeya ZRH | 375 | 64 | 0 | 0 (0-1) | 0 (0-5.6) | 50 | 13.3 (10.1-17.2) | 78.1 (66-87.5) | 14 | 3.7 (2.1-6.2) | 21.9 (12.5-34) |
| Tanzania | St Francis RH | 343 | 210 | 0 | 0 (0-1.1) | 0 (0-1.7) | 181 | 52.8 (47.3-58.2) | 86.2 (80.8-90.6) | 29 | 8.5 (5.7-11.9) | 13.8 (9.4-19.2) |
| Tanzania | Mbeya RRH | 386 | 329 | 0 | 0 (0-1) | 0 (0-1.1) | 302 | 78.2 (73.8-82.3) | 91.8 (88.3-94.5) | 27 | 7 (4.7-10) | 8.2 (5.5-11.7) |
| Zimbabwe | Mbare PC | 183 | 106 | 55 | 30.1 (23.5-37.3) | 51.9 (42-61.7) | 37 | 20.2 (14.7-26.8) | 34.9 (25.9-44.8) | 14 | 7.7 (4.2-12.5) | 13.2 (7.4-21.2) |
| Zimbabwe | Mutare PH | 253 | 211 | 0 | 0 (0-1.4) | 0 (0-1.7) | 184 | 72.7 (66.8-78.1) | 87.2 (81.9-91.4) | 27 | 10.7 (7.2-15.1) | 12.8 (8.6-18.1) |
| EMRO |  |  |  |  |  |  |  |  |  |  |  |  |
| Iran | Mahdieh H | 402 | 17 | 0 | 0 (0-0.9) | 0 (0-19.5) | 0 | 0 (0-0.9) | 0 (0-19.5) | 17 | 4.2 (2.5-6.7) | 100 (80.5-100) |
| Iran | Shohada TH | 188 | 0 | 0 | 0 (0-1.9) |  | 0 | 0 (0-1.9) |  | 0 | 0 (0-1.9) |  |
| EURO |  |  |  |  |  |  |  |  |  |  |  |  |
| Spain | Castellon GUH | 161 | 137 | 0 | 0 (0-2.3) | 0 (0-2.7) | 109 | 67.7 (59.9-74.8) | 79.6 (71.8-86) | 28 | 17.4 (11.9-24.1) | 20.4 (14-28.2) |
| Spain | Dr Peset UH | 86 | 75 | 0 | 0 (0-4.2) | 0 (0-4.8) | 16 | 18.6 (11-28.4) | 21.3 (12.7-32.3) | 59 | 68.6 (57.7-78.2) | 78.7 (67.7-87.3) |
| SEARO |  |  |  |  |  |  |  |  |  |  |  |  |
| India | JSS H | 255 | 193 | 0 | 0 (0-1.4) | 0 (0-1.9) | 17 | 6.7 (3.9-10.5) | 8.8 (5.2-13.7) | 176 | 69 (63-74.6) | 91.2 (86.3-94.8) |
| India | Grant GMC | 204 | 161 | 0 | 0 (0-1.8) | 0 (0-2.3) | 99 | 48.5 (41.5-55.6) | 61.5 (53.5-69) | 62 | 30.4 (24.2-37.2) | 38.5 (31-46.5) |
| India | IMS SUM H | 270 | 268 | 0 | 0 (0-1.4) | 0 (0-1.4) | 119 | 44.1 (38.1-50.2) | 44.4 (38.4-50.6) | 149 | 55.2 (49-61.2) | 55.6 (49.4-61.6) |
| India | Kasturba MC | 285 | 235 | 0 | 0 (0-1.3) | 0 (0-1.6) | 48 | 16.8 (12.7-21.7) | 20.4 (15.5-26.2) | 187 | 65.6 (59.8-71.1) | 79.6 (73.8-84.5) |
| India | MP Shah MC | 436 | 411 | 0 | 0 (0-0.8) | 0 (0-0.9) | 201 | 46.1 (41.3-50.9) | 48.9 (44-53.9) | 210 | 48.2 (43.4-53) | 51.1 (46.1-56) |
| India | SKIMS | 271 | 263 | 0 | 0 (0-1.4) | 0 (0-1.4) | 261 | 96.3 (93.3-98.2) | 99.2 (97.3-99.9) | 2 | 0.7 (0.1-2.6) | 0.8 (0.1-2.7) |
| Nepal | Patan H | 298 | 193 | 0 | 0 (0-1.2) | 0 (0-1.9) | 104 | 34.9 (29.5-40.6) | 53.9 (46.6-61.1) | 89 | 29.9 (24.7-35.4) | 46.1 (38.9-53.4) |
| Nepal | BP Koirala | 373 | 370 | 0 | 0 (0-1) | 0 (0-1) | 4 | 1.1 (0.3-2.7) | 1.1 (0.3-2.7) | 366 | 98.1 (96.2-99.2) | 98.9 (97.3-99.7) |

*Table S4-14: Description of the elements which prevent the GAIA level 3 low birth weight cases to be classified as level 1 or 2**

| Country | Site | N classified at Level 3 | Wrong type of scale, n (%*) | Taring unknown or inappropriate, n(%*) | Graduation unknown or inappropriate, n(%*) | Calibration unknown or inappropriate, n(%*) | Baby weighed >24 hours after birth, n(%*) |
| --- | --- | --- | --- | --- | --- | --- | --- |
| AFRO |  |  |  |  |  |  |  |
| Ghana | St Joseph's H | 100 | 0 (0) | 0 (0) | 100 (100) | 100 (100) | 0 (0) |
| Ghana | Tema GH | 101 | 0 (0) | 0 (0) | 101 (100) | 0 (0) | 0 (0) |
| Tanzania | Mbeya ZRH | 59 | 0 (0) | 0 (0) | 59 (100) | 0 (0) | 0 (0) |
| Tanzania | St Francis RH | 100 | 100 (100) | 100 (100) | 100 (100) | 100 (100) | 0 (0) |
| Zimbabwe | Mbare PC | 106 | 0 (0) | 0 (0) | 0 (0) | 106 (100) | 0 (0) |
| Zimbabwe | Mutare PH | 100 | 0 (0) | 0 (0) | 100 (100) | 0 (0) | 0 (0) |
| EURO |  |  |  |  |  |  |  |
| Spain | Castellon GUH | 104 | 0 (0) | 0 (0) | 104 (100) | 104 (100) | 0 (0) |
| Spain | Dr Peset UH | 47 | 0 (0) | 0 (0) | 0 (0) | 47 (100) | 0 (0) |
| SEARO |  |  |  |  |  |  |  |
| India | Grant GMC | 58 | 58 (100) | 0 (0) | 0 (0) | 0 (0) | 0 (0) |
| Nepal | Patan H | 101 | 0 (0) | 0 (0) | 101 (100) | 101 (100) | 0 (0) |

*percentage among all level 3 cases for this outcome

*Table S4-15. Description of the elements which prevent the GAIA level 3 preterm birth cases to be classified as level 1 or 2*

| Country | Site | Level 3, N | No 1^st^ trimester US reported, n (%*) | 1^st^ trimester US reported, but information from US is insufficient**, n (%) | LMP available, but 2^nd^ trimester US is not reported or 2^nd^ trimester US information is insufficient**, and no 1^st^ trimester physical examination is available, n (%) | 2^nd^ trimester US is not reported but information is insufficient**, n (%) |
| --- | --- | --- | --- | --- | --- | --- |
| AFRO |  |  |  |  |  |  |
| Ghana | St Joseph's H | 16 | 16 (100) | 0 (0) | 0 (0) | 16 (100) |
| Ghana | Ejisu H | 8 | 6 (75) | 2 (25) | 4 (50) | 8 (100) |
| Ghana | Tema GH | 96 | 96 (100) | 0 (0) | 0 (0) | 96 (100) |
| Ghana | Eastern RH | 13 | 13 (100) | 0 (0) | 3 (23.1) | 13 (100) |
| Tanzania | Mbeya ZRH | 70 | 70 (100) | 0 (0) | 53 (75.7) | 70 (100) |
| Tanzania | St Francis RH | 95 | 95 (100) | 0 (0) | 88 (92.6) | 95 (100) |
| Tanzania | Mbeya RRH | 85 | 85 (100) | 0 (0) | 83 (97.6) | 85 (100) |
| Zimbabwe | Mbare PC | 71 | 71 (100) | 0 (0) | 69 (97.2) | 71 (100) |
| Zimbabwe | Mutare PH | 71 | 70 (98.6) | 1 (1.4) | 63 (88.7) | 71 (100) |
| EMRO |  |  |  |  |  |  |
| Iran | Mahdieh H | 99 | 23 (23.2) | 76 (76.8) | 68 (68.7) | 99 (100) |
| Iran | Shohada TH | 54 | 27 (50) | 27 (50) | 1 (1.9) | 54 (100) |
| EURO |  |  |  |  |  |  |
| Spain | Castellon GUH | 46 | 26 (56.5) | 20 (43.5) | 46 (100) | 46 (100) |
| Spain | Dr Peset UH | 13 | 10 (76.9) | 3 (23.1) | 10 (76.9) | 13 (100) |
| SEARO |  |  |  |  |  |  |
| India | JSS H | 31 | 31 (100) | 0 (0) | 29 (93.5) | 31 (100) |
| India | Grant GMC | 37 | 37 (100) | 0 (0) | 27 (73) | 37 (100) |
| India | IMS SUM H | 42 | 42 (100) | 0 (0) | 42 (100) | 42 (100) |
| India | Kasturba MC | 40 | 23 (57.5) | 17 (42.5) | 40 (100) | 40 (100) |
| India | MP Shah MC | 59 | 58 (98.3) | 1 (1.7) | 42 (71.2) | 59 (100) |
| India | SKIMS | 25 | 21 (84) | 4 (16) | 25 (100) | 25 (100) |
| Nepal | Patan H | 9 | 8 (88.9) | 1 (11.1) | 9 (100) | 9 (100) |
| Nepal | BP Koirala | 34 | 34 (100) | 0 (0) | 30 (88.2) | 34 (100) |

US: Ultrasound

*Table 4-16. Description of the reasons for recruited small for gestational age cases not being classifiable to level 3 using the GAIA definition. For each category, the percentage describes the proportion of unclassified recruited cases for which that was the reason why it could not be classified*

| Country | Site | N unclassified | Weight not <10^th^ percentile for GA on Intergrowth-21 chart, n (% of unclassified) | Information on GA insufficient, n (% of unclassified) | Baby not weighed within 48h of birth, n (% of unclassified) | Weight not obtained using appropriate method, n (% of unclassified) |
| --- | --- | --- | --- | --- | --- | --- |
| AFRO |  |  |  |  |  |  |
| Ghana | St Joseph's H | 82 | 10 (12.2) | 0 (0) | 0 (0) | 82 (100) |
| Ghana | Ejisu H | 2 | 2 (100) | 0 (0) | 0 (0) | 0 (0) |
| Ghana | Tema GH | 99 | 90 (90.9) | 0 (0) | 1 (1) | 99 (100) |
| Ghana | Eastern RH | 27 | 27 (100) | 0 (0) | 0 (0) | 0 (0) |
| Tanzania | Mbeya ZRH | 57 | 12 (21.1) | 0 (0) | 0 (0) | 55 (96.5) |
| Tanzania | St Francis RH | NA | NA | NA | NA | NA |
| Tanzania | Mbeya RRH | 24 | 22 (91.7) | 0 (0) | 0 (0) | 2 (8.3) |
| Zimbabwe | Mbare PC | 9 | 9 (100) | 0 (0) | 0 (0) | 0 (0) |
| Zimbabwe | Mutare PH | 86 | 22 (25.6) | 0 (0) | 0 (0) | 86 (100) |
| EMRO |  |  |  |  |  |  |
| Iran | Mahdieh H | 32 | 32 (100) | 0 (0) | 0 (0) | 0 (0) |
| Iran | Shohada TH | 10 | 9 (90) | 1 (10) | 0 (0) | 1 (10) |
| EURO |  |  |  |  |  |  |
| Spain | Castellon GUH | 57 | 7 (12.3) | 1 (1.8) | 1 (1.8) | 57 (100) |
| Spain | Dr Peset UH | 48 | 8 (16.7) | 0 (0) | 0 (0) | 48 (100) |
| SEARO |  |  |  |  |  |  |
| India | JSS H | 9 | 9 (100) | 0 (0) | 0 (0) | 0 (0) |
| India | Grant GMC | 10 | 10 (100) | 0 (0) | 0 (0) | 0 (0) |
| India | IMS SUM H | 24 | 24 (100) | 0 (0) | 0 (0) | 0 (0) |
| India | Kasturba MC | 1 | 1 (100) | 0 (0) | 0 (0) | 0 (0) |
| India | MP Shah MC | 47 | 47 (100) | 0 (0) | 0 (0) | 0 (0) |
| India | SKIMS | 5 | 5 (100) | 0 (0) | 0 (0) | 0 (0) |
| Nepal | Patan H | 66 | 17 (25.8) | 0 (0) | 0 (0) | 66 (100) |
| Nepal | BP Koirala | 24 | 24 (100) | 0 (0) | 0 (0) | 0 (0) |

*Table S4-17. Description of the elements which prevent the level 3 small for gestational age cases to be classified as level 1 or 2*

| Country | Site | Level 3, N | GA information insufficient, n(%*) | Birth weight measurement insufficient, n(%) |
| --- | --- | --- | --- | --- |
| AFRO |  |  |  |  |
| Ghana | Eastern RH | 1 | 1 (100) | 0 (0) |
| Tanzania | Mbeya ZRH | 23 | 23 (100) | 0 (0) |
| Tanzania | Mbeya RRH | 61 | 61 (100) | 0 (0) |
| Zimbabwe | Mbare PC | 60 | 60 (100) | 0 (0) |
| EMRO |  |  |  |  |
| Iran | Mahdieh H | 67 | 67 (100) | 0 (0) |
| SEARO |  |  |  |  |
| India | JSS H | 18 | 18 (100) | 0 (0) |
| India | Grant GMC | 5 | 5 (100) | 0 (0) |
| India | IMS SUM H | 33 | 33 (100) | 0 (0) |
| India | Kasturba MC | 39 | 39 (100) | 0 (0) |
| India | MP Shah MC | 20 | 20 (100) | 0 (0) |
| India | SKIMS | 4 | 4 (100) | 0 (0) |
| Nepal | BP Koirala | 16 | 16 (100) | 0 (0) |

*percentage among all Level 3 cases for this outcome

*Table S4-18. Description of the reasons for recruited stillbirth cases not being classifiable to level 3 using the GAIA definition. For each category, the percentage describes the proportion of unclassified recruited cases for which that was the reason why it could not be classified*

| Country | Site | N unclassified | Signs of life at birth recorded as present, n (% of unclassified) | Unknown whether signs of life at birth present, n (% of unclassified) | The absence of at least one sign of life at birth not recorded, n (% of unclassified) | Inability to distinguish between antepartum and intrapartum stillbirth, n (% of unclassified) | No record of absence of signs of life prior to onset of labour (antepartum) OR no record of evidence of live fetus prior to onset of labour  (intrapartum), n (% of unclassified) | Information on GA insufficient, n (% of unclassified) |
| --- | --- | --- | --- | --- | --- | --- | --- | --- |
| AFRO |  |  |  |  |  |  |  |  |
| Ghana | St Joseph's H | 7 | 0 (0) | 0 (0) | 1 (14.3) | 2 (28.6) | 6 (85.7) | 0 (0) |
| Ghana | Ejisu H | 2 | 0 (0) | 0 (0) | 1 (50) | 0 (0) | 1 (50) | 0 (0) |
| Ghana | Tema GH | 76 | 0 (0) | 1 (1.3) | 1 (1.3) | 2 (2.6) | 74 (97.4) | 17 (22.4) |
| Ghana | Eastern RH | 4 | 0 (0) | 0 (0) | 0 (0) | 0 (0) | 1 (25) | 3 (75) |
| Tanzania | Mbeya ZRH | 21 | 0 (0) | 0 (0) | 0 (0) | 0 (0) | 19 (90.5) | 2 (9.5) |
| Tanzania | St Francis RH | 56 | 0 (0) | 7 (12.5) | 33 (58.9) | 2 (3.6) | 34 (60.7) | 6 (10.7) |
| Tanzania | Mbeya RRH | 65 | 0 (0) | 5 (7.7) | 59 (90.8) | 1 (1.5) | 15 (23.1) | 4 (6.2) |
| Zimbabwe | Mbare PC | 11 | 0 (0) | 0 (0) | 1 (9.1) | 4 (36.4) | 11 (100) | 1 (9.1) |
| Zimbabwe | Mutare PH | 24 | 0 (0) | 0 (0) | 0 (0) | 4 (16.7) | 22 (91.7) | 3 (12.5) |
| EMRO |  |  |  |  |  |  |  |  |
| Iran | Mahdieh H | 51 | 0 (0) | 0 (0) | 0 (0) | 0 (0) | 9 (17.6) | 49 (96.1) |
| Iran | Shohada TH | 14 | 0 (0) | 0 (0) | 1 (7.1) | 2 (14.3) | 6 (42.9) | 10 (71.4) |
| EURO |  |  |  |  |  |  |  |  |
| Spain | Castellon GUH | 1 | 0 (0) | 0 (0) | 0 (0) | 1 (100) | 1 (100) | 1 (100) |
| Spain | Dr Peset UH | NA | NA | NA | NA | NA | NA | NA |
| SEARO |  |  |  |  |  |  |  |  |
| India | JSS H | 20 | 0 (0) | 4 (20) | 16 (80) | 2 (10) | 13 (65) | 3 (15) |
| India | Grant GMC | 51 | 1 (2) | 36 (70.6) | 49 (96.1) | 1 (2) | 27 (52.9) | 10 (19.6) |
| India | IMS SUM H | 3 | 0 (0) | 0 (0) | 0 (0) | 1 (33.3) | 1 (33.3) | 2 (66.7) |
| India | Kasturba MC | 8 | 0 (0) | 0 (0) | 0 (0) | 1 (12.5) | 8 (100) | 0 (0) |
| India | MP Shah MC | 54 | 0 (0) | 2 (3.7) | 3 (5.6) | 8 (14.8) | 54 (100) | 2 (3.7) |
| India | SKIMS | 15 | 0 (0) | 8 (53.3) | 8 (53.3) | 4 (26.7) | 11 (73.3) | 7 (46.7) |
| Nepal | Patan H | 8 | 0 (0) | 0 (0) | 2 (25) | 1 (12.5) | 2 (25) | 5 (62.5) |
| Nepal | BP Koirala | 15 | 0 (0) | 2 (13.3) | 13 (86.7) | 0 (0) | 1 (6.7) | 0 (0) |

*Table S4-19. Description of the elements which prevent the level 3 stillbirth cases being classified as level 1 or 2*

| Country | Site | Level 3, N | GA information insufficient, n (%*) | Insufficient information on cardiac activity before labour, n (%) | Insufficient information on fetal movement before labour, n (%) |
| --- | --- | --- | --- | --- | --- |
| AFRO |  |  |  |  |  |
| Ghana | St Joseph's H | 10 | 6 (60) | 4 (40) | 4 (40) |
| Ghana | Ejisu H | 5 | 1 (20) | 3 (60) | 4 (80) |
| Ghana | Tema GH | 22 | 18 (81.8) | 20 (90.9) | 18 (81.8) |
| Ghana | Eastern RH | 26 | 23 (88.5) | 4 (15.4) | 4 (15.4) |
| Tanzania | Mbeya ZRH | 70 | 68 (97.1) | 7 (10) | 7 (10) |
| Tanzania | St Francis RH | 43 | 43 (100) | 13 (30.2) | 13 (30.2) |
| Tanzania | Mbeya RRH | 20 | 18 (90) | 8 (40) | 12 (60) |
| Zimbabwe | Mbare PC | 4 | 4 (100) | 4 (100) | 2 (50) |
| Zimbabwe | Mutare PH | 24 | 24 (100) | 7 (29.2) | 5 (20.8) |
| EMRO |  |  |  |  |  |
| Iran | Mahdieh H | 34 | 34 (100) | 2 (5.9) | 21 (61.8) |
| Iran | Shohada TH | 5 | 3 (60) | 1 (20) | 2 (40) |
| EURO |  |  |  |  |  |
| Spain | Castellon GUH | NA | NA | NA | NA |
| Spain | Dr Peset UH | NA | NA | NA | NA |
| SEARO |  |  |  |  |  |
| India | JSS H | 1 | 1 (100) | 1 (100) | 1 (100) |
| India | Grant GMC | NA | NA | NA | NA |
| India | IMS SUM H | 21 | 21 (100) | 0 (0) | 0 (0) |
| India | Kasturba MC | 11 | 10 (90.9) | 5 (45.5) | 3 (27.3) |
| India | MP Shah MC | 19 | 13 (68.4) | 10 (52.6) | 8 (42.1) |
| India | SKIMS | 7 | 7 (100) | 0 (0) | 0 (0) |
| Nepal | Patan H | 3 | 3 (100) | 0 (0) | 0 (0) |
| Nepal | BP Koirala | 23 | 23 (100) | 0 (0) | 0 (0) |

*Table S4-20. Description of the reasons for recruited congenital microcephaly cases not being classifiable to level 3 using the GAIA definition. For each category, the percentage describes the proportion of unclassified recruited cases for which that was the reason why it could not be classified.*

| Country | Site | N unclassified | GA <24 weeks, n (% of unclassified) | Information on GA insufficient, n (% of unclassified) | HC not 2SD below mean or 3^rd^ percentile according to GA and gender on Intergrowth-21 chart, n (% of unclassified) | HC measured >6 weeks after birth, n (% of unclassified) | HC not available | Not diagnosed based on ICD code algorithm, n (% of unclassified) |
| --- | --- | --- | --- | --- | --- | --- | --- | --- |
| AFRO |  |  |  |  |  |  |  |  |
| Ghana | Tema GH | 3 | 0 (0) | 3 (100) | 0 (0) | 0 (0) | 0 (0) | 3 (100) |
| Ghana | Eastern RH | 2 | 0 (0) | 2 (100) | 0 (0) | 0 (0) | 0 (0) | 2 (100) |
| Tanzania | Mbeya ZRH | 1 | 0 (0) | 0 (0) | NA (NA) | 1 (100) | 1 (100) | 1 (100) |
| Tanzania | Mbeya RRH | 1 | 0 (0) | 0 (0) | 1 (100) | 0 (0) | 0 (0) | 1 (100) |
| EMRO |  |  |  |  |  |  |  |  |
| Iran | Mahdieh H | 8 | 0 (0) | 7 (87.5) | 2 (25) | 0 (0) | 0 (0) | 8 (100) |
| Iran | Shohada TH | 7 | 0 (0) | 7 (100) | 4 (57.1) | 0 (0) | 0 (0) | 7 (100) |
| EURO |  |  |  |  |  |  |  |  |
| Spain | Castellon GUH | 6 | 0 (0) | 1 (16.7) | 5 (83.3) | 0 (0) | 0 (0) | 6 (100) |
| Spain | Dr Peset UH | 11 | 0 (0) | 2 (18.2) | 10 (90.9) | 0 (0) | 0 (0) | 11 (100) |
| SEARO |  |  |  |  |  |  |  |  |
| India | JSS H | 1 | 0 (0) | 0 (0) | 1 (100) | 0 (0) | 0 (0) | 1 (100) |
| India | Kasturba MC | 1 | 0 (0) | 0 (0) | 1 (100) | 0 (0) | 0 (0) | 1 (100) |
| India | MP Shah MC | 3 | 0 (0) | 2 (66.7) | 1 (33.3) | 0 (0) | 0 (0) | 3 (100) |
| Nepal | Patan H | 2 | 0 (0) | 0 (0) | 2 (100) | 0 (0) | 0 (0) | 2 (100) |
| Nepal | BP Koirala | 1 | 0 (0) | 0 (0) | 1 (100) | 0 (0) | 0 (0) | 1 (100) |

GA: Gestational Age; HC: head circumference; NA: not applicable

*Table S4-21. Congenital microcephaly: Description of the elements which prevent the level 3 congenital microcephaly cases from being classified as level 1 or 2*

| Country | Site | N classified as Level 3 | Level 1/2: GA information insufficient | Level 1/2: No record of HC to compare to relevant charts | |
| --- | --- | --- | --- | --- | --- |
| AFRO |  |  |  |  |  |
| Tanzania | Mbeya RRH | 1 | 1 (100) | 0 (0) |  |
| EMRO |  |  |  |  |  |
| Iran | Mahdieh H | 3 | 3 (100) | 0 (0) |  |
| EURO |  |  |  |  |  |
| Spain | Castellon GUH | 1 | 1 (100) | 0 (0) |  |
| Spain | Dr Peset UH | 2 | 2 (100) | 0 (0) |  |
| SEARO |  |  |  |  |  |
| India | JSS H | 1 | 1 (100) | 0 (0) |  |
| India | IMS SUM H | 2 | 2 (100) | 0 (0) |  |
| India | Kasturba MC | 2 | 2 (100) | 0 (0) |  |
| India | SKIMS | 1 | 1 (100) | 0 (0) |  |
| Nepal | BP Koirala | 7 | 7 (100) | 0 (0) |  |

*Table S4-22. Neonatal invasive bloodstream infection: Description of the elements which prevent the GAIA level 3 neonatal invasive bloodstream infection cases to be classified as level 1 or 2.*

| Country | Site | Level 3, N | No sample from normally sterile site tested, n (%*) | Negative or insufficient findings from sample from normally sterile site, n (%) | No validated laboratory method used**, n (%) | Insufficient clinical criteria met, n (%) |
| --- | --- | --- | --- | --- | --- | --- |
| AFRO |  |  |  |  |  |  |
| Ghana | St Joseph's H | 6 | 6 (100) | 0 (0) | 0 (0) | 6 (100) |
| Ghana | Ejisu H | 1 | 1 (100) | 0 (0) | 0 (0) | 1 (100) |
| Ghana | Tema GH | 8 | 6 (75) | 2 (25) | 0 (0) | 8 (100) |
| Ghana | Eastern RH | 3 | 3 (100) | 0 (0) | 0 (0) | 3 (100) |
| Tanzania | Mbeya ZRH | 5 | 5 (100) | 0 (0) | 0 (0) | 5 (100) |
| Tanzania | St Francis RH | 10 | 10 (100) | 0 (0) | 0 (0) | 10 (100) |
| Tanzania | Mbeya RRH | 15 | 15 (100) | 0 (0) | 0 (0) | 15 (100) |
| EMRO |  |  |  |  |  |  |
| Iran | Shohada TH | 5 | 5 (100) | 0 (0) | 0 (0) | 5 (100) |
| SEARO |  |  |  |  |  |  |
| India | MP Shah MC | 2 | 2 (100) | 0 (0) | 0 (0) | 2 (100) |
| Nepal | Patan H | 1 | 1 (100) | 0 (0) | 0 (0) | 1 (100) |

*Table S4-23. Neonatal meningitis: Description of the reasons for recruited neonatal meningitis cases not being classifiable to level 3 using the GAIA definition. For each category, the percentage describes the proportion of unclassified recruited cases for which that was the reason why it could not be classified*

| Country | Site | N unclassified | Fever criterion not met | Insufficient number of other clinical criteria met |
| --- | --- | --- | --- | --- |
| Ghana | Eastern RH | 2 | 0 (0) | 2 (100) |
| Tanzania | Mbeya ZRH | 2 | 0 (0) | 2 (100) |
| Tanzania | St Francis RH | 4 | 0 (0) | 4 (100) |
| Tanzania | Mbeya RRH | 2 | 1 (50) | 2 (100) |
| India | JSS H | 2 | 2 (100) | 2 (100) |
| India | Grant GMC | 2 | 1 (50) | 2 (100) |
| India | IMS SUM H | 3 | 2 (66.7) | 3 (100) |
| India | Kasturba MC | 1 | 1 (100) | 1 (100) |
| India | MP Shah MC | 1 | 0 (0) | 1 (100) |
| Iran | Shohada TH | 1 | 1 (100) | 1 (100) |
| Nepal | Patan H | 22 | 10 (45.5) | 22 (100) |
| Nepal | BP Koirala | 7 | 2 (28.6) | 7 (100) |

*Table S4-24. Neonatal meningitis: Description of the elements which prevent the level 3 neonatal meningitis cases being classified as level 1 or 2.*

| Country | Site | N classified as Level 3 | No sample from normally sterile site tested | Negative or insufficient findings from sample from normally sterile site | No Validated laboratory method used | CSF pleocytosis of positive IgM antibodies not reported or absent | Insufficient clinical criteria met |
| --- | --- | --- | --- | --- | --- | --- | --- |
| AFRO |  |  |  |  |  |  |  |
| Ghana | Eastern RH | 1 | 0 (0) | 1 (100) | 0 (0) | 1 (100) | 0 (0) |
| SEARO |  |  |  |  |  |  |  |
| Nepal | BP Koirala | 8 | 1 (12.5) | 7 (87.5) | 0 (0) | 0 (0) | 0 (0) |

*Table S4-25. Description of the elements which prevent the level 3 neonatal respiratory infection cases to be classified as level 1 or 2*

| Country | Site | Level 3, N | No sample tested, n (%*) | Negative or insufficient findings from sample, n (%) | No validated laboratory method used**, n (%) | No chest X-ray done, n (%) | No relevant findings on chest X-ray, n (%) | Clinical criteria insufficient, n (%) |
| --- | --- | --- | --- | --- | --- | --- | --- | --- |
| AFRO |  |  |  |  |  |  |  |  |
| Ghana | St Joseph's H | 8 | 7 (87.5) | 1 (12.5) | 0 (0) | 8 (100) | 0 (0) | 1 (12.5) |
| Ghana | Tema GH | 5 | 3 (60) | 0 (0) | 0 (0) | 5 (100) | 0 (0) | 1 (20) |
| Tanzania | Mbeya ZRH | 1 | 1 (100) | 0 (0) | 0 (0) | 1 (100) | 0 (0) | 0 (0) |
| Tanzania | St Francis RH | 3 | 3 (100) | 0 (0) | 0 (0) | 3 (100) | 0 (0) | 0 (0) |
| Tanzania | Mbeya RRH | 2 | 2 (100) | 1 (50) | 0 (0) | 2 (100) | 0 (0) | 1 (50) |
| Zimbabwe | Mutare PH | 1 | 0 (0) | 0 (0) | 0 (0) | 1 (100) | 0 (0) | 0 (0) |
| EMRO |  |  |  |  |  |  |  |  |
| Iran | Shohada TH | 3 | 0 (0) | 0 (0) | 0 (0) | 0 (0) | 1 (33.3) | 1 (33.3) |
| SEARO |  |  |  |  |  |  |  |  |
| India | JSS H | 3 | 2 (66.7) | 0 (0) | 0 (0) | 0 (0) | 2 (66.7) | 0 (0) |
| India | Kasturba MC | 1 | 0 (0) | 0 (0) | 0 (0) | 0 (0) | 1 (100) | 0 (0) |
| India | MP Shah MC | 1 | 0 (0) | 0 (0) | 0 (0) | 0 (0) | 0 (0) | 0 (0) |
| Nepal | Patan H | 51 | 1 (2) | 4 (7.8) | 1 (2) | 0 (0) | 28 (54.9) | 26 (51) |
| Nepal | BP Koirala | 1 | 0 (0) | 0 (0) | 0 (0) | 0 (0) | 0 (0) | 0 (0) |

*Table S4-26. Maternal immunization: Description of the elements which prevent level 3 maternal immunization during pregnancy to be classified as level 1 or 2.*

| Country | Site | Level 3, N* | Immunization not obtained from a primary source medical record, n(%*) | Date of immunization not available, n(%) | Time of immunization not available, n(%)* | Batch number not available, n(%)** | Disease against which was vaccinated not available, n(%)** | Vaccine brand name not available, n(%)** | Date of immunization (when available) not during pregnancy, n(%) |
| --- | --- | --- | --- | --- | --- | --- | --- | --- | --- |
| AFRO |  |  |  |  |  |  |  |  |  |
| Ghana | St Joseph's H | 2 | 0 (0) | 1 (50) | 2 (100) | 2 (100) | 0 (0) | 2 (100) | 1 (50) |
| Ghana | Ejisu H | 1 | 0 (0) | 0 (0) | 0 (0) | 0 (0) | 0 (0) | 0 (0) | 1 (100) |
| Ghana | Tema GH | 7 | 0 (0) | 6 (85.7) | 7 (100) | 4 (57.1) | 0 (0) | 2 (28.6) | 1 (14.3) |
| Ghana | Eastern RH | 8 | 0 (0) | 3 (37.5) | 7 (87.5) | 8 (100) | 0 (0) | 8 (100) | 5 (62.5) |
| Tanzania | Mbeya ZRH | 14 | 7 (50) | 12 (85.7) | 13 (92.9) | 14 (100) | 0 (0) | 14 (100) | 1 (7.1) |
| Tanzania | St Francis RH | 29 | 0 (0) | 1 (3.4) | 29 (100) | 29 (100) | 0 (0) | 29 (100) | 28 (96.6) |
| Tanzania | Mbeya RRH | 27 | 1 (3.7) | 9 (33.3) | 27 (100) | 27 (100) | 0 (0) | 27 (100) | 18 (66.7) |
| Zimbabwe | Mbare PC | 14 | 0 (0) | 11 (78.6) | 14 (100) | 14 (100) | 0 (0) | 14 (100) | 3 (21.4) |
| Zimbabwe | Mutare PH | 27 | 7 (25.9) | 15 (55.6) | 27 (100) | 27 (100) | 0 (0) | 27 (100) | 10 (37) |
| EMRO |  |  |  |  |  |  |  |  |  |
| Iran | Mahdieh H | 17 | 15 (88.2) | 16 (94.1) | 17 (100) | 17 (100) | 0 (0) | 17 (100) | 0 (0) |
| EURO |  |  |  |  |  |  |  |  |  |
| Spain | Castellon GUH | 28 | 27 (96.4) | 2 (7.1) | 28 (100) | 2 (7.1) | 0 (0) | 4 (14.3) | 1 (3.6) |
| Spain | Dr Peset UH | 59 | 26 (44.1) | 59 (100) | 59 (100) | 52 (88.1) | 0 (0) | 58 (98.3) | 0 (0) |
| SEARO |  |  |  |  |  |  |  |  |  |
| India | JSS H | 176 | 175 (99.4) | 174 (98.9) | 176 (100) | 176 (100) | 0 (0) | 176 (100) | 0 (0) |
| India | Grant GMC | 62 | 58 (93.5) | 56 (90.3) | 62 (100) | 62 (100) | 0 (0) | 62 (100) | 0 (0) |
| India | IMS SUM H | 149 | 149 (100) | 148 (99.3) | 149 (100) | 149 (100) | 0 (0) | 149 (100) | 0 (0) |
| India | Kasturba MC | 187 | 185 (98.9) | 126 (67.4) | 187 (100) | 187 (100) | 0 (0) | 187 (100) | 0 (0) |
| India | MP Shah MC | 210 | 15 (7.1) | 205 (97.6) | 203 (96.7) | 210 (100) | 0 (0) | 210 (100) | 5 (2.4) |
| India | SKIMS | 2 | 1 (50) | 1 (50) | 2 (100) | 1 (50) | 0 (0) | 2 (100) | 0 (0) |
| Nepal | Patan H | 89 | 6 (6.7) | 85 (95.5) | 89 (100) | 89 (100) | 0 (0) | 89 (100) | 1 (1.1) |
| Nepal | BP Koirala | 366 | 366 (100) | 366 (100) | 366 (100) | 366 (100) | 0 (0) | 366 (100) | 0 (0) |


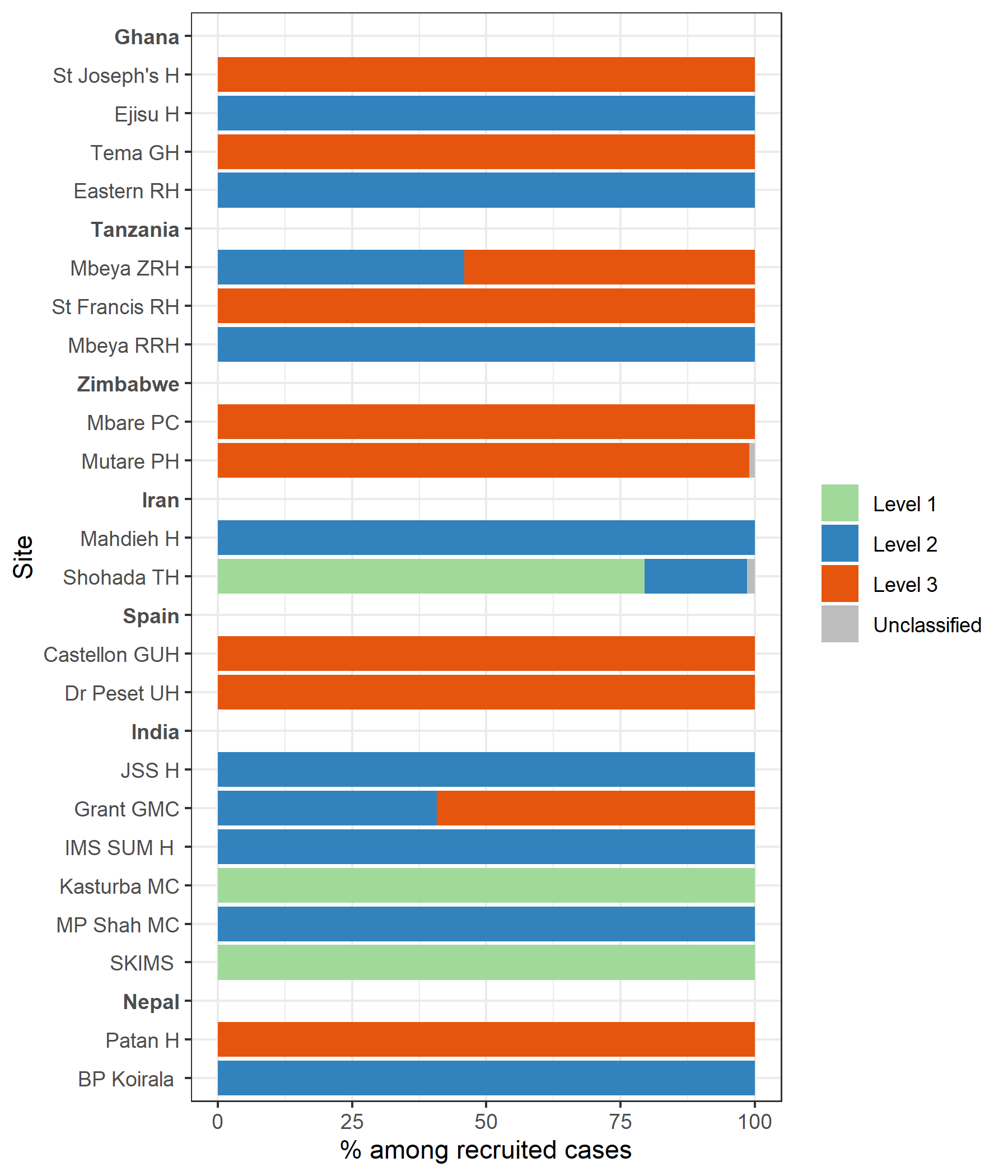

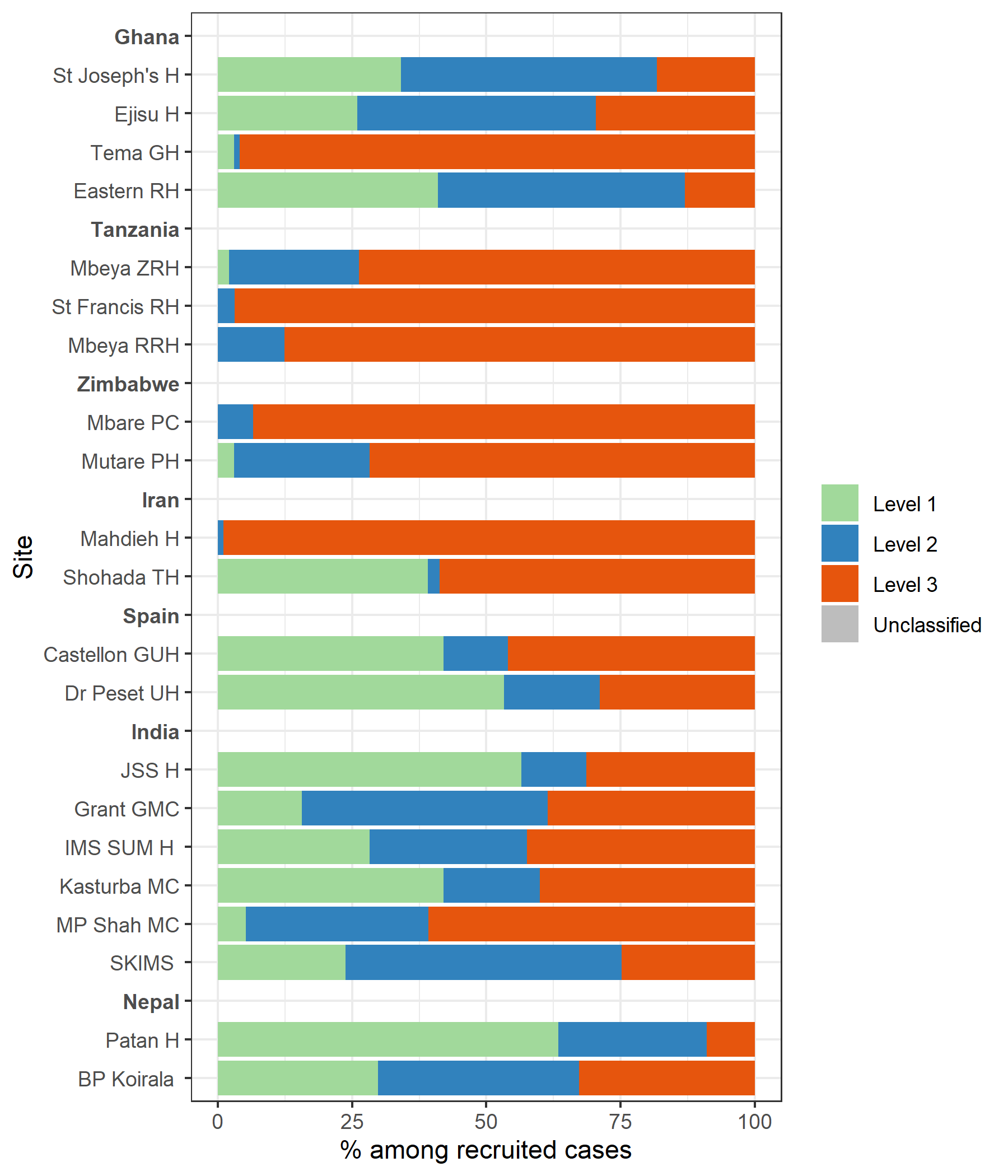


(a)

(b)

**Supp.Fig 1.** **(a)Low Birthweight and (b) Preterm birth case classification according to the GAIA definition, as % of recruited cases**


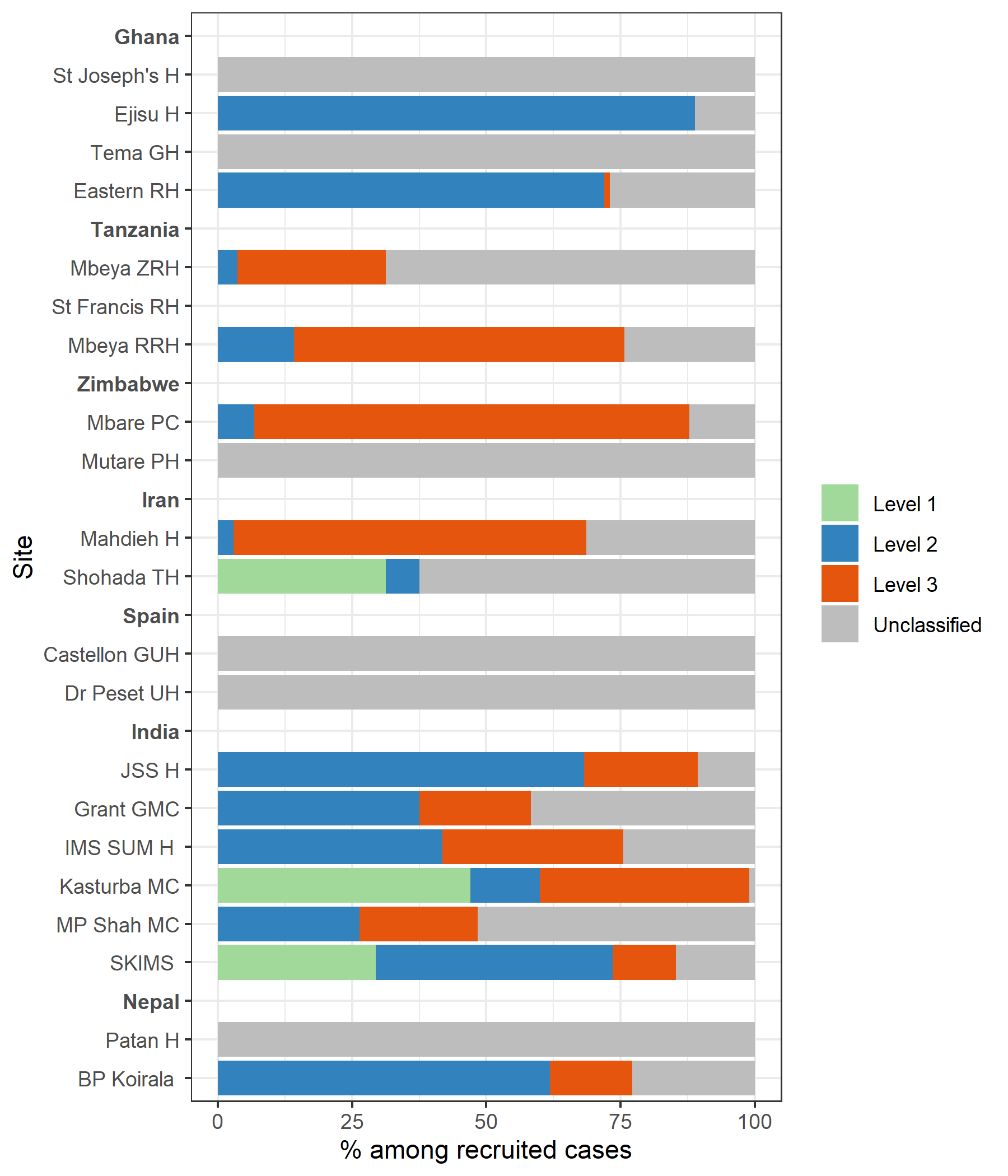

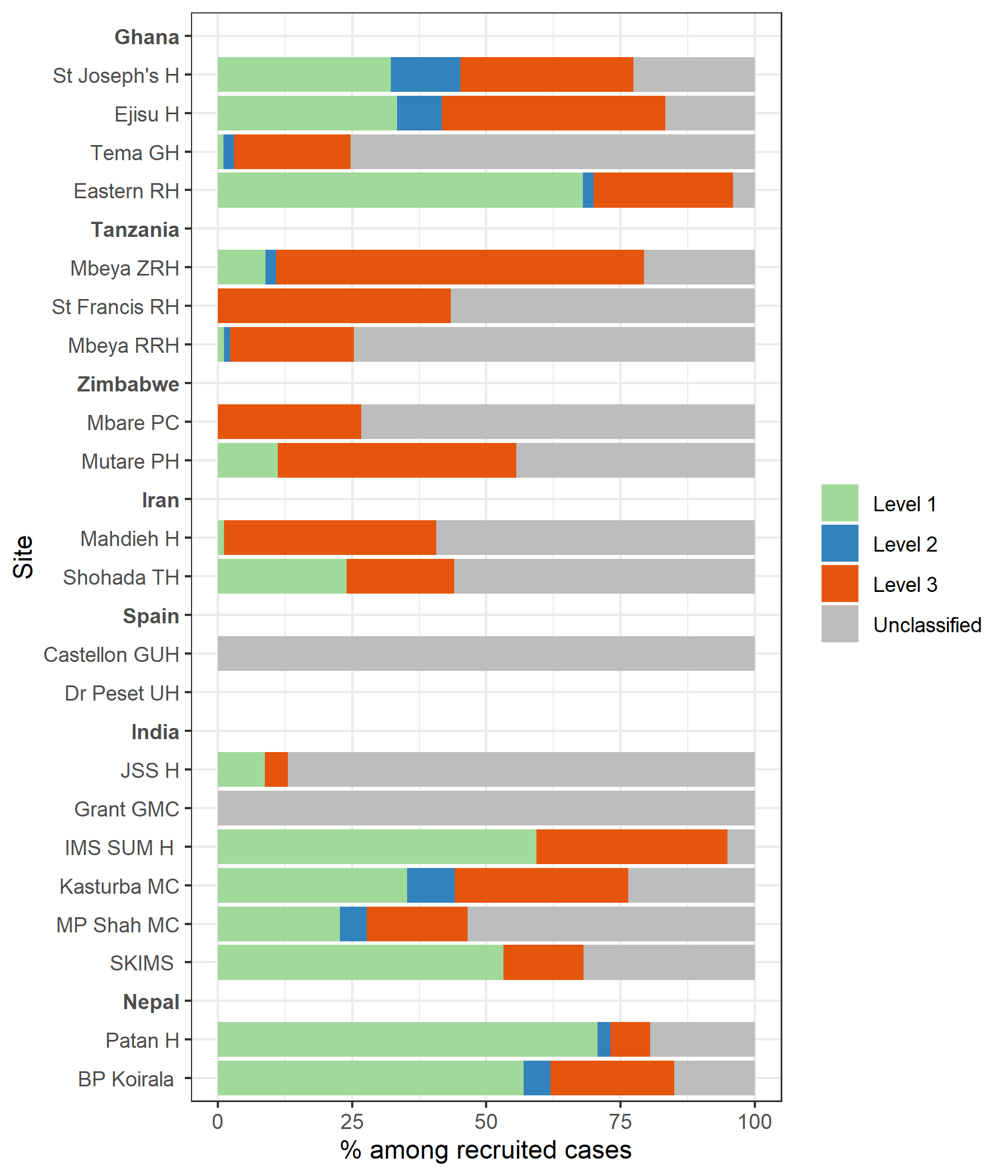


(a)

(b)

**Supp.Fig.2: (a)Small for gestational age and (b) Stillbirth case classification according to the GAIA definition, as % of recruited cases**


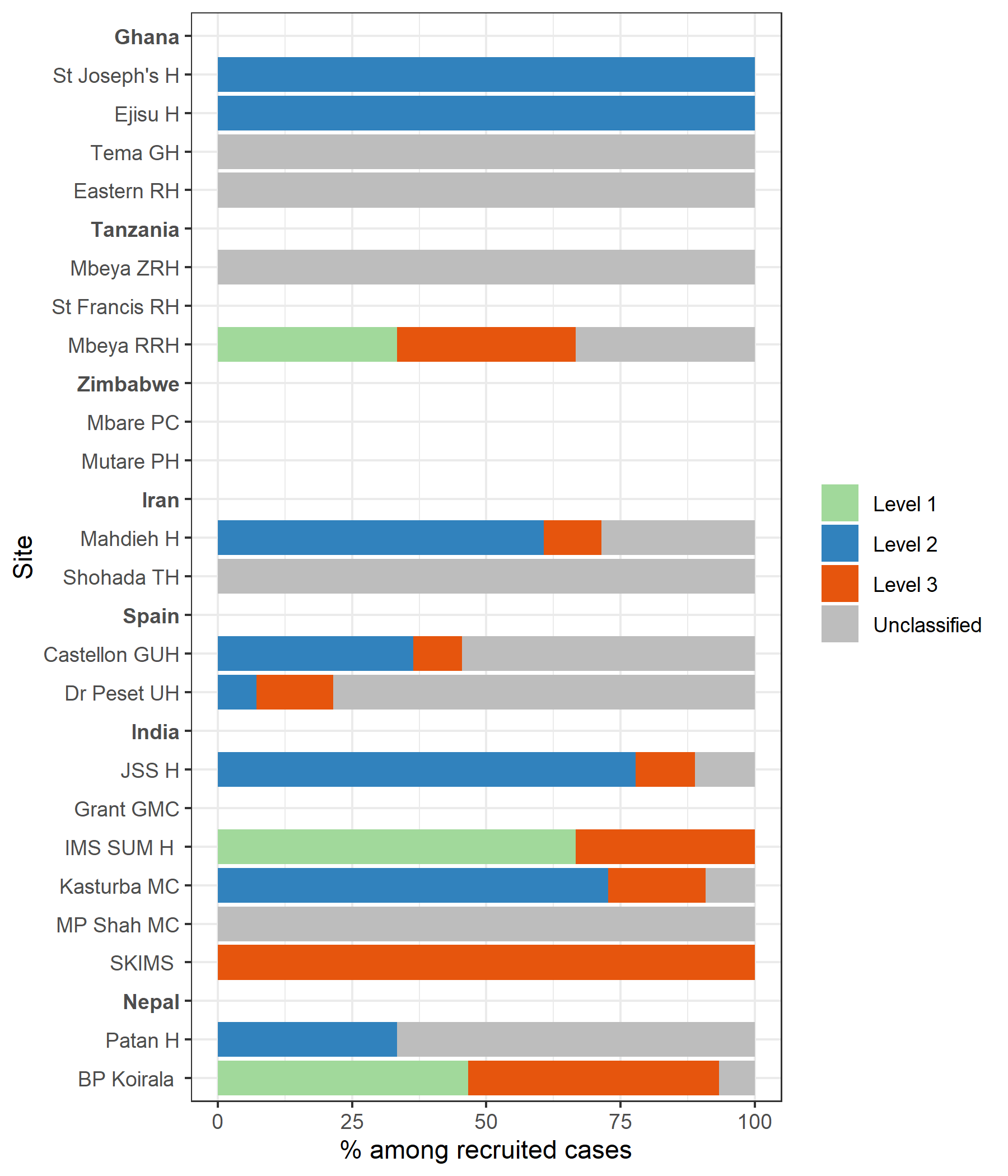

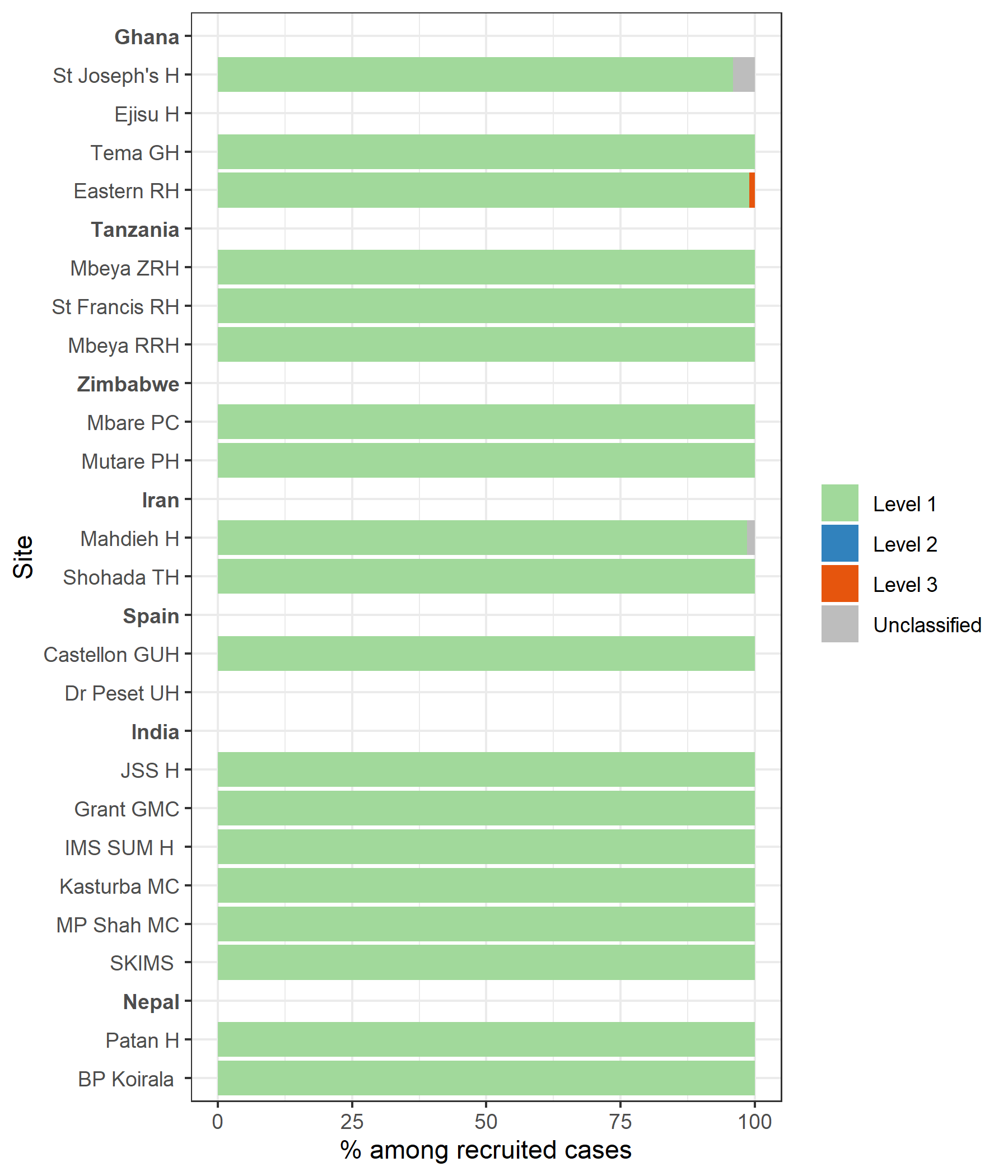


(a)

(b)

**Supp.Fig 3. (a) Congenital microcephaly and (b) Neonatal death case classification according to the GAIA definition, as % of recruited cases**


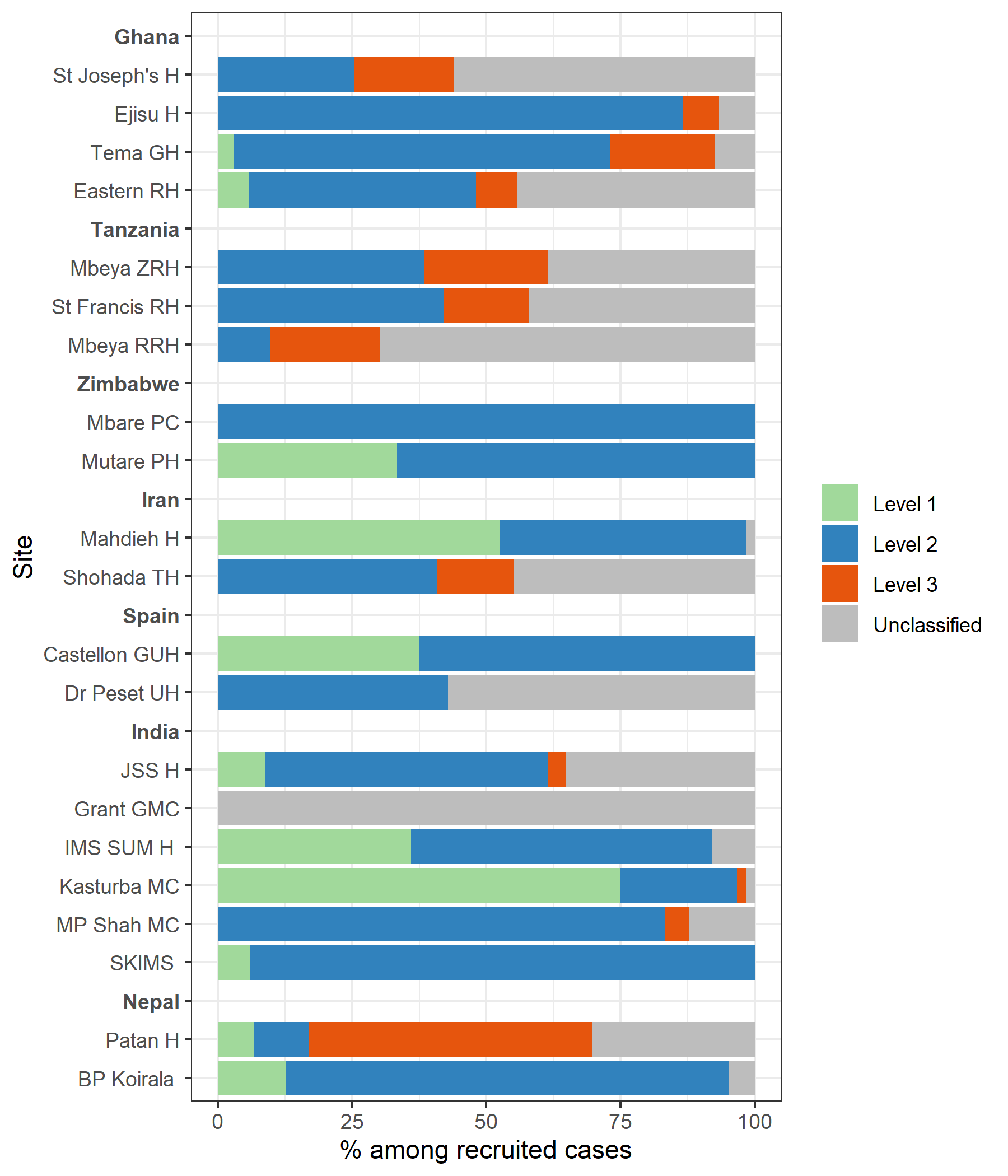


(a)

(b)


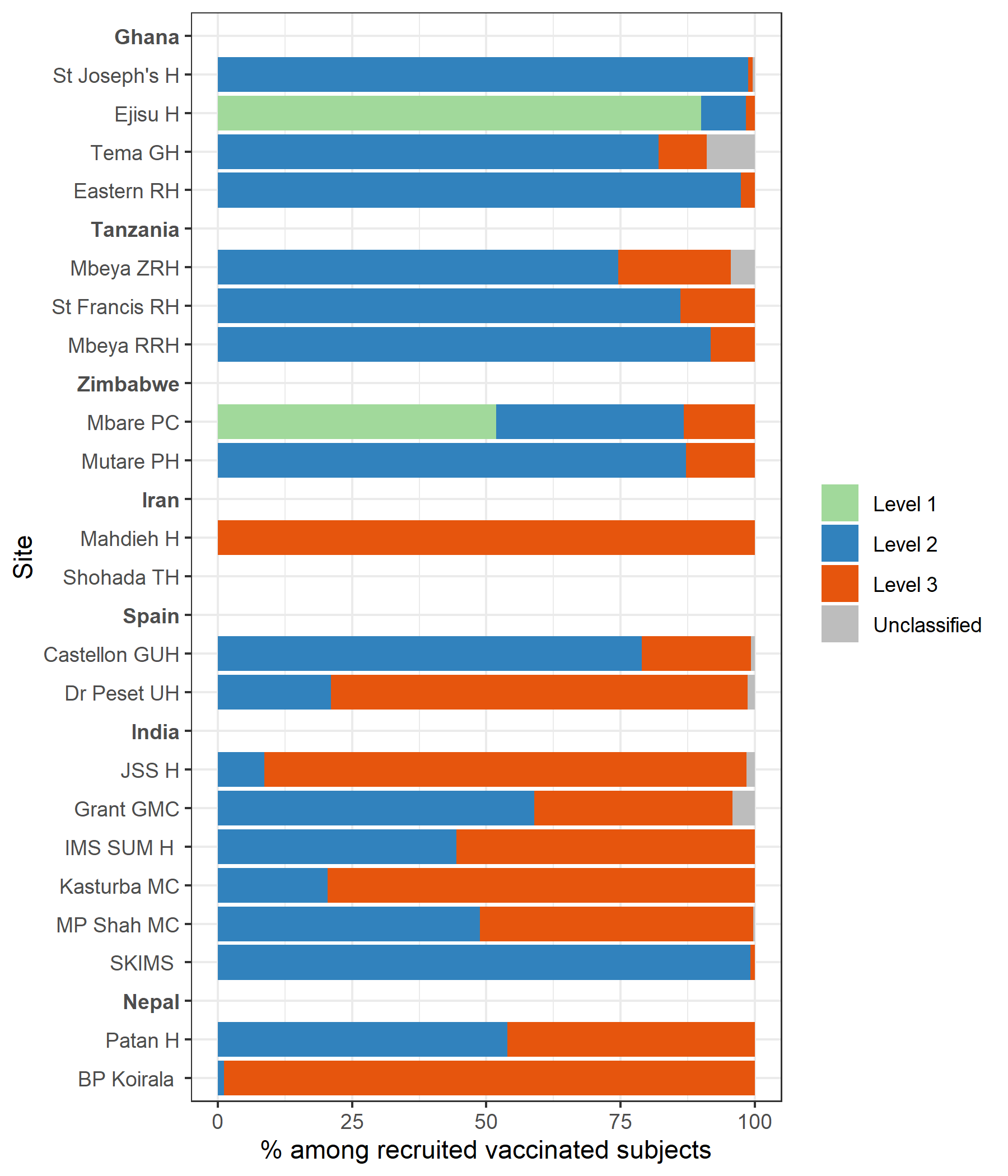


**Supp.Fig.4. (a) Neonatal Infection and (b) Maternal Immunization case classification according to the GAIA definition, as % of recruited cases**
